# Roux-en-Y gastric bypass, adjustable gastric banding, or sleeve gastrectomy for severe obesity (By-Band-Sleeve): a multicentre, open label, three-group, randomised controlled trial

**DOI:** 10.1016/S2213-8587(25)00025-7

**Published:** 2025-03-31

**Authors:** The By-Band-Sleeve Collaborative Group, Sanjay Agrawal, Ahmed Ahmed, Robert C Andrews, Jane M Blazeby, Natalie Blencowe, James P Byrne, Nicholas Carter, Katy Chalmers, Sian Cousins, Karen Coulman, Lucy Culliford, Lucy Dabner, Nick Davies, Jenny L Donovan, Danielle Edwards, Rebecca Evans, Jilles M Fermont, Ian Finlay, Eleanor A Gidman, Jeremy Hayden, St James, James Hopkins, Neil Jennings, Sofia Kanavou, Rositsa Koleva-Kolarova, Paul Leeder, Rachel Maishman, Graziella Mazza, Mary O’Kane, Katie Pike, Sangeetha Paramasivan, Koen Pouwels, Barnaby C Reeves, Alba X Realpe, Chris A Rogers, Nicki Salter, Rishi Singhal, Janice L Thompson, Richard Welbourn, Caroline Wilson, Paul Whybrow, Sarah Wordsworth, Other study group members, Research nurses, practitioners and dieticians, Sally Abbott, Benita Adams, Paul Corrigan, Sabrina Dabner, Tracey Fong, Hassina Furreed, Jeremy Gilbert, Kirsty Gladas, Beth Greenslade, Jennifer Henderson, Helen Horton, Chiwen Lin, Amy Long, Priya Mathew, Sarah Matthias, Maria Moon, Catherine Moriarty, Mark Priestley, Pedro B Sardo, Kerry Thorpe, Jill Townley, Rachael Wright, Bariatric surgeons, Hazem Al Momani, Waleed Al-Khyatt, Omer Altaan, Sherif Awad, Altaf Awan, Shlok Balupuri, Richard Byrom, Vasileios Charalampakis, James Clark, Michael Clarke, Allwyn Cota, Markos Daskalakis, Simon Dexter, Khaleel Fareed, Sherif Hakky, James Hewes, Marianne Hollyman, Buddika Jayathilaka, Jamie Kelly, Ben Knight, John Loy, Brijesh Madhok, Kamal Mahawar, David Mahon, Matthew Mason, Samir Mehta, Peter Mekhail, Simon Monkhouse, Krishna Moorthy, Rajwinder Nijjar, Hamish Noble, Alan Osborne, Dimitri Pournaras, Sanjay Purkayastha, Martin Richardson, Andrew G Robertson, Abeezar Sarela, Peter Small, Shaw Somers, Paul Super, Christos Tsironis, James Williamson, Oversight committees: Data Monitoring Committee, Nick Finer, Olbers Torsten, Craig Ramsay, Trial Steering Committee, Julia Brown, Jo Coast, John Dixon, Jodie Henman, Charlotte McCaie, Steve Morris, Sally Norton, Michel Suter, John Wilding, Patient and public advisors, John Bessent, Caroline Clay, Janet Edmond, Support staf, Manuela Antognozzi, Rachel Brierley, Anna Gilbert, Rachael Heys, Surinder Kaur, Jenny Lamb, Holly Mckeon, Alexander Mikulski, Abby O’Connell, Stephen Palmer, Jade Salter-Hewitt, Funding support, Paul Roderick, IT support staff, Samir Bellani, Jonathan Betts, Heike Cappel-Porter, Neil Smith

**Affiliations:** Homerton University Hospital, London, UK; Imperial College, London, UK; University of Exeter Medical School, UK; NIHR Bristol and Weston Biomedical Research Centre, Bristol Medical School, Population Health Sciences, Professor University of Bristol, UK; NIHR Bristol and Weston Biomedical Research Centre, Bristol Medical School, Population Health Sciences, University of Bristol, UK; University Hospital Southampton NHS Foundation Trust, UK; Portsmouth Hospitals University NHS Trust, UK; Population Health Sciences, University of Bristol, UK; NIHR Bristol and Weston Biomedical Research Centre, Bristol Medical School, Population Health Sciences, Professor University of Bristol, UK; Population Health Sciences, University of Bristol, UK; Bristol Trials Centre, Bristol Medical School, University of Bristol, UK; Bristol Trials Centre, Bristol Medical School, University of Bristol, UK; Royal Bournemouth and Christchurch NHS Foundation Trust, UK; Bristol Medical School, University of Bristol, UK; Bristol Trials Centre, Bristol Medical School, University of Bristol, UK; Bristol Trials Centre, Bristol Medical School, University of Bristol, UK; Health Economics Research Centre, Nuffield Department of Population Health, University of Oxford, UK; Royal Cornwall NHS Foundation Trust, Truro, UK; Bristol Trials Centre, Bristol Medical School, University of Bristol, UK; University Hospital Leeds, UK; North Bristol NHS Trust, Bristol, UK; South Tyneside and Sunderland NHS Foundation Trust, UK; Bristol Trials Centre, Bristol Medical School, University of Bristol, UK; Health Economics Research Centre, Nuffield Department of Population Health, University of Oxford, UK; University Hospitals of Derby and Burton, UK; Bristol Trials Centre, Bristol Medical School, University of Bristol, UK; Bristol Trials Centre, Bristol Medical School, University of Bristol, UK; Dietetic Department, Leeds Teaching Hospitals NHS Trust, UK; Bristol Trials Centre, Bristol Medical School, University of Bristol, UK; Bristol Medical School, University of Bristol, Bristol, UK; Health Economics Research Centre, Nuffield Department of Population Health, University of Oxford, UK; Bristol Trials Centre, Bristol Medical School, University of Bristol, UK; Bristol Medical School, University of Bristol, UK; Bristol Trials Centre, Bristol Medical School, University of Bristol, UK; Somerset NHS Foundation Trust, UK; Heart of England NHS Foundation Trust, UK; School of Sport, Exercise & Rehabilitation Sciences, University of Birmingham, UK; Somerset NHS Foundation Trust, UK; Bristol Medical School, University of Bristol, UK; Bristol Medical School, University of Bristol, UK; Health Economics Research Centre, Nuffield Department of Population Health, University of Oxford, UK

## Abstract

**Background:**

The health risks of severe obesity can be reduced with metabolic and bariatric surgery, but it is uncertain which operation is most effective or cost-effective. We aimed to compare Roux-en-Y gastric bypass, adjustable gastric banding, and sleeve gastrectomy in patients with severe obesity.

**Methods:**

By-Band-Sleeve is a pragmatic, multi-centre, open-label, randomised controlled trial conducted in 12 hospitals in the UK. Eligible participants were adults (aged ≥18 years) meeting national criteria for metabolic and bariatric surgery. Initially, a 2-group trial (Roux-en-Y gastric bypass versus adjustable gastric banding) became a 3-group trial to include sleeve gastrectomy at 2·6 years from study opening, when it became widely used in the UK. Co-primary endpoints were weight (proportion achieving ≥50% excess weight loss) and quality-of-life (EQ-5D utility score) at 3 years. If the proportion achieving at least 50% excess weight loss was non-inferior (<12% difference between groups) and quality-of-life was superior, sleeve gastrectomy and Roux-en-Y gastric bypass were considered more effective than adjustable gastric banding, and sleeve gastrectomy more effective than Roux-en-Y gastric bypass. Cost-effectiveness of the procedures was compared. This trial is registered with ClinicalTrials.gov, NCT02841527, and ISRCTN, 00786323.

**Results:**

Between Jan 16, 2013, and Sept 27, 2019, 1351 participants were randomly assigned; five withdrew consent and 1346 (mean age 47·3 [SD 10·6] years, 1020 [76%] women, 324 (24%) men, and two with missing data, mean weight of 129·7 kg [23·6] and mean BMI of 46·4 [6·9] kg/m^2^) were included in this report. Of 1346 participants, 462 (34%) were in the Roux-en-Y gastric bypass group, 464 (34%) in the adjustable gastric banding group, and 420 (31%) in the sleeve gastrectomy group. 1183 (88%) participants underwent surgery. 276 (68%) of 405 participants in the Roux-en-Y gastric bypass group, 97 (25%) of 383 participants in the adjustable gastric banding group and 141 (41%) of 342 participants in the sleeve gastrectomy group achieved at least 50% excess weight loss (adjusted risk difference: Roux-en-Y gastric bypass *vs* adjustable gastric banding 41% [98% CI 34 to 48]; sleeve gastrectomy *vs* adjustable gastric banding 15% [5 to 24]; sleeve gastrectomy *vs* Roux-en-Y gastric bypass, –26% [–36 to –16%]). Mean EQ-5D scores were 0·72 for Roux-en-Y gastric bypass, 0·62 for adjustable gastric banding, and 0·68 for sleeve gastrectomy (adjusted mean difference: Roux-en-Y gastric bypass *vs* adjustable gastric banding 0·08 [0·04 to 0·12], sleeve gastrectomy *vs* adjustable gastric banding 0·05 [0·01 to 0·09], and sleeve gastrectomy *vs* Roux-en-Y gastric bypass –0·03 [–0·07 to 0·01]). 1651 adverse events were reported following surgery (5·7 per year after sleeve gastrectomy, 6·0 per year after Roux-en-Y gastric bypass, and 4·6 per year after adjustable gastric banding). There were 11 deaths from randomisation to 3 years: one attributable to surgery (in the adjustable gastric bypass group, during the surgical admission) and ten not attributable to surgery (four each in the Roux-en-Y gastric bypass and adjustable gastric banding groups and two in the sleeve gastrectomy group). Roux-en-Y gastric bypass was most cost-effective.

**Interpretation:**

Roux-en-Y gastric bypass and sleeve gastrectomy are more effective than adjustable gastric banding. Sleeve gastrectomy has inferior weight loss and lower mean quality of life score compared with Roux-en-Y gastric bypass. Based on this evidence, it is recommended that patients electing to have metabolic and bariatric surgery are advised to have Roux-en-Y gastric bypass. Where contraindicated or unfeasible, sleeve gastrectomy should be offered. This evidence does not support adjustable gastric band as standard treatment for severe obesity.

**Funding:**

National Institute for Health and Care Research Health Technology Assessment Programme.

## Introduction

Global rates of obesity (defined as a BMI ≥30 kg/m^2^)^1^ and severe obesity (defined as a BMI ≥35 kg/m^2^), are increasing, with over 25% of the world’s population predicted to be affected by 2035.^2^ As an elevated BMI is strongly associated with multiple diseases, effective preventative and treatment interventions are important. Lifestyle interventions form the basis of obesity management but if these prove ineffective, metabolic and bariatric surgery or obesity medications might be offered. Metabolic and bariatric surgery can lead to 20–30% total weight loss. Trials show that obesity management medications can lead to reductions of up to 24% total weight loss, although it could be less in real-world settings, and longer-term outcomes and tolerability of these drugs are uncertain.^1,3–6^ Although metabolic and bariatric surger is widely undertaken in some countries (>600 000 primary procedures were performed worldwide in 2018), it can be associated with morbidity and there is controversy about which procedure is most clinically effective and cost-effective.^7^ In the UK, Roux-en-Y gastric bypass and adjustable gastric banding predominated until 2015 despite scarce comparative effectiveness data. Only two small randomised controlled trials (RCTs) compared Roux-en-Y gastric bypass with adjustable gastric banding.^8,9^ This context informed the conception of the By-Band study in 2011, which aimed to compare the clinical effectiveness, cost-effectiveness, and safety of Roux-en-Y gastric bypass and adjustable gastric banding. The By-Band study opened in 2012 with the option to consider adding sleeve gastrectomy as a third group. By 2014, sleeve gastrectomy was increasingly used in the UK and internationally, based on safety and short-term outcome data.^10^ At the same time, adjustable gastric banding practice declined because of a perceived lack of effectiveness and the need for re-operation.^7^ In view of this changing practice, with support of the funder and study oversight groups, sleeve gastrectomy was added to By-Band study in 2015. The By-Band-Sleeve trial aimed to compare the clinical effectiveness, cost-effectiveness, and safety of Roux-en-Y gastric bypass, adjustable gastric banding, and sleeve gastrectomy in patients with severe obesity 3 years after randomisation.

## Methods

### Study design and participants

The design and rationale for the addition of a third group and characteristics of the By-Band-Sleeve study participants have been described previously and are summarised in the appendix (p 5)·^11–13^ In brief, the study began as a two-group trial of Roux-en-Y gastric bypass versus adjustable gastric banding. A third group (sleeve gastrectomy) was added after 32 months of recruitment because of changing national and international practice and accumulating data about the safety of sleeve gastrectomy. By-Band-Sleeve study is a pragmatic, multi-centre, open-label, RCT conducted in 12 hospitals in the UK. Adults referred for first-time metabolic and bariatric surgery who met the National Institute for Health and Care Excellence (NICE) guidelines were eligible to participate. Approval was obtained from the Southwest-Frenchay Research Ethics Committee (reference 11/SW/0248) on Dec 6, 2011. All participants gave written informed consent. The trial is registered (ISRCTN No: 00786323, ClinicalTrials.gov NCT02841527).

### Randomisation and blinding

Randomisation took place after baseline assessments had been completed. Participants were initially randomised 1:1 to Roux-en-Y gastric bypass or adjustable gastric banding in six centres. After addition of sleeve gastrectomy, participants were randomised into one of the three groups in 12 sites, varying the allocation ratio by centre to achieve approximate balance in the numbers per group at complete recruitment. Treatment assignments were stratified by centre. Cohort minimisation (with a random element) was used to ensure balance across groups by diabetes status and baseline BMI (appendix p 6). Randomisation was via a secure internet-based system provided by Sealed Envelope (Sealed Envelope, London, UK). Clinicians, research staff, and participants were made aware of the group assignment. Recruitment was supported throughout by a QuinteT Recruitment Intervention that involved interviews with patients, surgeons, and research staff, audio recording of recruitment consultations, analyses of recruitment data and review or revision of patient information. Data were used to support recruiters to explain clinical equipoise and address preferences.^14^

### Procedures

Preoperative evaluation included routine workup, laboratory blood analyses, anthropometric measures, and completion of quality-of-life questionnaires before randomisation. Preoperative endoscopy was performed if clinically indicated. All participants were prescribed a low-calorie diet for 2–4 weeks preoperatively. Thrombo-prophylaxis and pre-operative antibiotics were administered according to national guidelines.

Participating centres were mandated to have a specialist multidisciplinary bariatric team, to perform a minimum of 50 bariatric operations annually and to have a minimum of two surgeons involved in the By-Band-Sleeve trial. Individual surgeons had to have performed at least 50 adjustable gastric banding, 100 Roux-en-Y gastric bypass, and 50 sleeve gastrectomy procedures, be willing to offer participation in the trial to patients, and carry out the surgery according to the randomised allocation and pre-agreed surgical protocols.

Compliance with the allocated surgery was monitored.^15^ A crossover occurred when a participant was allocated one surgery but received another as their primary bariatric surgical procedure. Adherence to the pre-agreed surgical protocols that included mandated, prohibited, and flexible components was recorded. All procedures were to be performed laparoscopically. Roux-en-Y gastric bypass included construction of a small gastric pouch according to the surgeon’s usual practice, except that a horizontal gastric pouch that included fundus was prohibited. The creation of gastrojejunostomy and jejunojejunostomy was at the surgeon’s discretion, although upper limits of 75 cm and 150 cm were recommended for the biliary and gastric limbs respectively. The route of the Roux limb (antecolic or retrocolic) was according to surgeon choice. Closure of iatrogenic mesenteric defects was mandatory from April, 2018, onwards. Using a bougie for the gastric pouch was optional. The type and size of adjustable gastric band used was at the surgeon’s discretion. It was mandatory to (1) dissect the lesser curve using the pars flaccida technique; (2) fix the adjustable gastric band gastro-gastric tunnelling sutures (any fixation method allowed), and (3) fix the adjustment port to the anterior abdominal wall. Sleeve gastrectomy was done by vertical stapled resection of the stomach along the greater curvature, using a bougie up to 40Fr for calibration. Use of additional sutures, clips, reinforcement of the staple line, and its testing was according to surgeon choice. Undertaking a hiatal hernia repair and cholecystectomy were at the surgeon’s discretion. Surgical equipment (eg, type and length of staplers) used was recorded.

### Outcomes

After discharge participants attended hospital for surgical or dietetic, or both, follow-up except during the COVID-19 pandemic, which necessitated telephone appointments. Research appointments were designed to coincide with standard NHS care at 4 weeks post-surgery and month 6, 12, and 24 after randomisation, with one additional follow-up at 3 years after randomisation. Follow-up consultations for participants with an adjustable gastric banding in the first 24 postsurgical months were undertaken according to participant need. Postoperative vitamin and mineral supplementation were prescribed in accordance with national guidelines.

The co-primary outcomes were (1) loss of greater than or equal to 50% excess weight (defined as 100 × [BMI at 3 years – BMI at randomisation]/[BMI at randomisation – 25], and (2) the EQ-5D-5L utility score at 3 years after randomisation. Secondary outcomes included percentage total weight loss and BMI, disease-specific and other generic quality-of-life measures (Short-Form 12 (SF-12), Impact of Weight on Quality of life [IWQOL-Lite], Gastro-intestinal Quality of Life Index [GIQLI], Hospital and Anxiety Depression Scale [HADs]), dietary intake (assessed by interview), and binge eating behaviour (assessed via questionnaire),^13^ sleepiness (Epworth sleepiness scale), and resource use.^14^ Blood measurements were used to assess metabolic control (HbA_1c_, fasting glucose, triglycerides, total cholesterol and HDL-cholesterol, plus blood pressure); safety (haemoglobin, 25-hydroxyvitamin D, calcium, ferritin, folate, parathyroid hormone, serum iron, and vitamin B12); and liver and kidney function (alkaline phosphatase, alanine transaminase, and creatinine). Liver fibrosis was assessed using the ELF test measured at baseline and 3 years.

Adverse health events from randomisation to 3 years were captured and coded using the Medical Dictionary for Regulatory Activities (MedDRA; appendix p 8). Events meeting the regulatory definition of a serious adverse event were identified. Reported abdominal operations and overnight admissions were coded with clinical input. Hospital attendances for abdominal pain were recorded as unexpected serious adverse events.

### Statistical analyses

We hypothesised that Roux-en-Y gastric bypass and sleeve gastrectomy would have non-inferior weight loss and superior quality-of-life to adjustable gastric banding, and that sleeve gastrectomy would have non-inferior weight loss and superior quality-of-life to Roux-en-Y gastric bypass. Both hypotheses had to be supported to conclude that Roux-en-Y gastric bypass or sleeve gastrectomy is more effective than adjustable gastric banding, or that sleeve gastrectomy is more effective than Roux-en-Y gastric bypass. The expected proportion of participants achieving at least 50% excess weight loss at 3 years was 70% (based on registry data). The non-inferiority margin (12%) was chosen by clinicians and patient representatives. The target standardised difference for the EQ-5D-5L was 0·2, with correlations between before and after randomisation measures and repeated after randomisation measures of 0·5 and 0·75, respectively. The sample size of 447 per group was sufficient to test the two hypotheses with 90% power and 1% (one-sided) statistical significance for the non-inferiority hypothesis and 2% (two-sided) statistical significance for the superiority hypothesis, (chosen because there are three comparisons), allowing for 15% loss to follow-up (see appendix p 6).

The statistical analysis plan was finalised before data lock (March 7, 2023) and any analyses were performed. Outcomes were compared on an intention-to-treat (ITT) basis, except where indicated. The ITT, per-protocol, and safety populations are defined in the appendix (pp 8–9). Analyses were adjusted by diabetes status and BMI at baseline and baseline values of the outcome where available fitted as fixed effects. Centre was fitted as a random effect except in cases where the statistical software did not support this approach, in which case robust standard errors clustered by center were used. For longitudinal continuous outcomes, hierarchical mixed models were fitted with participant nested by centre and gradients allowed to vary across time for each participant. Time was modelled with treatment-specific restricted cubic splines. Generalised linear models were used to estimate treatment effects for binary and count outcomes. Pre-specified subgroup analyses (by diabetes status at baseline and BMI category <40 kg/m^2^, 40–50 kg/m^2^ and >50 kg/m^2^) and sensitivity analyses were performed for the primary outcomes (appendix pp 38–41). Analyses of the primary outcomes adjusting for design phase (randomisation to two groups or randomisation to three groups), restricting the analyses to the three-group randomisation phase, and comparing outcomes in the per-protocol population were post hoc. Missing data for the primary weight outcome were imputed using multiple imputation with results combined using Rubin’s rules (appendix pp 9–11). Results are presented as treatment effects with 98% CIs. Analyses were performed using Stata (version 18.0).

Detailed information about the economic evaluations, including a wide range of sensitivity analyses, are described in the appendix (pp 12–17). The primary objective of the economic evaluation was to compare the cost-utility of Roux-en-Y gastric bypass, adjustable gastric banding, and sleeve gastrectomy to 3 years from a UK NHS perspective. The primary analysis was a within-trial analysis performed on an ITT basis, effectively estimating the cost-effectiveness of the decision to perform surgery. Quality-adjusted life-years (QALYs) were estimated using the EQ-5D-5L utility score and expressed in monetary terms by multiplying QALYs gained by the willingness to pay per QALY. Total costs included surgical procedure costs,^15^ and health-care costs related to hospital admissions, outpatient visits, accident and emergency visits, primary care visits, and use of medication and supplements. Costs and outcomes after the first year were discounted at an annual rate of 3·5%. The incremental net monetary benefit was estimated by fitting separate linear regression models for costs and QALYs adjusting for the cohort minimisation variables, treatment allocation, and baseline quality-of-life. Correlation between costs and QALYs was accounted for using non-parametric clustered bootstrapping with a single imputation nested within each bootstrap. The probability of each intervention being the most cost-effective option was also estimated (appendix pp 12–17). In the UK, thresholds of £20 000 and £30 000 per QALY are typically used to support NHS decision making, and the cost-utility at these thresholds was considered. All health economic analyses were performed in R (version 4.0.1).

### Role of the funding source

The funder of the study had no role in study design, data collection, data analysis, data interpretation, or writing of the report.

## Results

**Figure 1:**
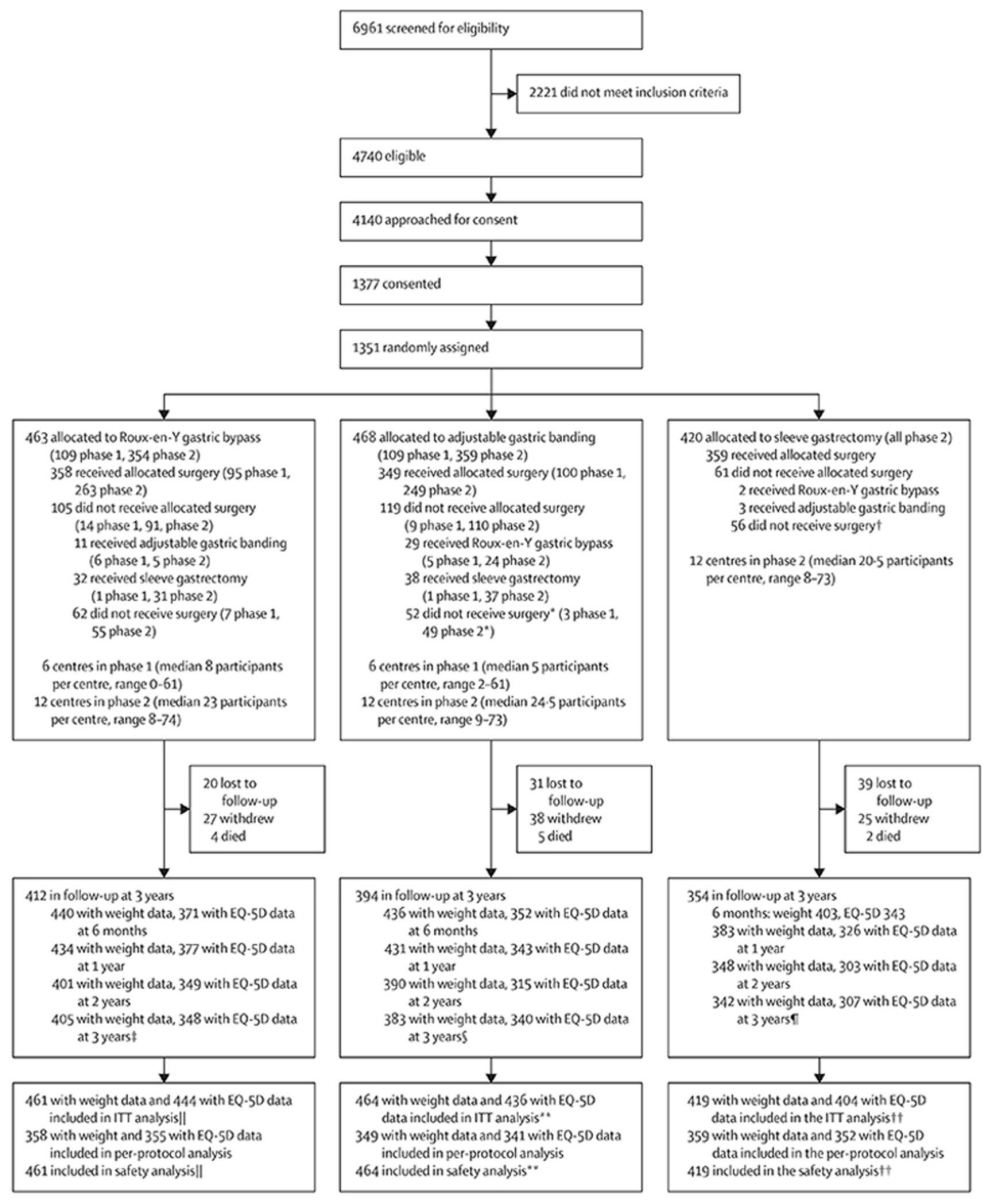
Trial profile Phase 1: two-group randomisation (Roux-en-Y gastric bypass vs adjustable gastric banding), phase 2: three-group randomisation (Roux-en-Y gastric bypass *vs* adjustable gastric banding *vs* sleeve gastrectomy). ITT=intention-to-treat. *†Includes one surgery that was abandoned in theatre. ‡Includes weight data collected from three withdrawals. §Includes weight data collected from four withdrawals. ¶Includes weight data collected from three withdrawals and EQ-5D data collected from two withdrawals. ||One exclusion due to withdrawal of consent for data, one exclusion due to no baseline data. **Four exclusions due to withdrawal of consent for data. ††One exclusion due to no baseline data.

Out of 6961 patients screened, 4140 were eligible and were offered participation. Reasons for ineligibility are given in the appendix (pp 22). Among those who were eligible, 1351 (33%) of 4140 were randomly assigned between Jan 16, 2013, and Sept 27, 2019. Five participants subsequently withdrew consent to use all data. Therefore, 1346 participants were included in this report; 462 were randomly assigned to Roux-en-Y gastric bypass, 464 to adjustable gastric banding, and 420 to sleeve gastrectomy, with 1159 (86%) of 1346 remaining in follow-up at 3 years (figure 1).

The participants’ mean age was 47·3 years (SD 10·6), 1020 (76%) of 1344 were women and 324 (24%) of 1344 were men, mean weight was 129·7 kg (23·6) and mean BMI was 46·4 (6·9) kg/m^2^.

Overall, of 1344 participants, 413 (31%) had diabetes; 92 (7%) self-identified as Black or African or Caribbean or Black British, 46 (3%) as Asian or Asian British, 33 (2%) as mixed or of multiple ethnicity, 33 (2%) as other ethnic group, and 1140 (85%) as White. Baseline characteristics were well balanced across the assigned treatment groups (table 1; appendix pp 23–26).^8^

In total, 163 (12%) of 1346 participants did not undergo surgery within 3 years of randomisation, mostly due to patient choice. Additionally, 115 (10%) of 1183 participants did not receive their randomised allocation with crossovers occurring in 43 (11%) of 401 in the Roux-en-Y gastric bypass group, 67 (16%) of 417 in the adjustable gastric banding group, and five (1%) of 365 participants in the sleeve gastrectomy group. Crossovers to adjustable gastric banding were evenly spread throughout the trial, whereas crossovers to Roux-en-Y gastric bypass or sleeve gastrectomy were more common from 2016 onwards (appendix pp 29–30). Patient choice accounted for 78 (68%) and clinical reasons for 26 (23%) of 115 crossovers, respectively. Characteristics of participants who received the allocated surgery, crossed over or did not undergo surgery within 3 years are summarised in the appendix (pp 31–33). The median time from randomisation to surgery was 5·0 months (IQR 2·5–10·1; appendix p 34). In 1181 participants, surgical adherence to the pre-specified prohibited protocol component was observed in 1181 (100%) and to the mandated component in 1150 (97%). A minority of participants (151 [13%] of 1142) had an additional procedure performed at the time of surgery, of which 114 (10%) of 1142 were hiatal hernia repairs (appendix p 35).

Overall, 307 (85%) of 363 participants in the adjustable gastric banding group had consultations in the first 6 months after surgery and 147 (40%) of 363 had consultations in the third year after surgery. Most participants (833 [86%] of 970) reported taking vitamin and mineral supplements during follow-up.

Overall, of 1346 participants, 1321 (98%) provided data on weight and 1284 (95%) that on EQ-5D-5L at least once during follow-up. Completeness of outcomes measured in blood was lowest for tests that required the participant to fast (eg, fasting glucose). 1254 (93%) participants provided at least one fasting glucose measurement during follow-up (appendix p 36), whereas 144 (11%) failed to complete any secondary quality-of-life questionnaires during follow-up.

**Figure 2:**
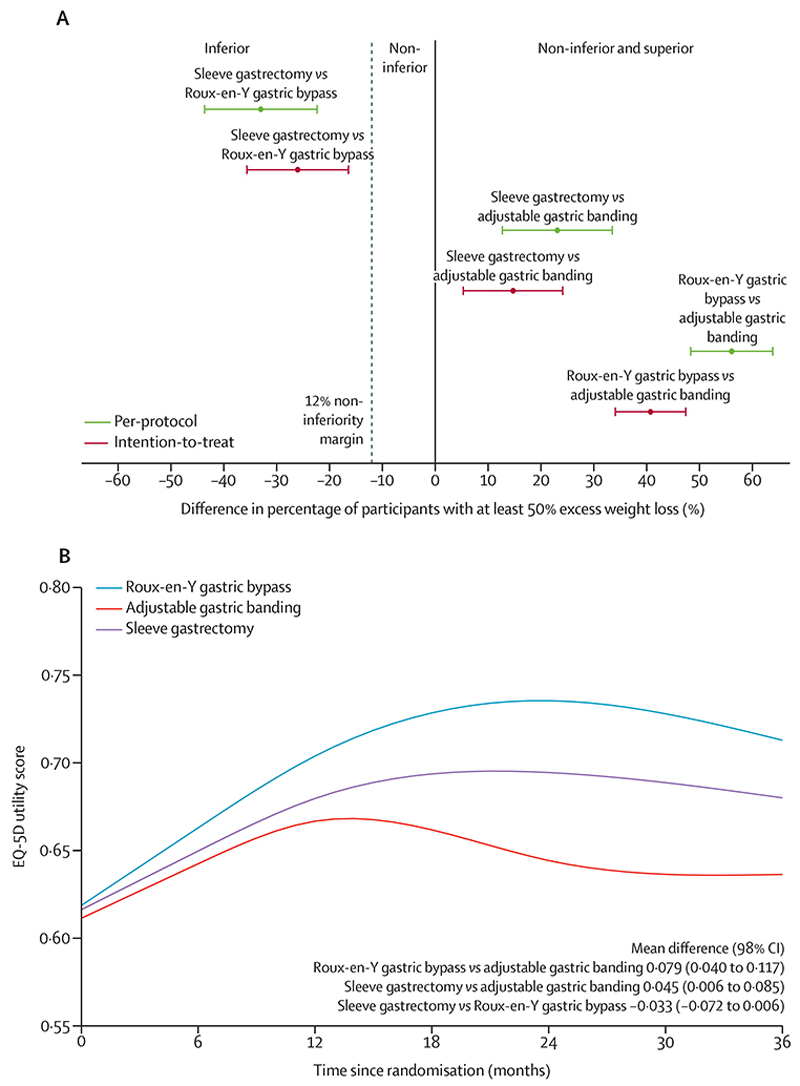
Primary outcomes: results at 3 years after randomisation (all participants) (A) Percentage excess weight loss at 3 years: ITT and per-protocol populations; adjusted risk differences are shown with 98% CIs. (B) EQ-5D utility score to 3 years: ITT population; predicted mean EQ-5D utility scores from randomisation to 3 years and adjusted mean differences in mean EQ-5D utility scores at 3 years from the primary analysis are shown; participants are grouped by allocated surgery. ITT population: all randomly assigned participants excluding those who withdrew consent to use their data; per-protocol population: participants in the ITT population who underwent the allocated surgery within 3 years of randomisation. ITT=intention-to-treat.

In total, 276 (68%) of 405 participants randomised to Roux-en-Y gastric bypass achieved at least 50% excess weight loss at 3 years, compared with 97 (25%) of 383 in the adjustable gastric banding group and 141 (41%) of 342 in the sleeve gastrectomy group. Comparing the risk between groups in relation to the predefined 12% non-inferiority margin, both Roux-en-Y gastric bypass and sleeve gastrectomy were non-inferior (and superior) to adjustable gastric banding, and sleeve gastrectomy was inferior to Roux-en-Y gastric bypass for weight loss (figure 2A). The EQ-5D-5L utility score shows a similar pattern (figure 2B). At 3 years, the mean utility score was significantly higher in the Roux-en-Y gastric bypass group compared with adjustable gastric banding (mean difference 0·079, 98% CI 0·040 to 0·117) and in the sleeve gastrectomy group compared to adjustable gastric banding (0·045, 98% CI 0·006 to 0·085). The difference between sleeve gastrectomy and Roux-en-Y gastric bypass was not significant (–0·033, 98% CI –0·072 to 0·006), although this exceeded the minimally important clinical difference for this measure.^16^

All sensitivity analyses for at least 50% excess weight loss, including the per-protocol analyses limited to the participants who received the allocated surgery, yielded consistent results and subgroup analyses found no statistical evidence to suggest that the treatment effects differed by diabetes status (p=0·90) or weight category at recruitment (p=0·30). Results from all analyses are shown in appendix (pp 38–39) and the weight loss over time for participants in the three randomised groups are shown in appendix (pp 42–43). Sensitivity analyses for EQ-5D-5L utility score were similarly consistent with the primary analysis with an increased difference between sleeve gastrectomy and Roux-en-Y gastric bypass in favour of Roux-en-Y gastric bypass when excluding participants who did not have surgery (appendix pp 40–41). All sensitivity analyses favoured sleeve gastrectomy over adjustable gastric banding, with some being significant at the 2% level. No subgroup differences were found (diabetes p=0·79, baseline BMI p=0·31; appendix pp 40–41). Analyses of outcomes by whether participants were recruited into the two or three group phase of the trial did not alter the findings.

Mean percentage total weight loss at 3 years was –26·8 (SD 13·5) for the Roux-en-Y gastric bypass, –14·0 (13·5) for adjustable gastric banding, and –19·4 (13·1) for the sleeve gastrectomy (table 2). 364 (90%) of 405 participants in the Roux-en-Y gastric bypass group, 217 (57%) of 383 participants in the adjustable gastric banding group, and 269 (79%) of 342 participants in the sleeve gastrectomy group achieved at least 10% total weight loss at 3 years (appendix p 42). HbA^1c^ of less than 48 mmol/mol at 3 years was reached by 293 (91%) of 323 participants in the Roux-en-Y gastric bypass group, 253 (82%) of 308 participants in the adjustable gastric banding group, and 233 (88%) of 266 participants in the sleeve gastrectomy group; of those known to have diabetes at baseline the corresponding percentages were 76% (90 of 118), 50% (51 of 103), and 62% (50 of 81). The reduction in proportion of participants in each group taking anti-diabetic medication followed a similar pattern (appendix p 44). Mean triglycerides were significantly lower for Roux-en-Y gastric bypass compared with adjustable gastric banding (mean difference 0·82, 98% CI 0·76–0·88) and for Roux-en-Y gastric bypass compared with sleeve gastrectomy (1·15, 1·07–1·24) at 3 years. The proportions of participants in each group with a total cholesterol of 5 mmol/L or less at 3 years were 73% (215 of 293) for Roux-en-Y gastric bypass, 58% (166 of 284) for adjustable gastric banding, and 52% (129 of 249) for sleeve gastrectomy. Proportions achieving normotension at 3 years showed a similar pattern (appendix p 45). Full details of secondary outcomes, medication and supplements taken by participants during follow-up are summarised in the appendix (pp 42–64).

The EQ-5D-5L visual analogue scale and the SF-12 physical function scores showed the same improvements as the EQ-5D-5L utility scale over time and between groups. Exceptions were the mental health component score of the SF-12 and the anxiety score of the HADs which showed no differences between groups at 3 years although adjustable gastric banding participants had significantly higher scores (worse depression) than participants receiving Roux-en-Y gastric bypass. The disease-specific quality-of-life measures mostly mirrored the primary outcome (Roux-en-Y gastric bypass and sleeve gastrectomy had better quality-of-life than adjustable gastric banding and differences observed between Roux-en-Y gastric bypass and sleeve gastrectomy favoured Roux-en-Y gastric bypass) with some showing significant differences between groups (eg, the IWQOL-Lite self-esteem scale showed a significant difference between Roux-en-Y gastric bypass and sleeve gastrectomy [mean difference –6·65 points (98% CI –12·3 to –1·00)]; table 2, appendix pp 54–57).

Hospital stay was a median of 2 days and 1099 (93%) of 1181 participants had a post-operative recovery classified as normal (ie, Clavien–Dindo grade 0; appendix p 35). One death occurred during admission for metabolic and bariatric surgery in the adjustable gastric banding group because of peritonitis and sepsis related to a leak from gastric sutures. Rates of all adverse events from surgery to 30 days were similar in the three groups (table 3). There were ten further deaths from randomisation to 3 years, four each in the Roux-en-Y gastric bypass and adjustable gastric banding groups and two in the sleeve gastrectomy group, none of which were attributable to surgery (appendix p 57).

**Figure 3:**
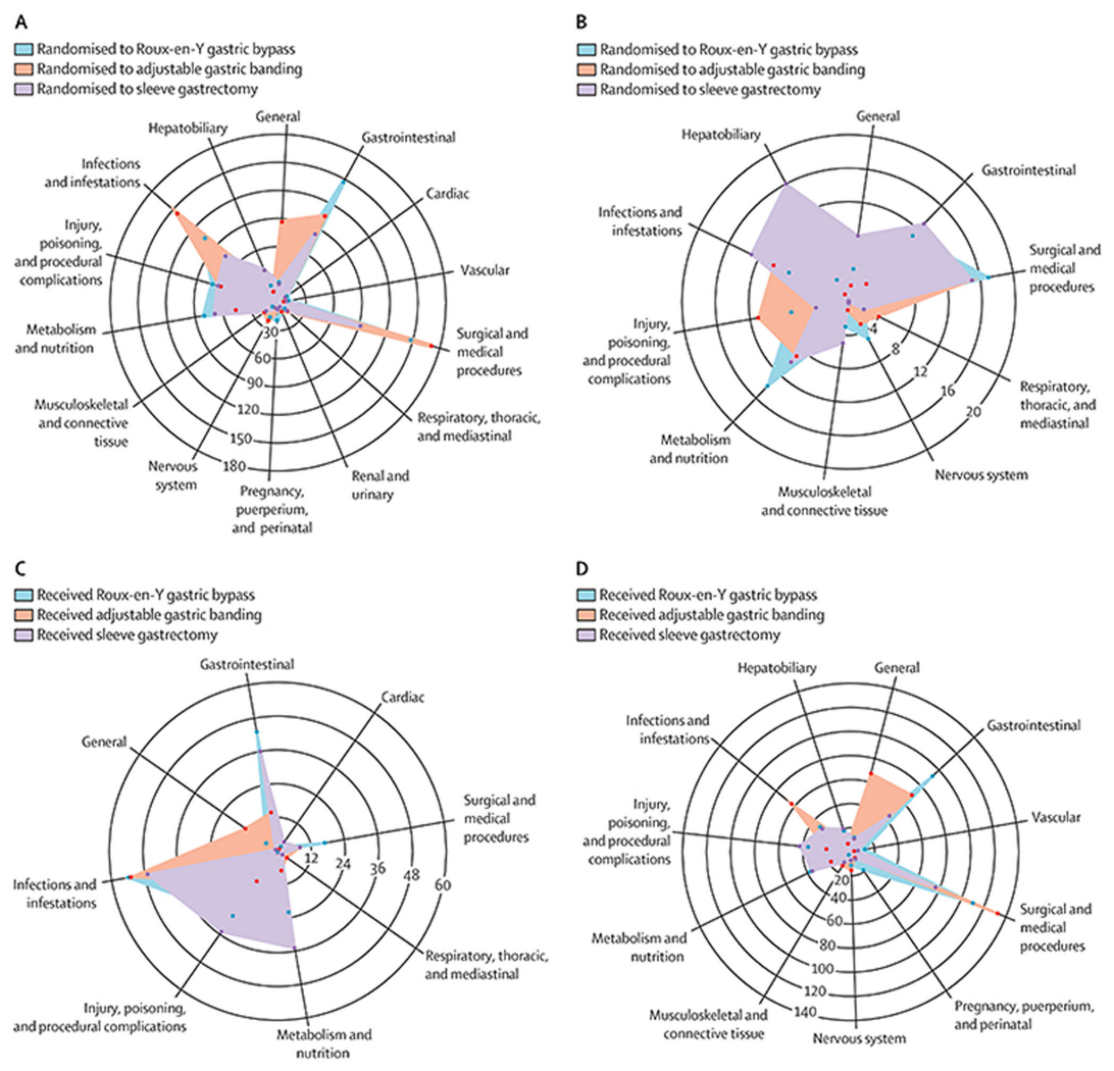
Adverse events reported in trial participants in the 3 years after randomisation (safety populations) (A) Period from randomisation to 3 years: participants grouped by surgery allocated; number of adverse events over the period from randomisation to 3 years within the different MedDRA system organ classes for participants allocated to the three surgeries. (B) Period from randomisation to surgery: participants grouped by surgery allocated; number of adverse events over the period from randomisation to surgery or last follow-up within the different MedDRA system organ classes for participants allocated to the three surgeries. (A, B) Safety population 1: all randomised participants excluding those who withdrew consent to use their data. (C) Period from surgery to 30 days; participants grouped by surgery received; number of adverse events over the period from surgery to 30-days post-surgery within the different MedDRA system organ classes for participants receiving the three surgeries. (D) Period from 30 days post-surgery to 3 years post-randomisation: participants grouped by surgery received; number of adverse events over the period from 30-days post-surgery to 3 years post-randomisation within the different MedDRA system organ classes for participants receiving the three surgeries. (C, D) Safety population 2: all randomised participants who underwent surgery within 3 years of randomisation excluding those who withdrew consent to use their data. The scales on the spider wheels differ across the four panels of the graph.

During the trial 1905 adverse events were reported, of which 242 occurred between randomisation and surgery. The incidence rates per 100 years of follow-up are shown in table 3. Rates of any adverse events were lowest for sleeve gastrectomy, and this was statistically lower compared with the adjustable gastric banding group (incidence rate ratio 0·78, 98% CI 0·62–0·98). The adverse event rate in the period from 30-days post-surgery to 3 years was significantly lower following sleeve gastrectomy compared with Roux-en-Y gastric bypass and adjustable gastric banding surgery (table 3). The proportion of serious adverse events was similar between the sleeve gastrectomy and Roux-en-Y gastric bypass groups (appendix pp 60–64). Complications related to technical aspects of surgery included internal hernia repairs after Roux-en-Y gastric bypass (in 15 [4%] of 389), leaks from the staple line following sleeve gastrectomy (in three [1%] of 429), and revision operations comprising correction, removal, or conversion to another procedure following adjustable gastric banding (in 52 [14%] of 363; appendix p 58). An overview of the adverse events reported by MedDRA system organ class is given in figure 3, with infection predominating in the early post-operative period and the need for further intervention thereafter. Notably, not all events were adverse, because any hospital admission is included even if it is a positive health event such as the birth of a child. The number of positive events was small and balanced across the groups. Although medication usage for reflux increased at 3 years in sleeve gastrectomy patients (appendix p 53), this was not reflected in gastrointestinal quality-of-life scores which were the same for Roux-en-Y gastric bypass and sleeve gastrectomy (table 2; appendix pp 55–56). Six (1%) of 429 participants receiving sleeve gastrectomy developed oesophagitis, 1 (<1%) had a hiatal hernia repair and four (<1%) were subsequently converted to Roux-en-Y gastric bypass (appendix pp 58–59)

The mean costs per participant over the 3 years, including the costs of surgery, were highest for Roux-en-Y gastric bypass and lowest for adjustable gastric banding (appendix p 67). Participants randomised to Roux-en-Y gastric bypass accrued on average more QALYs over the 3 years than those allocated to adjustable gastric banding and sleeve gastrectomy (2·02 [95% CI 1·95–2·09], 1·82 [1·75–1·90], and 1·95 [1·88–2·03], respectively). Combining the costs and QALYs, Roux-en-Y gastric bypass was the most cost-effective option at the cost-utility thresholds applied by NICE, (appendix p 71), with low probabilities that sleeve gastrectomy (<0·30) or adjustable gastric banding (<0·02) are the most cost-effective option (appendix p 71). Results were similar across subgroups and a wide range of sensitivity analyses, including instrumental variable analyses estimating the effect of performing the surgeries at start of follow-up (appendix pp 67–75).

## Discussion

Results from the By-Band-Sleeve study show that for patients with severe obesity referred for metabolic and bariatric surgery according to UK national criteria, Roux-en-Y gastric bypass led to loss of more excess weight, improved quality-of-life, and greater reduction in comorbidities than adjustable gastric banding or sleeve gastrectomy over 3 years. Differences in weight loss after Roux-en-Y gastric bypass and sleeve gastrectomy compared with adjustable gastric banding exceeded the pre-defined non-inferiority margin in both the ITT and post-hoc per-protocol analyses, and quality-of-life was significantly better for Roux-en-Y gastric bypass and sleeve gastrectomy compared with adjustable gastric banding. Weight loss after sleeve gastrectomy was inferior to Roux-en-Y gastric bypass in both analyses and although the difference in quality-of life between Roux-en-Y gastric bypass and sleeve gastrectomy was not significant in the primary ITT analysis, it favoured Roux-en-Y gastric bypass, exceeding the 0·03 threshold for clinical significance.^16^ Sensitivity analyses similarly favoured Roux-en-Y gastric bypass, with some analyses reaching significance. No subgroup differences by diabetes status or weight at baseline were found. Secondary outcomes and disease-specific quality-of-life generally showed similar patterns to those observed in the co-primary outcomes with benefits to Roux-en-Y gastric bypass over sleeve gastrectomy and adjustable gastric banding in some domains although notably mental health scores were similar across the three groups. The economic evaluation strongly supported Roux-en-Y gastric bypass as the most cost-effective option. Focusing on adverse events following surgery, no significant differences were found in the first 30 days after surgery between groups, although in the period from 30 days to 3 years significantly fewer adverse events occurred after sleeve gastrectomy surgery compared with Roux-en-Y gastric bypass and adjustable gastric banding. These findings confirm the prevailing clinical views about adjustable gastric banding that have influenced metabolic and bariatric surgery practice worldwide—ie, adjustable gastric banding is less clinically effective and cost-effective than Roux-en-Y gastric bypass and sleeve gastrectomy. Contrary to dominant clinical views regarding sleeve gastrectomy, this study shows that Roux-en-Y gastric bypass benefits patients and health providers more than sleeve gastrectomy.

Our finding that sleeve gastrectomy is associated with inferior weight loss compared with Roux-en-Y gastric bypass at 3 years distinguishes this trial from previous randomised trials comparing sleeve gastrectomy and Roux-en-Y gastric bypass although our findings are more similar to non-randomised data.^17–21^ The trials favoured Roux-en-Y gastric bypass for weight loss up to 5 years but did not reach prespecified thresholds to draw definitive conclusions. Although the reported trials (and the BEST trial,^22^ which is currently in follow-up) have small differences in patient eligibility criteria (notably fewer people living with diabetes), we do not think that these explain our findings because subgroup and sensitivity analyses all showed similar findings to the main results. Nor do we consider that differences are explained by the quality of surgery or learning curve effects (reasons surgeons commonly disbelieve trial results) because our surgical quality assurance standards before and during the trial were high and reports of adverse events like other studies. Large non-randomised data sets with reported ethnicity details have found a similar weight loss difference between Roux-en-Y gastric bypass and sleeve gastrectomy at 3 years. We therefore consider that it is the size and pragmatic design of By-Band-Sleeve that explains the benefits of Roux-en-Y gastric bypass in terms of weight loss identified. These were not achieved at the expense of increased short-term or serious long-term adverse events (which also confirm the quality of surgery in the By-Band-Sleeve trial).

It is critical to understand rates of adverse events associated with surgical interventions in the context of the clinical benefits. We observed similar rates of short-term adverse events as those reported in the earlier trials and the BEST trial.^17,20–22^ BEST randomised 1735 participants to Roux-en-Y gastric bypass or sleeve gastrectomy. Participants in BEST had a lower mean BMI (41kg/m^2^ *vs* 47 kg/m^2^) and lower proportion of people living with diabetes (12% *vs* 30%) than the By-Band-Sleeve trial. Although we found significantly lower rates of adverse events from 30 days to 3 years after sleeve gastrectomy surgery compared with Roux-en-Y gastric bypass or adjustable gastric banding, it was reassuring the proportion of adverse events classified as serious was similar between the two groups and we did not observe any deleterious effect of Roux-en-Y gastric bypass on nutritional deficiencies or bone mineral complications,^23–25^ or find high rates of hiatal hernia surgery for reflux disease in the sleeve gastrectomy group. Understanding long-term rates of re-operation and serious adverse events is essential and this is planned for By-Band-Sleeve participants.

The benefits of weight loss achieved by Roux-en-Y gastric bypass were mirrored in rates of remission of obesity-related comorbidities (type-2 diabetes, hyper-tension, and dyslipidaemia) which favoured Roux-en-Y gastric bypass compared with sleeve gastrectomy (in keeping with other studies where significant benefits of Roux-en-Y gastric bypass over sleeve gastrectomy were observed for hypertension^17^ and dyslipidaemia^18^). Definitions for remission of obesity-related diseases have changed over the duration of these trials which makes comparisons complicated, but, overall, findings support the effectiveness of Roux-en-Y gastric bypass over sleeve gastrectomy and adjustable gastric banding in terms of metabolic health.^17–19^ The observed mean percentage total weight loss in each group was –26·8 (SD 13·5), –14·0 (13·5), and –19·4 (13·1) for Roux-en-Y gastric bypass, adjustable gastric banding, and sleeve gastrectomy, respectively. Similar weight reductions can be achieved nowadays with pharmacotherapy.^1,3–6^ However, whether the new obesity medications will replace metabolic and bariatric interventions is unknown, and more understanding is needed about the long-term effects and sustainability of these medications compared with surgery.

The addition of sleeve gastrectomy to the By-Band trial to create the By-Band-Sleeve trial, rather than dropping adjustable gastric banding and adding sleeve gastrectomy to create the By-Sleeve trial, in 2015, was controversial. The clinical community had strong views that adjustable gastric banding was ineffective and associated with increased risk compared with other procedures although views were based on two small RCTs and non-randomised data.^8–9,26,27^ The trial oversight groups and patient and public partners in the By-Band-Sleeve trial considered this choice in detail. It was decided to retain adjustable gastric banding because of the scarcity of high-quality evidence. During the trial, every effort was made to support recruiters to provide balanced information to participants based on the published evidence.^28^

Provision of balanced information about treatment options is an essential part of informed consent. It is possible that outside of RCTs, information provision is less balanced during consultations between patients and surgeons especially where innovative surgical procedures are being discussed. Optimism bias for new procedures can result in surgeons not disclosing the uncertainties about risks and effectiveness; patients accepting the new procedure might assume there is supporting evidence of effectiveness.^29^ This might have happened when sleeve gastrectomy was introduced and rapidly adopted worldwide, yet results from the By-Band-Sleeve study (supported by earlier trials) show that prevailing opinion about the benefits of sleeve gastrectomy that led to its widespread use at the expense of Roux-en-Y gastric bypass might have been incorrect. As results from the By-Band-Sleeve study are directly generalisable, the findings challenge clinical practice, although concerns about the longer-term complications of Roux-en-Y gastric bypass and its role in certain patient groups (eg, young women because of nutritional risks) remain. The earlier trials are now reporting long-term data and such concerns have not yet emerged.

The main strengths of the By-Band-Sleeve study are the design and conduct which allowed it to recruit successfully, made it pragmatic beyond the UK, and protected it from bias.^9–10^ A third of participants had type 2 diabetes and a representative proportion of participants came from ethnic minority groups. The Black ethnic group is well represented at 7% compared with 4% of the UK population although the proportion of people with Asian heritage (3%) was smaller than in the UK population (9%). To our knowledge, this is the first RCT in metabolic and bariatric surgery that has reported ethnicity. 12 specialist surgical teams from a diverse set of hospitals across the UK including over 40 surgeons participated. All met study entry criteria for volume and quality of surgery, and surgical interventions were delivered according to the protocol with high rates of adherence to pre-agreed mandated surgical standards. Study conduct was supported with annual investigator meetings providing feedback on recruitment practice (based on qualitative analyses of audio recordings of consultations) and centre data on adherence to surgical protocols including compliance with the allocated surgery. A main strength is that the study included a comprehensive set of patient-reported outcome measures and a cost-effectiveness analysis. Although several previous cost-effectiveness analyses indicate that bariatric surgery is likely to be cost-effective compared with non-surgical interventions, our results provide more certainty and precision about the comparative cost-effectiveness of these three procedures.^30–32^

The study has limitations. Since the trial was designed, the percentage total weight loss has become more commonly used as a primary outcome, and international recommendations have been made about trial outcome selection and reporting. We report percentage total weight loss as a secondary outcome to allow synthesis with pharmaceutical studies and include all the outcomes recommended in the core outcome set for bariatric surgery.^33^ Another limitation is that baseline measurements were taken on average 5 months before surgery, and some participants did not have surgery. It was difficult to randomise close to the time of surgery because of the need to prepare participants and operating lists, although randomising the day before surgery would have been preferable. Analyses performed accounted for clustering at the centre level. We did not account for clustering by surgeon because evidence from previous surgical trials (including those where the randomisation was expertise-based) suggested the interclass correlation at the surgeon level is negligible; metabolic and bariatric surgery is also a complex intervention, and the surgical after care is critical. Trial allocation and surgery received were not blinded to participants or personnel because adjustable gastric banding required different post-operative care schedules. It is possible that participants could have been blinded to Roux-en-Y gastric bypass and sleeve gastrectomy and that might have enhanced the differences observed due to surgeons’ and patients’ beliefs about the superior benefits of sleeve gastrectomy. Since the initiation of the trial, national guidance for metabolic and bariatric surgery for people of Asian heritage, Middle Eastern, and Black African or African-Caribbean family background has changed to recommend surgical consideration at a lower BMI than other populations. Although our inclusion criteria did not change in line with guidance, it will be possible to explore the treatment effects in these ethnic groups. The use of the EQ-5D-5L as a co-primary endpoint which asks participants to describe their current health status, might risk capturing an untypical short-term health fluctuation although long lasting effects of surgery are not expected to change markedly in a 24-h period and similar fluctuations probably occurred in each group. However, this well-validated and easy-to-use instrument has many strengths. It is recommended for use in future trials of metabolic and bariatric surgery to capture the broader impact of surgery on health and allow comparisons with other interventions. An additional limitation is the 6 years needed to complete trial recruitment. Although this prolonged timeline has delayed the release of results, without this level of commitment, essential comparative data would remain unavailable. This study illustrates that, with sufficient investment and training, effective recruitment into challenging surgical trials is achievable.

Although every effort was made to conduct the trial to the highest standard, and 1159 participants (ie, 86%) remained in follow-up at 3 years, the data completeness for some secondary outcomes was low. This limits validity of the results, although there was no evidence to suggest the data were missing differentially by group. As some of the follow-up occurred during the pandemic this is likely to have impacted on participant retention, however, follow-up rates are comparable to those reported in the Biter trial.^17^ Another limitation is that follow-up consultations for adjustable gastric banding were fewer than intended in the protocol. A stricter post-operative adjustable gastric banding protocol might have resulted in better outcomes in this group although it is considered unlikely that they would be comparable with sleeve gastrectomy or Roux-en-Y gastric bypass. The observed weight loss in the adjustable gastric banding group was comparable to results from centres specialising in adjustable gastric banding care.^26,34^

The results of this pragmatic RCT confirm the safety and effectiveness of metabolic and bariatric surgery and that Roux-en-Y gastric bypass and sleeve gastrectomy are more effective than adjustable gastric banding. Sleeve gastrectomy had inferior weight loss compared with Roux-en-Y gastric bypass, and sleeve gastrectomy was less clinically effective for quality-of-life compared with Roux-en-Y gastric bypass. Future research to understand longer term outcomes and to compare metabolic and bariatric surgery with obesity management medications is needed to guide evidence-based practice in this rapidly evolving field.

## Supplementary Material

Supplementary material

## Figures and Tables

**Table 1:** Baseline characteristics

	Randomised to Roux-en-Y gastric bypass (n=462)	Randomised to adjustable gastric banding (n=464)	Randomised to sleeve gastrectomy (n=420)	Overall (n=1346)
Age, years*	47·4 (10·3)	46·8 (10·4)	47·4 (11·0)	47·4 (10·4)
Sex				
Male	116/461 (25%)	110/464 (24%)	98/419 (23%)	324/1344 (24%)
Female	345/461 (75%)	354/464 (76%)	321/419 (77%)	1020/1344 (76%)
Ethnicity				
White	401/461 (87%)	394/464 (85%)	345/419 (82%)	1140/1344 (85%)
Mixed or multiple ethnic groups	11/461 (2%)	11/464 (2%)	11/419 (3%)	33/1344 (2%)
Asian or Asian British	10/461 (2%)	21/464 (5%)	15/419 (4%)	46/1344 (3%)
Black or African or Caribbean or Black British	30/461 (7%)	26/464 (6%)	36/419 (9%)	92/1344 (7%)
Other ethnic group	9/461 (2%)	12/464 (3%)	12/419 (3%)	33/1344 (2%)
Weight, kg*	131·4 (23·8)	129·0 (23·1)	128·7 (23·8)	129·7 (23·6)
BMI, kg/m^2^*	46·9 (7·1)	46·1 (6·6)	46·1 (6·9)	46·4 (6·9)
Waist circumference, cm†	130·0 (120·3 –140·4)	129·5 (120·4–139·3)	128·2 (118·5–139·8)	129·3 (120·0–139·9)
Diabetes	152/461 (33%)	144/464 (31%)	117/419 (28%)	413/1344 (31%)
Duration of diabetes, years‡	3·6 (1·8–9·0)	5·0 (2·4–9·8)	4·8 (2·2–10·3)	4·7 (2·1–9·8)
HbA_1c_, mmol/mol	53 (44–64)	52 (44–64)	51 (44–63)	52 (44–64)
HbA_1c_, %	7·0 (6·2–8·0)	6·9 (6·2–8·0)	6·8 (6·2–7·9)	6·9 (6·2–8·0)
Fasting glucose, mmol/L	6·6 (6–8)	6·5 (6–9)	6·9 (5–9)	6·7 (6–9)
No diabetes	309/461 (67%)	320/464 (69%)	302/419 (72%)	931/1344 (69%)
HbA_1c_, mmol/mol	37 (35–40)	38 (35–41)	38 (35–41)	38 (35–41)
HbA_1c_, %	5·5 (5·4–5·8)	5·6 (5·4–5·9)	5·6 (5·4–5·9)	5·6 (5·4–5·9)
Fasting glucose, mmol/L	5·0 (5–5)	5·0 (5–5)	5·0 (5–5)	5·0 (5–5)
All participants				
HbA_1c_, mmol/mol§	40 (36–47)	40 (36–-45)	39 (36–45)	40 (36–45)
HbA_1c_,%§	5·8 (5·4–6·5)	5·8 (5·4–6·3)	5·7 (5·4–6·3)	5·8 (5·4–6·3)
Fasting glucose, mmol/L¶	5·2 (4·7–6·2)	5·2 (4·7–5·8)	5·1 (4·7–5·9)	5·2 (4·7–5·9)
Anti-diabetic medication	137/461 (30%)	125/464 (27%)	103/416 (25%)	365/1344 (27%)
Smoker	43/460 (9%)	39/464 (8%)	41/419 (10%)	123/1343 (9%)
Obstructive sleep apnoea	127/460 (28%)	135/464 (29%)	94/418 (22%)	356/1342 (27%)
Gastro-oesophageal reflux disease or hiatus hernia	209/460 (45%)	229/464 (49%)	212/419 (51%)	650/1343 (48%)
Back or leg pain from arthritis	251/460 (55%)	247/464 (53%)	226/419 (54%)	724/1343 (54%)
Anti-hypertensive medication	210/462 (45%)	178/464 (38%)	176/420 (42%)	564/1346 (42%)
Anti-hyperlipidaemia medication	131/424 (31%)	127/429 (30%)	110/379 (29%)	368/1232 (30%)
Systolic blood pressure, mm Hg||	132·6 (121·5–144·5)	132·0 (121·0–145·0)	134·2 (122·5–145·8)	133·0 (121·5–145·0)
Diastolic blood pressure, mm Hg||	83·5 (76·0–90·0)	82·0 (76·0–88·3)	82·4 (75·4–89·4)	82·5 (76·0–89·0)
Total cholesterol, mmol/L§	4·8 (1·2)	4·8 (1·0)	4·9 (1·0)	4·8 (1·1)
HDL-cholesterol, mmol/L**	1·2 (1·0–1·4)	1·2 (1·1–1·4)	1·2 (1·1–1·5)	1·2 (1·0–1·4)
Triglycerides, mmol/L††	1·4 (1·1–2·0)	1·4 (1·1–1·9)	1·4 (1·1–1·9)	1·4 (1·1–1·9)
EQ-5D-5L utility score‡‡	0·61 (0·29)	0·60 (0·28)	0·61 (0·28)	0·61 (0·28)

Data are median (IQR), mean (SD), or n/N (%).

* Data were missing for one participant each in the Roux-en-Y gastric bypass and the sleeve gastrectomy groups.

† Data were missing for four participants in the Roux-en-Y gastric bypass group and six participants each in the adjustable gastric banding and the sleeve gastrectomy groups.

‡ Data were missing for two participants in the Roux-en-Y gastric bypass group and four participants in the sleeve gastrectomy group.

§ Data were missing for ten participants in the Roux-en-Y gastric bypass group and nine participants each in the adjustable gastric banding and the sleeve gastrectomy groups.

¶ Data were missing for 39 participants in the Roux-en-Y gastric bypass group, 22 participants in the adjustable gastric banding group, and 18 participants in the sleeve gastrectomy group.

|| Data were missing for two participants in the Roux-en-Y gastric bypass group, 11 participants in the adjustable gastric banding group, and four participants in the sleeve gastrectomy group.

** Data were missing for 11 participants each in the Roux-en-Y gastric bypass and the adjustable gastric banding groups and ten participants in the sleeve gastrectomy group.

†† Data were missing for 19 participants in the Roux-en-Y gastric bypass group, 16 participants in the adjustable gastric banding group, and nine participants in the sleeve gastrectomy group.

‡‡ Data were missing for one participant in the Roux-en-Y gastric bypass group, ten participants in the adjustable gastric banding group, and four participants in the sleeve gastrectomy group.

**Table 2:** Key secondary outcomes: results at 3 years after randomisation

	Randomised to Roux-en-Y gastric bypass (mean [SD] or geometric mean [CV])	Randomised to adjustable gastric banding (mean [SD] or geometric mean [CV])	Randomised to sleeve gastrectomy (mean [SD] or geometric mean [CV])	Roux-en-Y gastric bypass *vs* adjustable gastric banding (MD [98% CI] or GMR [98% CI])	Sleeve gastrectomy *vs* adjustable gastric banding (MD [98% CI] or GMR [98% CI])	Sleeve gastrectomy *vs* Roux-en-Y gastric bypass (MD [98% CI] or GMR [98% CI])
**Weight outcomes**						
Weight, kg						
Baseline	131·4 (23·8)	129·0 (23·1)*	128·7 (23·8)*	··	··	··
3 years	95·4 (24·3)*	110·9 (26·0)*	102·8 (23·7)*	··	··	··
Percentage total weight loss	–26·8 (13·5)*	–14·0 (13·5)*	–19·4 (13·1)*	–12·7 (–14·9 to –10·4)†	–5·82 (–8·12 to –3·52)†	6·83 (4·55 to 9·11)†
BMI, kg/m^2^						
Baseline	46·9 (7·1)*	46·1 (6·6)*	46·1 (6·9)*	··	··	··
3 years	34·0 (7·6)*	39·6 (8·2)*	37·0 (7·6)*	–5·93 (–7·02 to –4·85)†	–2·75 (–3·87 to –1·64)†	3·18 (2·07 to 4·29)†
**Quality of life outcomes**						
EQ-5D visual analogue scale						
Baseline	61·6 (21·0)*	61·6 (19·9)*	61·3 (22·0)*	··	··	··
3 years	75·1 (19·6)*	67·9 (22·5)*	72·8 (20·6)*	7·79 (4·73 to 10·84)†	5·71 (2·58 to 8·35)†	–2·07 (–5·18 to 1·03)†
SF-12 physical component score						
Baseline	39·0 (10·6)*	38·4 (10·5)*	39·0 (10·9)*	··	··	··
3 years	48·8 (11·2)*	44·2 (12·3)*	46·9 (11·4)*	3·87 (1·87to 5·87)†	2·54 (0·49 to 4·59)†	–1·33 (–3·35 to 0·70)†
SF-12 mental component score						
Baseline	43·1 (11·1)*	42·7 (11·3)*	43·5 (10·9)*	··	··	··
3 years	46·7 (10·7)*	45·6 (12·3)*	45·9 (12·3)*	1·66 (–0·44 to 3·76)†	0·18 (–1·98 to 2·34)†	–1·48 (–3·62 to 0·65)†
HADS—anxiety						
Baseline	7·6 (4·5)*	8·0 (4·4)*	7·5 (4·2)*	··	··	··
3 years	6·38 (4·6)*	6·91 (5·1)*	6·71 (5·1)*	–0·66 (–1·48 to 0·15)†	–0·20 (–1·04 to 0·64)†	0·47 (–0·36 to 1·30)†
HADS—depression						
Baseline	7·8 (4·3)*	7·6 (3·9)*	7·3 (4·0)*	··	··	··
3 years	4·36 (4·6)*	5·47 (5·1)*	4·73 (4·8)*	–1·58 (–2·43 to –0·72)t	–0·78 (–1·66 to 0·10)t	0·80 (–0·07 to 1·67)*
GIQLI overall						
Baseline	86·5 (17·8)*	84·7 (16·2)*	86·0 (16·3)*	··	··	··
3 years	99·3 (17·9)*	92·8 (18·0)*	97·2 (17·1)*	5·70 (2·53 to 8·87)†	4·41 (1·14 to 7·67)†	–1·29 (–4·51 to 1·93)†
GIQLI gastrointestinal symptoms						
Baseline	58·1 (9·9)*	57·2 (9·3)*	57·7 (9·6)*	··	··	··
3 years	58·6 (9·9)*	56·3 (9·3)*	58·1 (9·6)*	2·10 (0·39 to 3·82)†	1·96 (0·19 to 3·72)†	–0·15 (–1·89 to 1·60)†
IWQOL overall						
Baseline	42·1 (22·0)*	41·2 (21·0)*	42·2 (20·3)*	··	··	··
3 years	78·6 (22·0)*	67·1 (25·4)*	74·4 (22·2)*	11·4 (638 to 15·8)†	7·60 (3·04 to 12·2)†	–3·81 (–8·32 to 0·71)†
IWQOL self-esteem						
Baseline	31·9 (26·8)*	30·2 (26·1)*	31·4 (26·2)*	··	··	··
3 years	72·2 (28·5)*	57·8 (31·1)*	65·0 (30·4)*	14·0 (8·43 to 19·6)†	7·35 (1·63 to 13·1)†	–6·65 (–12·3 to –1·00)†
IWQOL sexual life						
Baseline	44·0 (32·4)*	43·5 (31·1)*	42·6 (31·9)*	··	··	··
3 years	70·4 (33·4)*	64·9 (33·0)*	69·1 (33·6)*	8·07 (1·88 to 14·26)†	8·2 (1·80to 14·61)†	0·13 (–6·16 to 6·43)†
IWQOL public distress						
Baseline	41·6 (27·2)*	41·0 (26·9)*	44·3 (26·3)*	··	··	··
3 years	82·6 (23·2)*	71·5 (28·5)*	80·0 (24·0)*	11·83 (7·00 to 16·66)†	9·00 (4·02 to 13·97)†	–2·83 (–7·75 to 2·08)†
**Metabolic control**						
HbA_1c_, mmol/mol						
Baseline	42·3 (0·3)‡	42·3 (0·3)‡	41·4 (0·3)‡	··	··	··
3 years	37·9 (0·2)‡	40·4 (0·3)‡	38·3 (0·3)‡	0·94 (0·91 to 0·97)§	0·96 (0·93 to 0·99)§	1·02 (0·99 to 1·06)§
HbA_1c_, %						
Baseline	6·1 (0·2)‡	6·1 (0·2)‡	6·0 (0·2)‡	··	··	··
3 years	5·6 (0·2)‡	5·9 (0·3)‡	5·6 (0·3)‡	··	··	··
Fasting glucose, mmol/L						
Baseline	5·5 (0·3)‡	5·5 (0·3)‡	5·5 (0·3)‡	··	··	··
3 years	5·1 (0·2)	5·3 (0·3)‡	5 (0·3)‡	0·94 (0·89 to 0·98)§	0·95 (0·91 to 1·00)§	1·02 (0·97 to 1·06)§
Triglycerides, mmol/L						
Baseline	1·5 (0·5)‡	1·4 (0·5)‡	1·4 (0·5)‡	··	··	··
3 years	1·1 (0·4)‡	1·3 (0·5)‡	1·2 (0·5)‡	0·82 (0·76 to 0·88)§	0·94 (0·87 to 1·01)§	1·15 (1·07to 1·24)§
HDL cholesterol, mmol/L						
Baseline	1·2 (0·3)‡	1·2 (0·2)‡	1–2 (03)*	··	··	··
3 years	1·6 (0·3)‡	1·4 (0·3)‡	1·5 (0·3)‡	1·14 (1·09 to 1·19)§	1·09 (1·04 to 1·13)§	0·96 (0·92 to 1·00)§
Total cholesterol, mmol/L						
Baseline	4·8 (1·2)*	4·8 (1·0)*	4·9 (1·0)*	··	··	··
3 years	4·4 (1·0)*	4·8 (1·1)*	5·0 (1·0)*	–0·31 (–0·46 to –0·15)†	0·08 (–0·08 to 0·24)†	0·39 (0·22 to 0·55)†
**Safety bloods**						
Ferritin, μg/L						
Baseline	56·5 (1·1)‡	61·6 (1·1)‡	65 (1·2)‡	··	··	··
3 years	49·2 (1·7)‡	56·8 (1·4)‡	64·1 (1·5)‡	1·95 (0·81 to 1·11)§	1·33 (0·96 to 1·33)§	1·19 (1·02 to 1·40)§
Serum iron, μmol/L						
Baseline	12·7 (0·4)‡	12·4 (0·5)‡	12·6 (0·4)‡	··	··	··
3 years	15·0 (0·5)‡	14·4 (0·4)‡	15·4 (0·5)‡	1·05 (0·96 to 1·14)§	1·09 (1·00to 1·19)§	1·04 (0·95 to 1·13)§
Folate, μg/L						
Baseline	6·6 (0·6)‡	6·2 (0·6)‡	6·4 (0·6)‡	··	··	··
3 years	10·8 (0·8)‡	8·7 (0·8)‡	9·6(0·8)‡	1·23 (1·09 to 1·40)§	1·11 (0·97 to 1·26)§	0·90 (0·79 to 1·02)§
Vitamin B12, ng/L						
Baseline	315·2 (0·5)‡	307 (0·5)‡	323 (0·5)#x2021;	··	··	··
3 years	519·7 (0·8)‡	374·3 (0·6)‡	592·9 (0·8)‡	1·36 (1·22 to 1·52)§	1·46 (1·30 to 1·64)§	1·07 (0·95 to 1·20)§
25 hydroxyvitamin D, nmol/L						
Baseline	39·9 (0·6)‡	41·5 (0·6)‡	40·6 (0·6)‡	··	··	··
3 years	60·6 (0·5)‡	52·2 (0·5)‡	61·6 (0·6)‡	1·16 (1·06 to 1·27) ·	115 (1·05 to 1·27)§	0·99 (0·91 to 1·09)§
Parathyroid hormone, pmol/L						
Baseline	5·9 (0·5)‡	5·7 (0·5)‡	5·8 (0·5)‡	··	··	··
3 years	5·9 (0·6)‡	5·8 (0·5)‡	5·9(0·6)‡	1·04 (0·95 to 1·13)§	0·98 (0·90 to 1·07)§	0·95 (0·87 to 1·03)§
Haemoglobin, g/dL						
Baseline	13·9 (1·36)*	13·8 (1·27)*	13·8 (1·26)*	··	··	··
3 years	13·4 (1·34)*	13·6 (1·32)*	13·5 (1·32)*	–0·31 (–0·49 to –0·13)†	–0·15 (–0·34 to 0·04)†	0·16 (–0·03 to 0·35)†
Calcium, mmol/L						
Baseline	2·35 (0·10)*	2·37 (0·10)*	2·36 (0·09)*	··	··	··
3 years	2·32 (0·10)*	2·36 (0·11)*	2·35 (0·11)*	–0·027 (–0·045 to –0·010)†	–0·003 (–0·021 to 0·015)†	0·024 (0·007 to 0·042)†
**Liver and kidney function**						
ALT, IU/L						
Baseline	24·2 (0·5)‡	24·9 (0·5)‡	24·4 (0·5)‡	··	··	··
3 years	21·3 (0·5)‡	19·3 (0·5)‡	17·9 (0·5)‡	1·09 (1·00 to 1·18)§	0·95 (0·87 to 1·03)§	0·87 (0·80 to 0·95)§
ALP, IU/L						
Baseline	81·3 (0·3)‡	81·7 (0·3)‡	81·9 (0·3)‡	··	··	··
3 years	83·8 (0·3)‡	79 (0·3)‡	75·3 (0·4)‡	1·07 (1·03 to 1·12)§	0·96 (0·91 to 1·00)§	0·89 (0·85 to 0·93)§
Creatinine, pmol/L						
Baseline	66·7 (0·2)‡	66·7 (0·2)‡	66·1 (0·2)‡	··	··	··
3 years	64·1 (0·3)‡	66·0 (0·2)‡	65·4 (0·3)‡	0·98 (0·95 to 1·00)§	1·00 (0·98 to 1·03)§	1·03 (1·00 to 1·05)§

ALP=alkaline phosphatase. ALT=alanine transaminase. CV=coefficient of variation. GIQOL=gastrointestinal quality of life. GMR=geometric mean ratio (Roux-en-Y gastric bypass to adjustable gastric banding, sleeve gastrectomy to adjustable gastric banding, and sleeve gastrectomy to Roux-en-Y gastric bypass). HADS=hospital anxiety and depression scale. IWQOL=impact of weight on quality of life. MD=mean difference (Roux-en-Y gastric bypass minus adjustable gastric banding, sleeve gastrectomy minus adjustable gastric banding, and sleeve gastrectomy minus Roux-en-Y gastric bypass). SF-12=short-form 12. Data are:

* mean (SD)

† MD (98% CI)

‡ geometric mean (CV)

§ GMR (98% CI).

**Table 3:** Rates of adverse events: results at 3 years after randomisation by allocation and by surgery received

	Roux-en-Y gastric bypass (events [rate])	Adjustable gastric banding (events [rate])	Sleeve gastrectomy (events [rate])	Roux-en-Y gastric bypass *vs* adjustable gastric banding (IRR [98% CI])	Sleeve gastrectomy *vs* adjustable gastric banding (IRR [98% CI])	Sleeve gastrectomy *vs* Roux-en-Y gastric bypass (IRR [98% CI])
**Any adverse event by randomised allocation**						
Randomisation to 3 years, rate per 100 years	675 (56·8)	705 (62·0)	525 (50·2)	0·89 (0·72–1·12)	0·78 (0·62–0·98)	0·87 (0·69–1·10)
Surgery to 30 days post-surgery, rate per 100 days	183 (1·53)	155 (1·26)	140 (1·30)	1·20 (0·85–1·68)	1·01 (0·71–1·45)	0·84 (0·59–1·20)
30 days post-surgery to 3 years, rate per 100 years	410 (48·8)	485 (57·5)	290 (40·7)	0·81 (0·61–1·07)	0·65 (0·49–0·88)	0·81 (0·60–1·10)
Post-surgery abdominal procedure*						
Surgery to 3 years, rate per 100 years	47 (5·39)	79 (9·01)	29 (3·91)	0·56 (0·31–1·01)	0·41 (0·21–0·78)	0·72 (0·36–1·44)
Hospital attendance for abdominal pain						
Randomisation to 3 years, rate per 100 years	38 (3·20)	37 (3·25)	31 (2·96)	1·00 (0·51–1·97)	0·90 (0·44–1·83)	0·90 (0·45–1·82)
Surgery to 3 years, rate per 100 years	35 (4·01)	37 (4·22)	24 (3·24)	0·96 (0·47–1·96)	0·77 (0·35–1·66)	0·80 (0·37–1·73)
Overnight admission for any reason						
Randomisation to 3 years, rate per 100 years	179 (15·1)	189 (16·6)	161 (15·4)	0·90 (0·66–1·21)	0·89 (0·65–1·21)	0·99 (0·73–1·35)
Surgery to 3 years, rate per 100 years	153 (17·5)	164 (18·7)	127 (17·1)	0·90 (0·66–1·25)	0-85 (0·61–1·20)	0·94 (0·67–1·33)
**Any adverse event by received surgery**						
Surgery to 30 days post-surgery, rate per 100 days†	175 (1·51)	120 (1·11)	181 (1·44)	1·38 (0·96–1·98)	1·27 (0·89–1·83)	0·92 (0·66–1·29)
30 days post-surgery to 3 years, rate per 100 years†	412 (51·4)	443 (57·1)	320 (39·3)	0·85 (0·64–1·14)	0·63 (0·47–0·85)	0·74 (0·55–0·99)
Post-surgery abdominal procedure*						
Surgery to 3 years, rate per 100 years†	52 (6·24)	74 (9·19)	29 (3·41)	0·64 (0·36–1·15)	0·35 (0·18–0·67)	0·54 (0·28–1·06)
Hospital attendance for abdominal pain						
Surgery to 3 years, rate per 100 years†	38 (4·56)	31 (3·85)	27 (3·18)	1·22 (0·59–2·55)	0·81 (0·37–1·74)	0·66 (0·31–1·39)
Overnight admission for any reason						
Surgery to 3 years, rate per 100 years†	157 (18·8)	142 (17·6)	143 (16·8)	1·03 (0·74–1·44)	0·90 (0·64–1·26)	0·87 (0·63–1·21)

IRR=incidence rate ratio (Roux-en-Y gastric bypass to adjustable gastric banding, sleeve gastrectomy to adjustable gastric banding, and sleeve gastrectomy to Roux-en-Y gastric bypass).

* Abdominal procedures includes appendicectomy, caesarean section, cholecystectomy, hernia repair (abdominal, hiatus, incisional, umbilical, unspecified), hysterectomy, jejunostomy, laparoscopy, laparotomy, oophorectomy (unilateral, bilateral, and unspecified), hysterosalpingo-oophorectomy, salpingectomy, salpingo-oophorectomy (bilateral and unilateral), peritoneal adhesions division, small intestinal resection, splenectomy, liposuction, gastrostomy, wound drainage, enteral nutrition, abdominoplasty, adhesiolysis, peritoneal lavage, abdominal cavity drainage, gastric banding, gastric bypass, gastric operation, gastrointestinal surgery, gastric banding reversal, drain placement, stoma closure, stoma creation, and intestinal plication surgery.

† Two surgeries (one adjustable gastric banding and one sleeve gastrectomy) were attempted but abandoned (adjustable gastric banding because participant deteriorated anaesthetically; sleeve gastrectomy because participant became hypotensive 10 min after the laparoscopy with a possible cardiac event), these two surgeries are included in the analyses by randomised allocation but are excluded from the analyses by surgery received.

## Research in context

### Evidence before this study

Metabolic bariatric surgery is a well established intervention for people living with severe obesity, yet the optimal surgical procedure remains uncertain. Before 2012, Roux-en-Y gastric bypass and adjustable gastric banding were the most commonly performed procedures; by 2015, this had changed to Roux-en-Y gastric bypass and sleeve gastrectomy. However, the comparative evidence for all procedures was limited and less was known about the impact of different types of metabolic and bariatric surgery on quality-of-life. Reviews highlighted that trials would be difficult to conduct because preferences might prohibit randomisation.

### Added value of this study

The By-Band-Sleeve study randomised 1351 participants with support of a recruitment intervention. 88% of participants underwent surgery. At 3 years follow-up, Roux-en-Y gastric bypass and sleeve gastrectomy were more effective than adjustable gastric banding, with non-inferior weight loss and superior quality-of life. Weight loss following sleeve gastrectomy was inferior to Roux-en-Y gastric bypass. Quality-of-life differences between sleeve gastrectomy and Roux-en-Y gastric bypass were not significant, but favoured Roux-en-Y gastric bypass. Fewer adverse events occurred after sleeve gastrectomy compared with Roux-en-Y gastric bypass and adjustable gastric banding but Roux-en-Y gastric bypass was found to be the most cost-effective and to provide value for money for the National Health Service. Subgroup analyses by diabetes status and weight at baseline supported the findings of the primary analyses.

### Implications for all the available evidence

Roux-en-Y gastric bypass and sleeve gastrectomy are more effective than adjustable gastric banding for weight loss, quality-of-life, and reduction in comorbidities at 3 years follow-up. Sleeve gastrectomy is inferior to Roux-en-Y gastric bypass for weight loss with lower but statistically similar quality of life. Trials comparing Roux-en-Y gastric bypass with obesity management medications will inform the management of the severe obesity as the prevalence continues to rise.

## Data sharing

Following publication, anonymised individual participant data will be made available upon request to the corresponding author for secondary research, conditional on assurance from the secondary researcher that the proposed use of the data is compliant with the Medical Research Council Policy on Data Sharing regarding scientific quality, ethical requirements, and value for money. Only data from participants who have consented for their data to be shared with other researchers will be provided. This also applies to proposals for access to the QRI qualitative data consisting of audio-recordings, transcripts of consultations and interviews. Proposals will need appropriate approvals in place, and the data will need to be de-identified before being shared to ensure anonymity and privacy for study participants and staff.
