# Supplementary material for: Roux-en-Y gastric bypass, adjustable gastric banding, or sleeve gastrectomy for severe obesity (By-Band-Sleeve): a multicentre, open label, three-group, randomised controlled trial
Source: Lancet Diabetes Endocrinol. Author manuscript; Available in PMC 2026 Jul 13. (PMC7619233; doi:10.1016/S2213-8587(25)00025-7)
Supplement: Supplementary material [file EMS214251-supplement-Supplementary_material.pdf]

THE LANCET
Diabetes & Endocrinology

Supplementary appendix

This appendix formed part of the original submission and has been peer reviewed. We post it as supplied by the authors.

Supplement to: The By-Band-Sleeve Collaborative Group. Roux-en-Y gastric bypass, adjustable gastric banding, or sleeve gastrectomy for severe obesity (By-Band-Sleeve): a multicentre, open label, three-group, randomised controlled trial. *Lancet Diabetes Endocrinol* 2025; published online Mar 31. https://doi.org/10.1016/S2213-8587(25)00025-7.

Appendix

CONTENTS

1 Key protocol changes during the trial 5
2 Rationale for the move from a two-group to a three-group trial 5
3 Sample size – additional information 6
4 Randomisation – additional information 6
5 Database and data handling procedures 7
6 Minimisation of bias 7
7 Study oversight 7
8 Participant demographics – additional information 7
9 Sources of missing data 8
10 Statistical methods – additional information 8
Derivation of outcomes 8
Analysis populations 8
Summary statistics 9
Modelling of longitudinal continuous outcomes 9
Modelling of binary outcomes 10
Modelling of count outcomes 10
Modelling of ordered multi-category outcomes 10
Accounting for clustering 10
Subgroup analyses and sensitivity analyses 11
Missing data 11
Significance levels and adjustment for multiplicity 11
11 Economic evaluation – additional information 12
Resource use and costs 12
Quality adjusted life years (QALYs) 13
Health economic analyses 13
Subgroup and sensitivity analyses 14
Reporting results 18
12 Supplementary cost-effectiveness results 18
13 Participant characteristics – participants recruited to the two-group and three-group phases of the trial 20

14 Participant characteristics – participants who underwent the allocated surgery, crossed over or didn't receive surgery 21
15 Impact of the COVID-19 pandemic on trial conduct 21
16 Additional Tables and Figures 22

Table 1 Reasons for potential participants not being eligible for the trial 22
Table 2 Baseline characteristics of trial participants – additional information - intention-to-treat population 23
Table 3 Key baseline characteristics of trial participants randomised in the two phases of the trial - intention-to-treat population 27
Table 4 Protocol deviations and crossovers from allocated surgery to another metabolic bariatric surgery - intention-to-treat population 29
Table 5 Key baseline characteristics of trial participants who received the allocated surgery 31
Table 6 Key baseline characteristics of trial participants who received an alternative surgery 32
Table 7 Key baseline characteristics of trial participants who did not receive surgery within three years of randomisation for any reason 33
Table 8 Impact of the COVID-19 pandemic on waiting times for surgery and hospital attendance for in-person follow-up - intention-to-treat population 34
Table 9 Surgical details – participants who underwent surgery 35
Table 10 Completeness of outcome data – number of participants included in statistical analyses 36
Table 11 Primary outcome – at least 50% excess weight loss at 3-years – intention to treat and per-protocol populations 38
Table 12 Primary outcome – EQ-5D utility score – intention to treat and per-protocol populations 40
Table 13 Percentage total weight loss at 3-years - intention-to-treat population 42
Table 14 Waist circumference at baseline and 3-years - intention-to-treat population 43
Table 15 HbA1c and antidiabetic medication at baseline and 3-years - intention-to-treat population 44
Table 16 Blood pressure, anti-hypertensive medication, total cholesterol and anti-hyperlipidaemic medication at baseline and 3-years - intention-to-treat population 45
Table 17 Vitamin D at baseline and 3-years - intention-to-treat population 45
Table 18 Other markers measured in blood at baseline and 3-years - intention-to-treat population 48
Table 19 Outcomes related to diet at 3 years - intention-to-treat population 50
Table 20 Liver fibrosis at 3 years - intention-to-treat population 51
Table 21 Epworth sleepiness scale at 3 years - intention-to-treat population 51
Table 22 Prevalence of sleep apnoea at baseline and 3 years - intention-to-treat population 52

Table 23 Medication and dietary supplements taken during follow-up - intention-to-treat population 53
Table 24 Quality of life questionnaire responses at baseline and 3 years - intention-to-treat population 55
Table 25 Anxiety and depression as assessed using the HADS questionnaire at baseline and 3 years - intention-to-treat population 57
Table 26 Causes of deaths within 3 years of randomisation - intention-to-treat population 57
Table 27 Surgery-specific morbidity – participants who underwent surgery 58
Table 28 Adverse events following surgery: surgery to 30 days – safety population 3 60
Table 29 Adverse events following surgery: 30 days post-surgery to three years – safety population 3 62
Table 30 Intraclass correlations for outcomes measured at multiple post randomisation time points 65
Table 31 Estimated costs (including intervention costs) and QALYs (primary analysis) 67
Table 32 Impact of sensitivity analyses on estimated costs including intervention costs 67
Table 33 Impact of sensitivity analyses on estimated difference in health-related quality of life 68
Table 34 Impact of alternative discount rates 69
Table 35 Estimated costs (including intervention costs) and QALYs - comparing participants randomised before and after start of COVID-19 pandemic 70

Figure 1 Proportion achieving 50% excess weight loss over time by randomised group 42
Figure 2 Weight loss over time by randomised group - intention-to-treat population 43
Figure 3 Outcomes measured in blood over time by randomised group - intention-to-treat population 46
Figure 4 Trajectories of generic quality of life outcomes over time - intention-to-treat population 54
Figure 5 Expected incremental net monetary benefit 71
Figure 6 Cost-effectiveness acceptability curves 71
Figure 7 Expected incremental net monetary benefit by body mass index and diabetes status at baseline 72
Figure 8 Expected incremental net monetary benefit by surgery received 73
Figure 9 Expected incremental net monetary benefit using a binary indicator to address a potential time-trend bias 73
Figure 10 Expected incremental net monetary benefit using a more recent mapping algorithm for deriving health-related quality of life 74

Figure 11 Expected incremental net monetary benefit using intervention costs as reported by NHS Cost Collection 75

1 Key protocol changes during the trial

The primary change, approved May 2015 (and heralded in the initial funding. application), implemented in August 2015, was from a two-group trial of Bypass versus Band to a three-group trial of Bypass versus Band versus Sleeve (Rogers et al Br J Surg. 2017 104(9):1207-1214). At the same time the eligibility criteria were updated in line with NICE guidance. Hiatus hernia >5cm was added as exclusion criterion. 218 participants had been recruited prior to this change.

Other changes included i) removal of the 3-month follow-up (March 2014) when the timing of follow-up was revised; initially timed from surgery, it was changed to follow-up from randomisation; it was anticipated that randomisation would occur once a surgery date was confirmed, but to facilitate recruitment and allow participants time to prepare for their surgery this was changed to after consent and before the surgery date was confirmed. This amendment to the protocol reduced the permitted lengths of the biliary and gastric limbs from 100cm and 200cm to 75cm and 150cm respectively, 96 participants had been recruited prior to this change; ii) removal of planned research staff blinded assessment of outcomes at 3-years as this was not feasible (June 2016); iii) mandated closure of iatrogenic mesenteric defects for participants having RYGB surgery (April 2018), prior to the change closure was optional; iv) option for follow-up visits to take place at home as well as at the hospital (introduced in response to the COVID-19 pandemic); v) review of secondary outcomes and their definitions to determine if they were still fit for purpose and to identify which of the 70+ outcomes would be compared statistically and which would be described (October 2022).

2 Rationale for the move from a two-group to a three-group trial

Review of the role of sleeve gastrectomy (SG) 18 months after the planned start of recruitment with the view of possibly adding as a third group was agreed with the funder when the research was commissioned. At the same time key criteria related to trial conduct were also reviewed (e.g. rates of eligibility, recruitment, compliance with allocated treatment and engagement with follow-up). Four sources of information were reviewed to determine whether the trial should be changed to include SG as a third group: data on current surgical practice; published comparative evidence and ongoing trials; expert surgical opinion (trial oversight committees, and specialist society); and published data on the stability of SG surgery. The review established that major changes in surgical practice in the UK had occurred since the research was funded; AGB had dropped from 35% to 11% and SG had increased from 8% to 24%. Evidence supporting the three procedures had not changed. Well designed and conducted RCTs comparing them were lacking. The experts supported the change of trial design to maintain the relevance of the research, and following independent peer-review, the

funder approved the change. All expert and independent advice recommended continuing to include AGB.

3 Sample size – additional information

The sample size was set to test both trial hypotheses (i.e. non-inferior weight loss and superior quality of life measured using the EQ-5D), both of which had to be met for RYGB/SG to be considered more effective than AGB and or SG to be considered more effective than RYGB. The sample size calculation assumed equal allocation to the study groups. The estimates underpinning the calculation (i.e. non-inferiority margin, proportion expected to achieve 50% excess weight loss at 3 years, target effect size for the EQ-5D and assumed correlations between repeated EQ-5D scores when comparing any two groups) remained unchanged when the trial design was changed from two to three groups. The estimates used for the EQ-5D comparisons were taken from the literature (target difference 0.06 points, assumed standard deviation 0.03). No allowance was made for clustering by centre. The sample size for the original two group trial was set at 724 participants (362 per group). The only change when the third group was added was that significance level for the non-inferiority comparison was reduced from 2.5% (one-sided) to 1% and from 5% (two-sided) to 2% for the superiority comparison to account for the three hypotheses. A conservative estimate of the overall study wise Type I error rate, not accounting for statistical correlation between treatment comparisons, for the three-group trial is 0.06%.

4 Randomisation – additional information

Access to the internet-based randomisation system was password controlled and only available to approved users. Randomisation was carried out by site research staff. The allocation was not revealed until sufficient information to identify the participant and the data needed for the minimisation algorithm had been entered. The minimisation algorithm was implemented by the system provider, Sealed Envelope Ltd. The probability of being assigned a treatment at random was set at 0.3 for the two-group trial and for centres joining after the design had been revised to include the third group. Allocations were 1:1 and 1:1:1 respectively. For centres that recruited during both the two-group and the three-group phases of the trial, simulations of the anticipated future recruitment were used to determine the centre-specific system parameters for the three-group phase (i.e. allocation ratio and probability of treatment being assigned at random). The parameters were chosen to ensure allocation concealment and as close to equal numbers in the three groups at the planned end of recruitment.

5 Database and data handling procedures

Data were collected by research staff at the study centres using purpose-designed case report forms and entered into a purpose-designed database built in Genesys by staff from the Trials Centre. The database included extensive validation (range and consistency checks) and generated data queries in real time. Additional data checks were undertaken by the study statistician. Regular reports were produced for the study team to monitor trial conduct. Participant-reported outcomes were collected via paper questionnaire or online. Questionnaires could be completed in clinic or at home (sent by post). Participants were contacted by telephone if questionnaires were not returned and were invited to complete the EQ-5D questionnaire over the telephone.

6 Minimisation of bias

Concealed randomisation protected against selection bias. Standard protocols for follow-up after all procedures were used to minimise the risk of performance bias arising from carers differentially providing co-interventions. Self-completion HRQOL measures were susceptible to bias although expectations about the effects of the different procedures prior to surgery are likely to have reduced with follow-up, so participants will not have strong differential expectations after three years. Outcome measures were defined as far as possible based on objective criteria. Biochemical markers were measured by an independent laboratory technician at the local hospital, without knowledge of treatment allocation.

7 Study oversight

The trial data monitoring and safety committee (see page 2 of this supplement for membership) met approximately every six months and at minimum annually throughout the trial to review the accumulating data. They reviewed recruitment, data quality, compliance with the protocol and participant safety. They also reviewed emerging evidence from the living systematic review about (now published in full: Chalmers KA, et al. Obes Rev. 2024;25(5):e13718). There were no planned or unplanned interim analyses of trial outcomes during the conduct of the trial.

8 Participant demographics – additional information

Participant sex and ethnicity were collected directly from the participant at the baseline visit. Participant gender identity was not collected.

9 Sources of missing data

Missing data arose when participants failed to attend the centre for follow-up, failed to complete the quality-of-life questionnaires and/or failed to respond to a phone call from the study team. Some blood measurements were also missing when a participant failed to fast before their follow-up visit (see later in this supplement details on how the COVID-19 pandemic impacted follow-up). The extent of missing data is documented in footnotes to the relevant tables. The number of participants contributing data to each outcome is given in Table 10 of this supplement. For most outcomes (i.e. except for outcomes relating to diet which was captured by interview and liver fibrosis at 3-years) at least 85% of participants were included in the analyses.

10 Statistical methods – additional information

The data are reported in line with the Consolidated Standards of Reporting Trials (CONSORT) reporting guidelines for superiority and non-inferiority trials.

Derivation of outcomes

Health-related quality of life (HRQoL) questionnaires were scored according to the developer's scoring instructions, summary scales derived from the questionnaires are reported. Dietary data collected from participant interviews were analysed using the nutrient analysis programme Dietplan7 (Forestfield Software Limited, version 7.00.47). Dietary intake is reported. Adverse events were all coded using the Medical Dictionary for Regulatory Activities (MedDRA) and are reported by system organ class and preferred term.

Analysis populations

All analyses exclude those who withdrew and were unwilling for data already collected to be used. Data from any participant who withdrew and was unwilling for their data to be used are included in the study flowchart and table documenting withdrawals but not in any subsequent data tables or figures. The analysis populations included the following participants:

a) *Intention to treat*: all randomised participants, grouped according to the randomised allocation, excluding participants who withdrew their consent for their data to be used (n=5)
b) *Per-protocol:* all randomised participants who received their allocated surgery within 3 years of randomisation (i.e. excluding participants who withdrew their consent for their data to be used, those who did not have surgery and those who crossed over and received an alternative surgery), grouped according to the randomised allocation

c) *Safety population 1:* randomised participants excluding participants who withdrew their consent for their data to be used (analysis of all events from randomisation to 3 years and analysis of events in the period from randomisation to surgery, participants grouped according to the allocation)

d) *Safety population 2*: randomised participants who underwent surgery within 3 years of randomisation excluding participants who withdrew their consent for their data to be used (analysis of events occurring after surgery to 30-days and from 30-days to 3 years after randomisation, participants grouped according to the allocation and according to surgery received)

Summary statistics

Data were described using summary statistics; mean and standard deviation for continuous variables (or median and interquartile range if distributions were skewed) and number and percentage for categorical variables. For outcomes transformed to the logarithmic scale for comparative analysis geometric means and coefficients of variation are also reported.

Modelling of longitudinal continuous outcomes

Time (i.e., date of visit, blood test, (HRQoL questionnaire completion, dietary review, etc.) was modelled as a restricted cubic spline with 4 knots placed at the study follow-up points (6, 12, 24 and 36 months), with separate splines fitted for each treatment group. This allowed for the modelling of non-linear surgery-specific responses over time, accounted for variation in the actual timing of follow-up assessments and allowed the primary HRQoL outcome to be estimated from surgery (e.g. 3-years after surgery) as well as from randomisation. Identity covariance structure at the centre level and unstructured covariance at the participant level were specified, with alternatives only examined if convergence failed. The variance-covariance matrix corresponding to parameter estimates was estimated using the likelihood-based observed information matrix and inference is based on a large-sample approximation of the sampling distributions of the test statistics by normal and chi-squared distributions (i.e. a small sample adjustment was not made). Model fit was assessed graphically (e.g. plots of predicted values versus standardised residuals and normal probability plots). If the model was a poor fit, transformations (e.g. log transformation for data following a log normal distribution) were considered. The impact of suppressing outliers was assessed, especially in cases where the data value was considered implausible. Treatment effects at 3-years were calculated as differences between marginal treatment means for values of the splines corresponding to 3-years, holding all other covariates at their means. Trajectories of outcomes over time by treatment group were

estimated from the models by calculating marginal means at each month since randomisation with time increments calculated across the three splines and other covariates held at their means.

Modelling of binary outcomes

Risk differences were estimated using a binomial model with an identity link. Robust standard errors clustered by centre were used to account for the nested structure of the data.

Modelling of count outcomes

Incidence rate ratios were estimated using negative binomial models with a log link, with centre included as a random effect. The analyses accounted for exposure time defined as the time from randomisation or surgery (depending on the model) to last follow-up, withdrawal or death as appropriate. The negative binomial model was chosen in preference to the Poisson model because over-dispersion was indicated for all outcomes.

Modelling of ordered multi-category outcomes

Ordered logistic regression was used to assess the treatment effects for the single ordered category outcome. The proportional odds assumption was checked. The data were too sparse to allow for site to be fitted as a random effect, so the clustered sandwich estimator was used to account for the nested structure of the data.

Accounting for clustering

As indicated in the main paper clustering by centre was accounted for by fitting centre as a random effect, except where not supported by the statistical software when robust standard errors clustered by centre were used. Clustering at the participant level (i.e. repeated measurements from the same individual) was also accounted for in the regression models fitted. Clustering by surgeon was not considered for two reasons; a) evidence from previous surgical trials (including those where the randomisation was expertise-based) suggested the intraclass correlation (ICC) at the surgeon level is negligible; b) the actual surgical procedure is only one aspect of the treatment, i.e. MBS is a complex intervention delivered by a team and the success of MBS is also dependent on co-interventions (e.g. follow-up care provided by physicians and dieticians). The complex nature of MBS is best captured by clustering by centre in a pragmatic trial. This approach is supported by a recent review (Conroy EJ et al. Managing clustering effects and learning effects in the design and analysis of randomised surgical trials: a review of existing guidance. Trials 2022; 23, 869). The ICCs at the centre and participant level for the study outcomes are given in Table 30 of this supplement.

Subgroup analyses and sensitivity analyses

Pre-specified subgroup analyses were implemented by adding a treatment by sub-group interaction term into the models comparing primary outcomes between groups. Sensitivity analyses for the primary outcomes are described in the results (Table 11 and Table 12 in this supplement).

Missing data

Prior to analysis the pattern of missingness was examined to determine whether the assumption of a missing at random (MAR) held. For longitudinal outcomes there is negligible benefit in imputing missing outcome data if it can be assumed to be MAR. If the missing data were assumed to be missing not at random (MNAR), imputation methods for MNAR outcomes were explored. Missing baseline values were imputed using the overall median or mean value, as appropriate, when missingness was less than 5%. Where missingness was 5% or more, multiple imputation was used to impute 50 baseline values via a regression on age, sex, site, diabetes status at baseline, and BMI category at baseline, with results combined using Rubin's rules. The exception to this rule were the dietary intake and ELF outcomes, as the level of missingness at baseline was deemed too high to support imputation (>20%). Dietary intake outcome data for a given participant was only included if a baseline and at least one follow-up measurement were available, while the ELF analysis was restricted to participants with measurements at both baseline and 36 months.

Missing BMI data at 3-years was imputed by chained equations. The model imputed data for all successive study follow-ups from 4 weeks post-surgery to 3-years post-randomisation and included age, sex, diabetes at baseline, time from randomisation to surgery/last follow-up for participants who did not receive surgery in months, baseline BMI category, and baseline BMI as independent variables. The imputation was performed on each treatment group separately, was truncated between the minimum and maximum BMI values observed in the overall dataset and used 50 imputations.

Significance levels and adjustment for multiplicity

Likelihood ratio tests were used in preference to Wald tests. No formal adjustment for multiplicity across outcomes was made beyond using 98% confidence intervals rather than 95% confidence intervals to reflect the three treatment comparisons; the number of comparisons made should be considered when interpreting results.

Note: There are minor differences between the published baseline data (By-Band-Sleeve Collaborative Group. Roux-en-Y gastric bypass, gastric banding, or sleeve gastrectomy for severe

obesity: Baseline data from the By-Band-Sleeve randomized controlled trial. Obesity (Silver Spring). 2023 May;31(5):1290-1299) and this publication due to further data cleaning before final data lock.

11 Economic evaluation – additional information

The primary within-trial cost-utility (a cost-effectiveness analysis with health-related quality of life as the health outcome) analysis focused on the first 3-years after randomisation and was performed using the intention-to-treat principle, effectively estimating the cost-effectiveness of making the decision to perform the surgery. It is important to note that this is different from the cost-utility of actually performing the surgery. The latter is explored in post-hoc analyses using randomisation as an instrumental variable as detailed in the subgroup and sensitivity analysis section. The CHEERS statement was adhered to. All health-economic analyses were performed in R version 4.0.1.

Resource use and costs

Resource use related to healthcare resource use was identified a priori based on literature searches and the Database of Instruments for Resource Use. Healthcare resource use was collected through case report forms completed at scheduled follow-up visits. It included details about the type of healthcare contact, relevant diagnosis and procedures, and corresponding dates. Data (dates and reason for visit) on all types of hospital visits (inpatient, outpatient, and accident & emergency visits, but excluding research visits) were obtained for the entire study period, increasing the likelihood that information on these events were recorded even if a patient missed a follow-up visit. Information on the total number of contacts with primary care was recorded since patient's last visit. Costs for the three surgical procedures were taken from a micro-costing study. The mean costs for each procedure across three sites that participated were used as intervention costs. The unit cost of hospital admissions, outpatient visits, accident and emergency visits were estimated by first assigning healthcare resource groups (HRG) and then assigning reference costs to the resources. For primary care visits, unit costs were obtained from the Personal Social Services Research Unit data and medication costs from the Drugs and pharmaceutical electronic market information tool (eMit) and the British National Formulary (BNF). The medication cost per patient was estimated by multiplying the cost per day to the number of days the medication was taken. The cost per day was based on actual dose prescribed. The e-MIT that has prices for generic drugs was the first source for obtaining medication costs. When the drug cost was not available in e-MIT, the BNF was used and the price adopted was the one that was under the brand name of the drug. The BNF was also used to obtain unit costs for the vitamin and mineral supplements recommended after RYGB or SG. Costs are

reported as 2021 prices and costs not reported in this year were inflated using the Hospital and Community Health pay and price index in England.

Quality adjusted life years (QALYs)

Quality adjusted life years (QALYs) were estimated using the EQ-5D-5L questionnaire. Health state utility values were derived by mapping the 5 level (5L) descriptive system data onto the English 3-level (3L) value set. While in the main analysis, results were obtained using the interim mapping proposed by Van Hout et al. in line with the analysis plan (van Hout B, Janssen MF, Feng Y-S et al. Interim Scoring for the EQ-5D-5L: Mapping the EQ-5D-5L to EQ-5D-3L Value Sets. Value in Health 2012; 15 708-715) in post-hoc sensitivity analysis the more recently developed mapping function developed by the Decision Support Unit and since recently recommended by NICE was used. (Wailoo A, Alava MH, Pudney S, Barton G, O'Dwyer J, Gomes M, Irvine L, Meads D, Sadique Z. An International Comparison of EQ-5D-5L and EQ-5D-3L for Use in Cost-Effectiveness Analysis. Value Health. 2021 Apr;24(4):568-574). In the main analysis we assumed that HRQoL changes linearly between visits, where visits that occurred within 45 days of their planned date were assigned the date of the scheduled visit (i.e. 4 weeks post-surgery, 6 months, 1, 2 or 3-years post randomisation). Where there were no measurements within 45 days before or after a scheduled visit, we used multiple imputation to impute the HRQoL at the time of the scheduled visit. Utility at the time of death was estimated by linearly interpolating between participant’s last observed utility and the utility imputed in place of the following scheduled questionnaire using multiple imputation. For participants who died, their utility was set to zero after dying.

Health economic analyses

The main analyses included, estimating the within-trial cost-utility of being allocated to the different surgeries of interest, all randomised patients, excluding patients withdrawn who did not consent for their data collected to be used. QALYs gained were monetised by multiplying them by the willingness-to-pay (WTP) per QALY gained. The individual components of the net monetary benefit (NMB) are the total (discounted) costs and QALYs. For the main analyses linear regression was used for both costs and QALYs. Potential correlation between costs and QALYs was accounted for using non-parametric bootstrapping. To ensure the non-parametric bootstrap approximates the actual randomisation process including the minimization, trial participants were resampled within each ‘allocated treatment-diabetes-BMI’ group with the total number of (re)sampled participants equal to the percentage of people within each category in the trial. Through resampling patients instead of observations, correlation between observations within patients is taken into account. Missing data in terms of HRQoL estimates were dealt with by nesting a single imputation within each of the 500

bootstraps, which has been shown to be a strategy with good statistical properties for cost-effectiveness analyses in simulation studies (Brand J, van Buuren S, le Cessie S, van den Hout W. Combining multiple imputation and bootstrap in the analysis of cost-effectiveness trial data. Stat Med. 2019 Jan 30;38(2):210-220). This number of bootstraps ensured that the analyses, including a wide range of sensitivity analyses, were computationally feasible. Importantly, when rerunning the script with different seed virtually identical results were obtained, meaning that 500 bootstraps with a single imputation nested within each bootstrap is sufficient. The single imputations were obtained using multiple imputation models implemented using chained equations, implemented separately by allocated treatment, included the covariates included in the regression models, as well as the following auxiliary variables: age, sex, ethnicity, employment status at baseline, income band, receipt of benefits, height, weight, smoking, and timing of surgery. In line with that simulation study, we used standard percentile bootstrap method to estimate confidence intervals, although in sensitivity analysis we explored the potential benefits of bias-corrected and accelerated bootstrap (see sensitivity analysis section for more detail).

In the main analyses we assumed healthcare resource utilisation information was complete, thereby assuming medication and supplementation use would continue until the end of follow-up unless there was evidence that the patient stopped the drug/supplement of interest. Cost-effectiveness acceptability curves were used to show the probability of each intervention to be the most cost-effective option. In addition, we estimated the net loss statistic (opportunity cost), which is estimated as the difference in the incremental NMB of intervention of interest and incremental NMB of the cost-effective intervention (i.e. the intervention with the highest NMB).

Subgroup and sensitivity analyses

The subgroup analyses followed the clinical analyses. Subgroup effects were investigated by adding subgroup by allocated surgery interaction terms to the regression models described above. Several pre-specified sensitivity analyses were performed to evaluate the robustness of the cost-utility results.

1. Using a one-inflated beta regression model to deal with observations with a utility score of 1. Because negative utility scores can be observed, values less than one were first normalised so that they fell between (including) 0 and 1. Next they were transformed to ensure they fell between (excluding) 0 and 1: $(score_i \times (N-1) + 0.5) / N$, where N equals the sample size.
2. Using restricted maximum likelihood (REML)-based mixed effects linear models to estimate the impact on QALYs gained using i) a random intercept model assuming a diagonal covariance

structure with time as modelled categorically as in the main analysis and otherwise the same covariates as in the main analysis; ii) a similar random intercept, but with time modelled using a restricted cubic spline with one inner knot placed at 365 days and boundary knots placed at 182 and 730 days; or iii) a random intercept and a random slope on the spline for time, assuming an unstructured covariance matrix.

3. Using a generalised linear model with a gamma distribution and log link to model costs.
4. Assuming healthcare utilisation and costs were missing or incomplete if no weight measurement was provided at a visit, censoring participants from the time-point they had a missing weight measurement and onwards for the estimation of healthcare costs and using inverse probability of censoring weighting – using the same variables as used for the multiple imputation – to account for informative censoring. This analysis ignored any resource utilisation occurring after a missing weight measurement, even if reported. This was done as it is challenging for those that do not report healthcare utilisation to decide whether they did not have relevant healthcare contact or whether they did not report that they have healthcare contact.
5. Grouping participants according to their received surgery rather than the allocated surgery.
6. Estimate the impact of the COVID-19 pandemic, by comparing participants randomised before or after the start of the pandemic, taken to be 16 March 2020. While in the protocol the split between participants was proposed to happen based on having reached the 3 year endpoint before vs after the start of the pandemic, however after further discussion it was decided that the strategy implemented relate to an actual decision to make: are we going to schedule someone for bariatric surgery during a pandemic or not instead of relating to the decision to something that has not happened yet at the moment of decision making.
7. Assess the impact of alternative discount rates: 0 and 5% for both costs and QALYs; and using differential discounting: 0% for QALYs and 3.5% for costs, acknowledging that differential discounting is only accepted in a small number of countries and not (anymore) in the UK.
8. Exploring the potential impact of the long-term need of nutritional supplementation on the longer-term cost-utility over a 20-year time-horizon. Based on the observation that differences in measured costs (excluding intervention costs), QALYs, and several risk-factors of metabolic or cardiovascular disease – including weight loss, fasting glucose, HbA1c, triglycerides, HDL-C – if anything, widened in favour of RYGB over the 3 years within the trial follow-up, this sensitivity analysis was kept simple and conservatively assumed that those still alive after 3 years of follow-up would have perfect adherence to recommended nutritional supplementation – to keep preventing nutritional deficiencies after follow-up – for all surgeries without accruing any further

differences over the 20-year horizon. For these analyses, we assumed participants would use the supplementation recommended by NHS foundation trusts.

9. Assessing the incremental NMB using percentage total weight loss (%TWL) instead of QALYs, using a wide range for the WTP for a %TWL, given the latter is not known/defined.

We pre-specified a further sensitivity analysis that would include hospitalisation within a 2-month interval (flagging hospitalizations if they occurred ±30 days from EQ-5D questionnaire date; a 60-day window) as a covariate in the imputation models to capture potential temporary reductions in HRQoL due to acute illness related to the hospitalisations that would not be captured in participants that are hospitalised e.g., 100 days after the 1 year follow-up questionnaire. However, this resulted in too many variables included for the imputation model to converge. In addition, we originally planned to estimate the impact on income and receipt of benefits, however due to the high amount of missing data at 3 years (more than 50%) we decided not to perform these analyses.

The following sensitivity analyses were not pre-specified but added to further assess the robustness of the results.

1. Using a binary indicator for the period before vs the date of the introduction of SG to the study to assess to what extent results of the main analysis might be biased due to time-trends affecting results, where e.g. outcomes improved over time and the fact that patients could only be randomised to SG after the addition of this group to the already ongoing trial.

2. Using the more recently developed mapping function developed by the Decision Support Unit and since recently recommended by NICE. (Wailoo A, Alava MH, Pudney S, Barton G, O'Dwyer J, Gomes M, Irvine L, Meads D, Sadique Z. An International Comparison of EQ-5D-5L and EQ-5D-3L for Use in Cost-Effectiveness Analysis. Value Health. 2021 Apr;24(4):568-574)

3. Using the estimated intervention costs from the 2021 NHS cost collection (£7,860 for RYGB, £6,779 for SG, and £4,031 for AGB) instead of inflating the micro-costing costs, reflecting that the former costs are substantially higher (https://www.england.nhs.uk/costing-in-the-nhs/national-cost-collection/).

4. A per protocol analysis restricted to patients who received their allocated surgery within the 3 years of follow-up.

5. Because the above per protocol analysis is potentially biased as it ‘breaks’ randomisation and to estimate the cost-utility of performing the surgeries (instead of making the decision to perform the surgery as implicitly estimated in the main analysis), an additional analysis estimating the cost-utility of performing the three different surgeries at t=0, while restricting to those that received their

allocated surgery within the 3 years of follow-up (compliers), was performed. For this analysis randomisation itself was used as an instrumental variable for receipt of surgery, whereas time-to-surgery was included as a linear covariate given that restricted cubic splines all resulted in worse model fit. Effects on QALYs and costs were estimated using 2-stage least-squares (2SLS) regression, with the first stage focusing on whether patients ever received surgery during follow-up while adjusting for baseline EQ5D, diabetes and BMI at baseline, treating centre, and an indicator to distinguish between the period before and after the addition of Sleeve to the trial as covariates. The second stage estimated the effect on EQ5D / costs over time using the same covariates plus calendar time as a covariate. In addition, a time-to-surgery variable was included as a covariate, which was used to predict the EQ5D and costs if all compliers would have received their surgery at t=0 during the period that patients could be randomised to Sleeve. This analyses is done under the assumption that randomisation is and does not violate the exclusion restriction (i.e. assuming randomisation only has an influence on the outcome through influencing the probability of receiving each surgery of interest) and that time to surgery is largely driven by availability of surgeons and/or operation theatre which can be adjusted for by controlling for centre in the regression analysis; whereas any remaining variation in time to surgery is likely driven by random events such as patients going on holiday. Importantly, the time to surgery is very similar between the different randomisation groups. This modelling approach could only estimate the average effect over the three-year period, implicitly assuming that the effects of surgery are constant over time which is not completely true and will lead to a small bias due to discounting having a larger impact in year 3 than in year 1. To be able to estimate treatment effects over time, another instrumental variable would have been needed, for which no valid instrument could be found in this instance without likely violating the exclusion restriction. To be able to get as close as possible to causal estimates of the health-economic effects of performing the surgeries this analysis also used inverse probability of censoring weighting to account for potential missing healthcare costs after the first-time patients had a missing weight measurement.

7. We explored the potential benefits of bias-corrected and accelerated bootstrap by creating a dataset with expected values for missing data by taking the mean values (mode for factors) of 100 multiply imputed datasets. We compared point estimates on this dataset with the point estimate based on the mean of the bootstrap samples using the percentile method and estimated the acceleration parameter through a jack-knife procedure leaving one participant out at a time.

Reporting results

In line with the statistical analyses, 98% CIs were used for the pairwise comparisons to account for the three comparisons. For other estimates 95% CIs were reported. Confidence intervals were estimated form the bootstraps using the percentile method.

12 Supplementary cost-effectiveness results

Combining these effects on healthcare costs and HRQoL and their uncertainties into an incremental NMB analysis, RYGB was the most cost-effective option at both the lower and upper limit (£20,000 and £30,000 per QALY, respectively) of the cost-effectiveness threshold generally applied by NICE when identifying cost-effective interventions with the incremental NMB of RYGB (versus AGB) ranging between £3,149 and £5,241, whereas these values ranged between £2,288 and £3,587 for SG (versus GB) (Figure 5). When using these same thresholds, the probability that the intervention with the highest expected incremental NMB is also the most cost-effective option is relatively high for RYGB at £20,000 and £30,000 per QALY (0.75 and 0.81, respectively), with a zero probability for AGB being the most cost-effective option at these thresholds (Figure 6).

RYGB remained the most cost-effective option, with the highest incremental NMB, across the subgroup analyses by BMI (<40kg/m^2, 40-50kg/m^2, and >50kg/m^2) and diabetes (Figure 7) and when grouping participants by the surgery received (Figure 8) at the £20,000 and £30,000 per QALY thresholds. Using different statistical approaches to estimate QALYs gained or healthcare costs - including gamma regression for costs, varying mixed effects models for estimating QALYs, one-inflated beta-regression for QALYs - incurred during the trial follow-up had little impact on the results (Table 31 and Table 32). The most noticeable difference was observed when using one-inflated beta regression for QALYs instead of a linear model which resulted in smaller differences between surgeries (RYGB versus AGB 0.13, 98 CI 0.02 to 0.24 instead of 0.20, 98% CI 0.08 to 0.31; SG versus AGB 0.10, 98% CI 0.01 to 0.21 versus 0.13, 98% CI 0.02 to 0.27; SG versus RYGB -0.03 (98% CI -0.13 to 0.07) instead of -0.07 (98% CI -0.20 to 0.05). When artificially censoring participants from the first moment they had a weight measurement, resulting in 14%, 16%, and 11% of patient randomised to AGB, SG, and RYGB being censored at one point in time, and using inverse probability weighting to address potential missing healthcare utilisation and associated costs also led to similar results as the main analysis.

While using alternative discount rates inevitably led to slightly different total costs and QALYs accrued by allocated treatment (Table 33), the expected incremental NMB remained favourable for RYGB at

the £20,000 per QALY threshold with higher incremental NMB estimates for bypass than for sleeve (£3,317 vs. £2,427 with a non-differential discount rate of 0%, £3,028 vs. £2,260 with a non-differential discount rate of 5%, and £3,296 vs £2,422 with a discount rate of 0% for QALYs and 3.5% for costs, respectively).

When comparing patients randomised before the start of the COVID-19 pandemic, defined as 23 March 2020, or on or after the date of the start of the pandemic, those randomised before the pandemic accrued slightly higher number of QALYs, a difference most pronounced for those allocated to RYGB (2.04, 95% CI 1.95-2.12 vs. 1.96, 95% CI 1.87, 2.02; Table 26). Mean cost per participant were lower for those randomised on or after the start of the pandemic, except for those allocated to RYGB where costs were estimated to remain similar (£8,207, 95% CI £7,418-£9,182 after the start of the pandemic vs £8,277, 95% CI £7,685-£8,930; Table 34). These differences might be partly explained by the increasing waiting time for all surgeries, which was most pronounced for RYGB where median waiting time increased from 146 days to 189 days, whereas these figures changed from 146 days to 172 days for SG and from 110 days to 165 days for AGB. Cross-over to other surgeries was also more common for AGB after the start of the pandemic. Before the pandemic started, of those allocated to AGB, 6% received SG and 6% RYGB, whereas these numbers were 11% and 7% among those allocated to AGB after the start of the pandemic. Due to larger difference in the mean costs per participant and smaller difference in terms of QALYs gained, for patients randomised after the start of COVID-19, SG was estimated to be the most cost-effective option after the start of the pandemic at the £20,000 per QALY threshold (NMB for SG £1,372 vs. £1,050 for RYGB, with a 55% probability that SG was the most cost-effective option versus a 40% probability that RYGB was the most cost-effective option). This is in contrast to the period before COVID-19, where RYGB is clearly the most cost-effective option due to larger differences in QALYs gained and smaller differences in the mean healthcare costs per patient (NMB for SG £2,201 vs £3,943 for RYGB, with a 90% probability that RYGB is the most cost-effective option, whereas this probability was only 10% for SG).

The sensitivity analyses that were not pre-specified (see above), all led to similar results as the main analysis (Figures 9-11). Focusing on %TWL and ignoring QALYs gained, effectively performing a cost-effectiveness instead of a cost-utility analysis, RYGB was estimated to be the most cost-effective option if the WTP per %TWL reduction (26.8% for RYGB, 19.4% for SG, 14.0% for AGB, Table 23) is at least £75. However, it is important to note that this secondary analysis does not value relevant differences in HRQoL between the different groups. Indeed, the gap in measured in HRQoL was widening over the years within the first 3 years of follow-up (Figure 2 in main text). When assuming

perfect adherence to nutritional supplementation in all groups between year 4 and year 20, while conservatively – given the widening of difference between groups over time - not modelling any differences in health outcomes beyond year 3 between the allocated surgeries, these additional required supplementation costs would be higher for RYGB and SG (£1,217) than for AGB (£415). However, adding this difference (£802) is smaller than the difference in incremental NMB of the main analysis at a threshold of 20,000 per QALY (£3,149 for RYGB and £2,288 for SG), meaning that conclusion of the main analysis still holds after adding longer-term nutritional supplementation costs. However, longer-term follow-up is needed to estimate the longer-term cost-utility beyond 3 years more accurately.

Instrumental variable based analysis estimating the cost-utility of performing surgery at the start of follow-up for everyone led to larger differences in total healthcare costs (Table 31) due to the fact that everyone would receive surgery immediately, slightly more pronounced differences between the surgeries in terms of QALYs gained (Table 32), resulting in the same health-economic conclusions with e.g. a negative increment NMB of sleeve compared to bypass at 20,000 per QALY (£-933).

While all analyses use simple percentile bootstrap for analyses focusing on estimating confidence intervals, we assessed whether bias-corrected and accelerated bootstrap would have been better. Using a dataset with missing data replaced by their expected value based on 100 multiple imputed datasets, for QALYs, the point estimate was identical compared to the point estimate obtained through the percentile bootstrap (difference of 0.00), whereas the acceleration parameter was estimated at -0.01, suggesting no benefit of using bias-corrected and accelerated bootstrap over using the percentile bootstrap method. For costs, the point estimate using the expected value dataset was nearly identical compared to the point estimated obtained through the percentile bootstrap (difference < 1%) and the acceleration parameter was estimated to be 0.04, suggesting negligible benefit of using bias-corrected and accelerated bootstrap for estimating confidence intervals.

13 Participant characteristics – participants recruited to the two-group and three-group phases of the trial

Generally, the characteristics of participants recruited in the two phases are similar (Table 3 of this supplement). Exceptions are that a higher proportion of women (78% versus 67%) and a lower proportion of people living with diabetes (29% vs 38%) participated in the second three-group phase. There were also some ethnic differences; a higher proportion of people from Asian/Asian British or

Black/African/Caribbean/ Black British groups participated (1% vs. 4% and 1% vs 8% respectively). This may be explained by the geographical differences in participating centres although sensitivity analyses adjusting for design phase did not alter the findings.

14 Participant characteristics – participants who underwent the allocated surgery, crossed over or didn't receive surgery

Again, generally, the characteristics of participants who received the allocated surgery, crossed over or did not have surgery were similar (Tables 5, 6 and 7 of this supplement). The most notable difference is in ethnicity; proportionally more people from several ethnic minority groups did not receive surgery within three years of randomisation, for example, 12% of the Black/African/Caribbean and Black British participants recruited never received surgery.

15 Impact of the COVID-19 pandemic on trial conduct

Recruitment to the trial was completed before the pandemic, but not all participants had received their surgery. Those that were still waiting for surgery had a significantly longer wait than those operated before the pandemic (see Table 8 in this supplement). Only 25 (2.1%) of the 1183 surgeries took place after the pandemic and the numbers were well-balanced across the three groups. Of the 1346 randomised participants, 191 withdrew, died or were lost-to-follow-up within three years (see Figure 1). Of the remaining 1160, 610 (52.6%) reached the primary endpoint at three years before the pandemic, and 550 (47.4%) were in follow-up at the start of the pandemic (taken as 20 March 2020). The pandemic prevented these participants attending hospital for study follow-up appointments, and this was reflected in a higher proportion of follow-ups being completed by telephone for the primary outcomes and lower rates of completion for secondary outcomes that required an in-person appointment (i.e. outcomes measured in blood samples). Overall, loss-to-follow-up for the primary outcomes was in line with rate assumed in the power calculation.

16 Additional Tables and Figures

Table 1 Reasons for potential participants not being eligible for the trial

**Ineligible**	**2221 (39.6%)**
Under 18 years	20 (0.9%)
Did not meet NICE guidelines	57 (2.6%)
Not fit for anaesthesia and surgery	134 (6.0%)
Not committed to follow-up	415 (18.7%)
Not able to provide written informed consent	109 (4.9%)
History of previous gastric surgery for severe and complex obesity	394 (17.7%)
Large abdominal ventral hernia	84 (3.8%)
Crohn's disease	29 (1.3%)
Liver cirrhosis and/or portal hypertension	41 (1.8%)
Systemic lupus erythematosus	21 (0.9%)
Known silicone allergy	13 (0.6%)
Other clinical/ psychological reason	952 (42.9%)
Active participation in another interventional study	56 (2.5%)
Pregnancy*	32 (1.4%)
Previous abdominal surgery precluding one or more of the surgeries	178 (8.0%)
Hiatus hernia > 5cm	90 (4.1%)

Table 2 Baseline characteristics of trial participants – additional information - intention-to-treat population

	Randomised to RYGB (n = 462)	Randomised to AGB (n = 464)	Randomised to SG (n = 420)	Overall (n = 1346)
Neck circumference[1]	42.0 (39.0, 46.0)	42.0 (39.5, 46.0)	42.0 (39.0, 45.0)	42.0 (39.0, 46.0)
ASA class				
I: Healthy, no medical problems	93/458 (20%)	93/463 (20%)	91/418 (22%)	277/1339 (21%)
II: Mild systemic disease	229/458 (50%)	222/463 (48%)	211/418 (50%)	662/1339 (49%)
III: Severe systemic disease, but not incapacitating	128/458 (28%)	140/463 (30%)	113/418 (27%)	381/1339 (28%)
IV: Severe systemic disease that is a constant threat to life	8/458 (2%)	8/463 (2%)	3/418 (1%)	19/1339 (1%)
Taking any medication	424/461 (92%)	429/464 (92%)	379/419 (90%)	1232/1344 (92%)
Epworth sleepiness scale[2]	5 (3, 9)	4 (2, 8)	5.0 (2.0, 10.0)	5.0 (2.0, 9.0)
Asthma	127/460 (28%)	150/464 (32%)	137/419 (33%)	414/1343 (31%)
**Cardiovascular history**				
Angina	6/461 (1%)	11/464 (2%)	8/419 (2%)	25/1344 (2%)
Myocardial infarction	4/461 (1%)	6/464 (1%)	7/419 (2%)	17/1344 (1%)
Coronary artery bypass graft	0/461 (0%)	2/463 (<1%)	1/419 (<1%)	3/1343 (<1%)
Stroke	9/461 (2%)	13/464 (3%)	5/419 (1%)	27/1344 (2%)
Claudication	4/461 (1%)	11/464 (2%)	13/419 (3%)	28/1344 (2%)
NYHA classification				
I	357/459 (78%)	349/463 (75%)	304/419 (73%)	1010/1341 (75%)
II	74/459 (16%)	84/463 (18%)	96/419 (23%)	254/1341 (19%)
III	26/459 (6%)	27/463 (6%)	14/419 (3%)	67/1341 (5%)
IV	2/459 (<1%)	3/463 (1%)	5/419 (1%)	10/1341 (1%)
**Other past history**				
Symptomatic history of gallstones	75/460 (16%)	80/419 (19%)	95/464 (20.5%)	250/1343 (19%)
Previous cholecystectomy	58/460 (13%)	57/419 (14%)	74/464 (16%)	189/1343 (14%)
Deep vein thrombosis or pulmonary embolism	22/460 (5%)	27/419 (6%)	31/464 (7%)	80/1343 (6%)
Peptic ulcer	16/460 (3%)	10/418 (2%)	8/464 (2%)	34/1342 (3%)
Helicobacter pylori status checked	145/460 (32%)	102/417 (24%)	142/464 (31%)	389/1341 (29%)
Positive result	29/462 (6%)	19/420 (5%)	24/465 (5%)	72/1347 (5%)
Number of previous conceptions[3]	2 (1, 3)	2 (1, 4)	2 (1, 3)	2 (1, 3)
Number of children[4]	2 (0, 2)	2 (1, 3)	2 (1, 3)	2 (1, 3)

	Randomised to RYGB (n = 462)	Randomised to AGB (n = 464)	Randomised to SG (n = 420)	Overall (n = 1346)
Treatment for fertility?	25/459 (5%)	22/464 (5%)	27/418 (6%)	74/1341 (6%)
**Medication and supplements taken**				
Anti-depressant medication	165/462 (36%)	190/464 (41%)	153/420 (36%)	508/1346 (38%)
Asthma medication	94/424 (22%)	114/429 (27%)	107/379 (28%)	315/1232 (26%)
Medication for gastro-oesophageal reflux disease	158/424 (37%)	187/429 (44%)	143/379 (38%)	488/1232 (40%)
Anti-coagulants	8/424 (2%)	9/429 (2%)	9/379 (2%)	26/1232 (2%)
Analgesia	216/424 (51%)	219/429 (51%)	204/379 (54%)	639/1232 (52%)
Other medication	254/424 (60%)	263/429 (61%)	234/379 (62%)	751/1232 (61%)
Dietary supplements	179/424 (42%)	165/429 (38%)	142/379 (37%)	486/1232 (39%)
**Weight loss methods attempted**				
Orlistat	309/461 (67%)	272/419 (65%)	319/464 (69%)	900/1344 (67%)
Sibutramine	36/460 (8%)	22/419 (5%)	32/464 (7%)	90/1343 (7%)
Intra-gastric balloon	4/461 (1%)	6/419 (1%)	4/464 (1%)	14/1344 (1%)
Rimonbant	7/460 (2%)	4/419 (1%)	7/464 (2%)	18/1343 (1%)
Topiramate	7/460 (2%)	2/419 (<1%)	5/464 (1%)	14/1343 (1%)
Very low-calorie diet	313/461 (68%)	271/419 (65%)	304/464 (66%)	888/1344 (66%)
High protein/low carbohydrate diet	209/461 (45%)	181/419 (43%)	208/464 (45%)	598/1344 (44%)
Commercial slimming clubs	372/461 (81%)	349/419 (83%)	382/464 (82%)	1103/1344 (82%)
Medical management clinic	375/461 (81%)	334/419 (80%)	365/464 (79%)	1074/1344 (80%)
Metformin	77/461 (17%)	65/419 (16%)	97/464 (21%)	239/1344 (18%)
Other	154/461 (33%)	129/419 (31%)	166/463 (36%)	449/1343 (33%)
**Functional status**				
Can climb 3 flights of stairs without resting	194/458 (42%)	197/463 (43%)	177/419 (42%)	568/1340 (42%)
Can climb 1 flight of stairs without resting	187/458 (41%)	179/463 (39%)	156/419 (37%)	522/1340 (39%)
Can climb half a flight of stairs without resting	61/458 (13%)	74/463 (16%)	71/419 (17%)	206/1340 (15%)
Requires wheelchair or housebound	16/458 (3%)	13/463 (3%)	15/419 (4%)	44/1340 (3%)
**Employment status**				
Full time	191/460 (42%)	188/464 (41%)	182/419 (43%)	561/1343 (42%)
Part time	81/460 (18%)	67/464 (14%)	69/419 (16%)	217/1343 (16%)
Self employed	25/460 (5%)	27/464 (6%)	16/419 (4%)	68/1343 (5%)
Homemaker	39/460 (8%)	50/464 (11%)	40/419 (10%)	129/1343 (10%)
Student	5/460 (1%)	5/464 (1%)	4/419 (1%)	14/1343 (1%)
Retired	44/460 (10%)	33/464 (7%)	31/419 (7%)	108/1343 (8%)

	Randomised to RYGB (n = 462)	Randomised to AGB (n = 464)	Randomised to SG (n = 420)	Overall (n = 1346)
Unable to work	43/460 (9%)	62/464 (13%)	50/419 (12%)	155/1343 (12%)
Unemployed	32/460 (7%)	32/464 (7%)	27/419 (6%)	91/1343 (7%)
**Income and benefits**				
Total Income/benefits (£1000)	28 (19.404, 45)	26.4 (17.285, 45)	26.774 (17.847, 45)	26.870 (18.259, 45)
Unknown/unwilling to say	93/461 (20%)	101/464 (22%)	78/419 (19%)	272/1344 (20%)
Any benefits	179/461 (39%)	204/464 (44%)	165/419 (40%)	548/1344 (41%)
Disability benefits	106/461 (23%)	123/464 (27%)	97/419 (23%)	326/1344 (24%)
Child benefits	46/461 (10%)	48/464 (10%)	37/419 (9%)	131/1344 (10%)
**Blood measurements**				
Fasting glucose (mmol/L)[5]	5.2 (4.7, 6.2)	5.2 (4.7, 5.8)	5.1 (4.7, 5.9)	5.2 (4.7, 5.9)
LDL-Cholesterol (mmol/L)[6]	2.8 (2.2, 3.4)	2.9 (2.2, 3.5)	2.9 (2.3, 3.5)	2.9 (2.3, 3.5)
Red blood cells (10^{12}/L) [7]	4.8 (4.5, 5.1)	4.8 (4.5, 5.1)	4.8 (4.6, 5.1)	4.8 (4.5, 5.1)
Haematocrit (L/L)[8]	0.45 (0.27)	0.42 (0.04)	0.43 (0.19)	0.43 (0.19)
Haemoglobin (g/dL)[9]	13.9 (13.0, 14.7)	13.9 (13.0, 14.6)	13.8 (13.1, 14.6)	13.9 (13.0, 14.7)
Mean cell haemoglobin (pg)[7]	29.0 (27.8, 30.1)	28.9 (27.7, 30.0)	29.1 (27.6, 30.2)	29.0 (27.7, 30.1)
Mean cell volume (fL)[7]	88.4 (84.9, 91.3)	88.0 (84.6, 91.2)	88.1 (84.3, 91.5)	88.2 (84.6, 91.3)
MCHC (g/dL)[10]	32.7 (31.8, 33.5)	32.8 (31.9, 33.5)	32.7 (31.9, 33.5)	32.7 (31.9, 33.5)
Lymphocytes (10^9/L) [11]	2.2 (1.8, 2.7)	2.2 (1.8, 2.6)	2.1 (1.7, 2.6)	2.2 (1.8, 2.6)
Neutrophils (10^9/L) [12]	4.5 (3.6, 5.8)	4.6 (3.7, 5.7)	4.5 (3.6, 5.6)	4.5 (3.7, 5.7)
Alanine aminotransferase (IU/L)[13]	23.0 (18.0, 32.0)	24.0 (17.5, 33.0)	23.0 (17.0, 32.5)	23.0 (18.0, 32.0)
Alkaline phosphatase (IU/L)[14]	81.0 (69.0, 97.0)	81.0 (68.0, 98.0)	82.0 (69.0, 96.0)	81.0 (68.0, 97.0)
Bilirubin (µmol/L) [15]	8.0 (6.0, 11.0)	8.0 (7.0, 11.0)	9.0 (7.0, 12.0)	8.0 (7.0, 11.0)
Albumin (g/L)[16]	40.4 (3.8)	40.3 (3.8)	40.0 (3.8)	40.2 (3.8)
Total protein (g/L)[17]	72.0 (69.0, 74.0)	72.0 (69.0, 75.0)	72.0 (69.0, 75.0)	72.0 (69.0, 75.0)
C-reactive protein (mg/L)[18]	7.0 (3.7, 12.0)	7.0 (4.0, 13.0)	7.1 (4.0, 13.0)	7.0 (4.0, 12.0)
Sodium (mmol/L) [19]	139.0 (138.0, 141.0)	139.0 (138.0, 141.0)	140.0 (138.0, 141.0)	139.0 (138.0, 141.0)
Potassium (mmol/L) [20]	4.3 (4.1, 4.5)	4.3 (4.1, 4.5)	4.3 (4.1, 4.5)	4.3 (4.1, 4.5)
Urea (mmol/L) [21]	4.9 (3.9, 5.8)	4.6 (3.9, 5.7)	4.7 (4.0, 5.7)	4.8 (3.9, 5.8)
Creatinine (µmol/L)[22]	66.0 (58.0, 74.0)	66.0 (58.0, 75.0)	65.0 (59.0, 72.0)	66.0 (59.0, 74.0)
Magnesium (mmol/L) [23]	0.8 (0.8, 0.9)	0.8 (0.8, 0.9)	0.8 (0.8, 0.9)	0.8 (0.8, 0.9)
Serum iron (µmol/L)[24]	13.0 (10.0, 17.0)	12.8 (10.0, 16.0)	13.0 (10.0, 16.6)	13.0 (10.0, 16.5)
Ferritin (µgl/L)[25]	59.0 (30.0, 109.0)	67.0 (35.0, 114.0)	71.0 (33.0, 131.5)	66.0 (32.0, 116.0)
Vitamin B12 (ng/L)[26]	308.0 (235.0, 417.0)	296.0 (226.0, 398.0)	319.5 (245.0, 415.5)	310.0 (233.0, 415.0)
Folate (µgl/L)[27]	6.5 (4.5, 9.3)	5.9 (4.2, 8.8)	5.9 (4.3, 9.2)	6.1 (4.3, 9.2)
25-hydroxyvitamin D (nmol/L)[28]	42.4 (28.0, 59.0)	44.0 (30.0, 60.6)	42.8 (29.5, 61.1)	43.0 (29.0, 60.0)
Parathyroid hormone (pmol/L) [29]	5.7 (4.4, 7.9)	5.8 (4.2, 7.9)	5.8 (4.3, 7.9)	5.8 (4.3, 7.9)

	Randomised to RYGB (n = 462)	Randomised to AGB (n = 464)	Randomised to SG (n = 420)	Overall (n = 1346)
Calcium (mmol/L) [30]	2.4 (2.3, 2.4)	2.4 (2.3, 2.4)	2.4 (2.3, 2.4)	2.4 (2.3, 2.4)
Phosphate (mmol/L) [31]	1.1 (1.0, 1.2)	1.1 (1.0, 1.2)	1.1 (0.9, 1.2)	1.1 (1.0, 1.2)

Data are presented as median (interquartile range), mean (standard deviation), or n/N (%).

RYGB = Roux-en-Y gastric bypass, AGB = adjustable gastric band, SG = sleeve gastrectomy, ASA = LDL-cholesterol = low-density lipoprotein cholesterol

Missing data (RYGB, AGB, SG): [1] 35 participants (13, 14, 8), [2]5 participants (3, 1, 1), [3]11 participants (4, 1, 6), [4]7 participants (3,1, 3), [5]79 participants (39, 22, 18), [6]65 participants (26, 23, 16), [7]22 participants (11, 6, 5), [8]29 participants (15, 7, 7), [9]21 participants (11, 5, 5), [10]28 participants (13, 9, 6), [11]22 participants (11, 5, 6), [12]21 participants (11, 5, 5), [13]24 participants (12, 8, 4), [14]19 participants (9, 6, 4), [15]25 participants (12, 7, 6), [16]18 participants (9, 6, 3), [17]35 participants (15, 14, 6), [18]1 participants (26, 29, 16), [19]20 participants (11, 6, 3), [20]24 participants (13, 8, 3), [21]21 participants (11, 6, 4), [22]21 participants (12, 6, 3), [23]117 participants (37, 46, 34), [24]62 participants (27, 24, 11), [25]39 participants (13, 14, 12), [26]36 participants (15, 13, 8), [27]52 participants (21, 19, 12), [83]43 participants (19, 16, 8), [29]73 participants (25, 30, 18), [30]41 participants (19, 16, 6), [31]49 participants (19, 21, 9)

Table 3 Key baseline characteristics of trial participants randomised in the two phases of the trial - intention-to-treat population

	Phase 1			Phase 2			
**Characteristic**	**Randomised to RYGB (n = 109)**	**Randomised to AGB (n = 107)**	**Overall (n = 216)**	**Randomised to RYGB (n = 353)**	**Randomised to AGB (n = 357)**	**Randomised to SG (n = 420)**	**Overall (n = 1130)**
Age[1] (years)	47.8 (10.3)	47.4 (10.0)	47.6 (10.1)	47.3 (10.3)	46.7 (10.5)	47.8 (11.0)	47.3 (10.6)
Female sex	70/109 (64%)	74/107 (69%)	144/216 (67%)	275/352 (78%)	280/357 (78%)	321/419 (77%)	876/1128 (78%)
Ethnicity							
White	107/109 (98%)	101/107 (94%)	208/216(96%)	294/352 (84%)	293/357 (82%)	345/419 (82%)	932/1128 (83%)
Mixed/multiple ethnic groups	2/109 (2%)	2/107 (2%)	4/216(2%)	9/352 (3%)	9/357 (3%)	11/419 (3%)	29/1128 (3%)
Asian/Asian British	0/109 (0%)	1/107 (1%)	1/216(<1%)	10/352 (3%)	20/357 (6%)	15/419 (4%)	45/1128 (4%)
Black/African/Caribbean/Black British	0/109 (0%)	2/107 (2%)	2/216(1%)	30/352 (9%)	24/357 (7%)	36/419 (9%)	90/1128 (8%)
Other ethnic group	0/109 (0%)	1/107 (1%)	1/216(<1%)	9/352 (3%)	11/357 (3%)	12/419 (3%)	32/1128 (3%)
Weight[1] (kg)	136.3 (24.4)	131.8 (23.7)	134.1 (24.1)	129.9 (23.4)	128.2 (22.8)	128.7 (23.8)	128.9 (23.4)
Body mass index[1]	47.1 (7.5)	47.1 (6.9)	47.1 (7.2)	46.8 (7.0)	45.9 (6.5)	46.1 (6.9)	46.2 (6.8)
Diabetic	41/109 (38%)	40/107 (37%)	81/216 (38%)	111/352 (32%)	104/357 (29%)	117/419 (28%)	332/1128 (29%)
Duration of diabetes[2] (years)	3.6 (1.7, 7.1)	4.6 (2.3, 7.8)	4.1 (2.0, 7.1)	3.6 (2.0, 10.1)	5.3 (2.5, 10.0)	4.8 (2.2, 10.3)	4.8 (2.1, 10.0)
Anti-diabetic medication	36/109 (33%)	34/107 (32%)	70/216 (32%)	101/352 (29%)	91/357 (26%)	103/419 (25%)	295/1128 (26%)
Obstructive sleep apnoea	25/109 (23%)	34/107 (32%)	59/216 (27%)	102/351 (29%)	101/357 (28%)	94/418 (22%)	297/1126 (26%)
Gastro-oesophageal reflux disease/ hiatus hernia	51/109 (47%)	60/107 (56%)	111/216 (51%)	158/351 (45%)	169/357 (47%)	212/419 (51%)	539/1127 (48%)
Anti-hypertensive medication	55/99 (56%)	46/105 (44%)	101/204 (50%)	155/325 (48%)	132/324 (41%)	176/379 (46%)	463/1028 (45%)
Anti-hyperlipidaemia medication	31/99 (31%)	33/105 (31%)	64/204 (31%)	100/325 (31%)	94/324 (29%)	110/379 (29%)	304/1028 (30%)

	Phase 1			Phase 2			
Characteristic	Randomised to RYGB (n = 109)	Randomised to AGB (n = 107)	Overall (n = 216)	Randomised to RYGB (n = 353)	Randomised to AGB (n = 357)	Randomised to SG (n = 420)	Overall (n = 1130)
Systolic blood pressure (mmHg)[3]	133 (122, 143)	132 (123, 141)	132 (122, 142)	132 (121, 145)	132 (120, 146)	134 (122, 146)	133 (121, 146)
Diastolic blood pressure (mmHg)[3]	84 (76, 94)	84.0 (78, 91)	84 (77, 93)	83 (76, 89)	81 (74, 88)	82 (75, 89)	82 (75, 88)
HbA1c (mmol/mol)[4]	40 (36, 46)	41 (36, 47)	40 (36, 46)	40 (36, 47)	40 (36, 45)	39 (36, 45)	40 (36, 45)
HbA1c (%)[4]	5.8 (5.4, 6.4)	5.9 (5.4, 6.5)	5.8 (5.4, 6.4)	5.8 (5.4, 6.5)	5.8 (5.4, 6.3)	5.7 (5.4, 6.3)	5.8 (5.4, 6.3)
Total cholesterol (mmol/L)[5]	4.7 (1.3)	4.6 (1.1)	4.6 (1.2)	4.8 (1.1)	4.8 (1.0)	4.9 (1.0)	4.8 (1.0)
HDL-cholesterol (mmol/L)[6]	1.1 (1.0, 1.4)	1.2 (1.0, 1.4)	1.1 (1.0, 1.4)	2.8 (2.2, 3.5)	3.0 (2.3, 3.5)	2.9 (2.3, 3.5)	2.9 (2.3, 3.5)
Triglycerides (mmol/L)[7]	1.4 (1.1, 1.8)	1.5 (1.1, 2.0)	1.4 (1.1, 1.9)	1.5 (1.0, 2.0)	1.4 (1.1, 1.8)	1.4 (1.1, 1.9)	1.4 (1.1, 1.9)
EQ-5D-5L utility score[8]	0.64 (0.31)	0.60 (0.25)	0.62 (0.28)	0.60 (0.28)	0.60 (0.29)	0.61 (0.28)	0.61 (0.28)
EQ-5D-5L visual analogue scale[9]	62.4 (22.1)	62.0 (19.8)	62.2 (21.0)	61.4 (20.7)	61.5 (19.9)	61.3 (22.0)	61.4 (20.9)

Data are presented as median (interquartile range), mean (standard deviation), or n/N (%).

RYGB = Roux-en-Y gastric bypass, AGB = adjustable gastric band, SG = sleeve gastrectomy

Missing data in phase 1 (RYGB, AGB): [3]1 participant (0, 1), [4]10 participants (5,5), [5]8 participants (3,5), [6]8 participants (3, 5), [7]16 participants (8, 8), [8]3 participants (0, 3), [9]4 participants (0, 4)

Missing data in phase 2 (RYGB, AGB, SG): [1]2 participants (1, 0, 1), [2]6 participants (2, 0, 4), [3]16 participants (2, 10, 4), [4]26 participants (10, 9, 7), [5]20 participants (7, 4, 9), [6]24 participants (8, 6, 10), [7]28 participants (11, 8, 9), [8]12 participants (1, 7, 4), [9]15 participants (3, 6, 6)

Table 4 Protocol deviations and crossovers from allocated surgery to another metabolic bariatric surgery - intention-to-treat population

		Randomised to RYGB (n = 462)	Randomised to AGB (n = 464)	Randomised to SG (n = 420)	Overall (n = 1346)
Received surgery		401/462 (87%)	417/464 (90%)	365/420 (87%)	1183/1346 (88%)
Did not receive surgery		61/462 (13%)	47/464 (10%)	55/420 (13%)	163/1346 (12%)
Surgery with 18 weeks of randomisation		185/401 (46%)	222/417 (53%)	173/364 (47%)	580/1183 (49%)
Surgery more than 18 weeks after randomisation		216/401 (54%)	195/417 (47%)	192/365 (53%)	603/1183 (51%)
Received allocated surgery		358/401 (89%)	349/417 (847%)	359/365 (98%)	1066/1183 (90%)
Did not receive allocated surgery		43/401 (11%)	68/417 (16%)	6/365 (2%)	117/1183 (10%)
Surgery abandoned in operating theatre		0/401 (0%)	1/417 (<1%)	1/365 (<1%)	2/1183 (<1%)
**All Crossovers**		**43/401 (11%)**	**67/417 (16%)**	**5/365 (1%)**	**115/1183 (10%)**
**Reasons for crossover**					
Crossed to RYGB	Patient preference		20/67 (30%)	1/5 (20%)	21/115 (18%)
	Clinical reason		4/67 (6%)	1/5 (20%)	5/115 (4%)
	Error		1/67 (1%)	0/5 (0%)	1/115 (1%)
	Unclear		4/67 (6%)	0/5 (0%)	4/115 (3%)
	**Overall**		**29/67 (43%)**	**2/5 (40%)**	**31/115 (27%)**
Crossed to AGB	Patient preference	8/43 (19%)		2/5 (40%)	10/115 (9%)
	Clinical reason	2/43 (5%)		1/5 (20%)	3/115 (3%)
	Error	1/43 (2%)		0/5 (0%)	1/115 (1%)
	**Overall**	**11/43 (26%)**		**3/5 (60%)**	**14/115 (12%)**
Crossed to SG	Patient preference	12/43 (28%)	35/67 (52%)		47/115 (41%)
	Clinical reason	17/43 (40%)	1/67 (1%)		18/115 (16%)
	Error	1/43 (2%)	0/67 (0%)		1/115 (1%)
	Unclear	2/43 (5%)	2/67 (3%)		4/115 (3%)
	**Overall**	**32/43 (74%)**	**38/67 (57%)**		**70/115 (61%)**
**Year of surgery**					
Crossed to RYGB	2013-2015		4/67 (6%)	0/5 (0%)	4/115 (3%)
	2016-2018		18/67 (27%)	1/5 (20%)	19/115 (17%)
	2019-2022		7/67 (10%)	1/5 (20%)	8/115 (7%)
	**Overall**		**29/67 (43%)**	**2/5 (40%)**	**31/115 (27%)**
Crossed to AGB	2013-2015	5/43 (12%)		0/5 (0%)	5/115 (4%)
	2016-2018	2/43 (5%)		2/5 (40%)	4/115 (3%)
	2019-2022	4/43 (9%)		1/5 (20%)	5/115 (4%)
	**Overall**	**11/43 (26%)**		**3/5 (60%)**	**14/115 (12%)**

		Randomised to RYGB (n = 462)	Randomised to AGB (n = 464)	Randomised to SG (n = 420)	Overall (n = 1346)
Crossed to SG	2013-2015	1/43 (2%)	1/67 (1%)		2/115 (2%)
	2016-2018	23/43 (53%)	23/67 (34%)		46/115 (40%)
	2019-2022	8/43 (19%)	14/67 (21%)		22/115 (19%)
	**Overall**	**32/43 (74%)**	**38/67 (57%)**		**70/115 (61%)**

Data are presented as n/N (%)

RYGB = Roux-en-Y gastric bypass, AGB = adjustable gastric band, SG = sleeve gastrectomy

Table 5 Key baseline characteristics of trial participants who received the allocated surgery

Characteristic	Randomised to RYGB (n =358)	Randomised to AGB (n = 349)	Randomised to SG (n = 359)	Overall (n = 1066)
Age (years)	47.0 (10.0)	47.3 (10.2)	47.8 (11.0)	47.4 (10.4)
Female sex	274/358 (77%)	259/349 (74%)	281/359 (78%)	814/1066 (76%)
Ethnicity				
White	324/358 (91%)	306/349 (88%)	303/359 (84%)	933/1066 (88%)
Mixed/multiple ethnic groups	9/358 (3%)	10/349 (3%)	8/359 (2%)	27/1066 (3%)
Asian/Asian British	4/358 (1%)	7/349 (2%)	10/359 (3%)	21/1066 (2%)
Black/African/Caribbean/Black British	15/358 (4%)	20/349 (6%)	29/359 (8%)	64/1066 (6%)
Other ethnic group	6/358 (2%)	6/349 (2%)	9/359 (3%)	21/1066 (2%)
Weight (kg)	130.0 (23.3)	129.6 (23.5)	128.6 (23.5)	129.4 (23.4)
Body mass index	46.6 (7.1)	46.2 (6.6)	46.0 (6.5)	46.3 (6.7)
Diabetic	118/358 (33%)	116/349 (33%)	101/359 (28%)	335/1066 (31%)
Duration of diabetes (years)[1]	3.6 (1.7, 9.1)	5.2 (2.6, 9.4)	4.2 (2.0, 9.5)	4.7 (2.1, 9.3)
Anti-diabetic medication	105/358 (29%)	101/348 (29%)	89/357 (25%)	295/1066 (28%)
Obstructive sleep apnoea	94/357 (26%)	107/349 (31%)	77/358 (22%)	278/1064 (26%)
Gastro-oesophageal reflux disease/ hiatus hernia	160/358 (45%)	172/349 (49%)	178/359 (50%)	510/1066 (48%)
Anti-hypertensive medication	155/358 (43.3%)	143/349 (41.0%)	151/359 (42.1%)	449/1066 (42.1%)
Anti-hyperlipidaemia medication	97/334 (29.0%)	97/324 (29.9%)	92/326 (28.2%)	286/984 (29.1%)
Systolic blood pressure (mmHg)[2]	132 (121, 145)	132 (121, 145)	134 (123, 145)	133 (122, 145)
Diastolic blood pressure (mmHg)[2]	83 (76, 90)	82 (76, 88)	82 (76, 89)	82 (76, 89)
HbA1c (mmol/mol)[3]	39 (36, 47)	40 (36, 48)	40 (36, 45)	40 (36, 46)
HbA1c (%)[3]	5.7 (5.4, 6.5)	5.8 (5.4, 6.5)	5.8 (5.4, 6.3)	5.8 (5.4, 6.4)
Total cholesterol (mmol/L)[4]	4.8 (1.2)	4.7 (1.0)	4.9 (1.0)	4.8 (1.1)
HDL-cholesterol (mmol/L)[5]	1.2 (1.0, 1.4)	1.2 (1.1, 1.4)	1.2 (1.1, 1.5)	1.2 (1.0, 1.4)
Triglycerides (mmol/L)[6]	1.4 (1.1, 2.0)	1.5 (1.1, 1.9)	1.4 (1.0, 1.8)	1.4 (1.1, 1.9)
EQ-5D-5L utility score[7]	0.63 (0.27)	0.60 (0.27)	0.62 (0.27)	0.62 (0.27)
EQ-5D-5L visual analogue scale[8]	62.9 (20.7)	61.9 (19.7)	61.1 (21.8)	62.0 (20.8)

Data are presented as median (interquartile range), mean (standard deviation), or n/N (%).

RYGB = Roux-en-Y gastric bypass, AGB = adjustable gastric band, SG = sleeve gastrectomy

Missing data (RYGB, AGB, SG): [1]6 participants (2, 0, 4), [2]6 participants (0, 4, 2), [3]29 participants (11, 13, 5), [4]22 participants (7, 7, 8), [5]24 participants (8, 7, 9), [6]34 participants (14, 12,8), [7]12 participants (0, 9, 3), [8]16 participants (2, 9, 5)

Table 6 Key baseline characteristics of trial participants who received an alternative surgery

Characteristic	Randomised to RYGB (n = 43)	Randomised to AGB (n = 67)	Randomised to SG (n = 5)	Overall (n = 115)
Age (years)	51.1 (11.3)	43.1 (10.5)	48.0 (13.0)	46.3 (11.5)
Female sex	30/43 (70%)	56/67 (84%)	5/5 (100%)	91/115 (79%)
Ethnicity				
White	33/43 (77%)	55/67 (82%)	3/5 (60%)	91/115 (79%)
Mixed/multiple ethnic groups	2/43 (5%)	0/67 (0%)	1/5 (20%)	3/115 (3%)
Asian/Asian British	1/43 (2%)	8/67 (12%)	1 (20%)	10/115 (9%)
Black/African/Caribbean/Black British	7/43 (16%)	2/67 (3%)	0/5 (0%)	9/115 (8%)
Other ethnic group	0/43 (0%)	2/67 (3%)	0/5 (0%)	2/115 (2%)
Weight (kg)	134.8 (23.0)	132.6 (22.7)	108.2 (19.4)	132.4 (23.1)
Body mass index	47.7 (6.4)	47.6 (6.4)	44.3 (5.8)	47.5 (6.4)
Diabetic	11/43 (26%)	13/67 (19%)	1/5 (20%)	25/115 (22%)
Duration of diabetes (years)	3.8 (1.6, 12.0)	2.4 (1.6, 7.0)	13.7 (13.7, 13.7)	3.4 (1.6, 10.3)
Anti-diabetic medication	10/43 (23%)	9/67 (13%)	1/5 (20%)	20/115 (17%)
Obstructive sleep apnoea	14/43 (33%)	20/67 (30%)	2/5 (40%)	36/115 (31%)
Gastro-oesophageal reflux disease/ hiatus hernia	16/42 (38%)	32/67 (48%)	5 /5(100%)	53/114 (46%)
Anti-hypertensive medication	25/37 (68%)	17/59 (29%)	2/5 (40%)	44/101 (44%)
Anti-hyperlipidaemia medication	16/37 (43%)	12/59 (20%)	1/5 (20%)	29/101 (29%)
Systolic blood pressure (mmHg)[1]	136 (124, 147)	138 (122, 146)	128 (126, 138)	136 (123, 146)
Diastolic blood pressure (mmHg)[1]	86 (79, 90)	82 (74, 88)	78 (70, 82)	83 (75,89)
HbA1c (mmol/mol)[2]	42 (37, 46)	39 (35, 42)	37 (33, 43)	39 (36, 44)
HbA1c (%)[2]	6.0 (5.5, 6.4)	5.7 (5.4, 6.0)	5.5 (5.2, 6.1)	5.7 (5.4, 6.2)
Total cholesterol (mmol/L)[3]	4.8 (1.0)	4.8 (1.0)	4.7 (0.6)	4.8 (1.0)
HDL-cholesterol (mmol/L)[3]	1.2 (1.1, 1.5)	1.2 (1.1, 1.4)	1.2 (1.1, 1.4)	1.2 (1.1, 1.4)
Triglycerides (mmol/L)[3]	1.4 (1.0, 1.9)	1.4 (1.1, 1.9)	2.2 (1.4, 2.9)	1.4 (1.1, 1.9)
EQ-5D-5L utility score[5]	0.55 (0.35)	0.60 (0.30)	0.58 (0.23)	0.58 (0.32)
EQ-5D-5L visual analogue scale[5]	55.8 (20.3)	60.8 (20.1)	63.8 (20.2)	59.1 (20.2)

Data are presented as median (interquartile range), mean (standard deviation), or n/N (%).

RYGB = Roux-en-Y gastric bypass, AGB = adjustable gastric band, SG = sleeve gastrectomy

Missing data (RYGB, AGB, SG): [1]6 participants (0, 6, 0), [2]4 participants (3, 1, 0), [3]3 participants (1, 2, 0), [4]1 participant (1, 0, 0)

Table 7 Key baseline characteristics of trial participants who did not receive surgery within three years of randomisation for any reason

Characteristic	Randomised to RYGB (n = 61)	Randomised to AGB (n = 48[1])	Randomised to SG (n = 56[1])	Overall (n = 165[1])
Age (years)[2]	47.0 (10.8)	48.4 (10.7)	48.1 (11.4)	47.8 (10.9)
Female sex	41/60 (68%)	39/48 (81%)	35/55 (64%)	115/163 (71%)
Ethnicity				
White	44/60 (73%)	33/48 (69%)	39/55 (71%)	116/163 (71%)
Mixed/multiple ethnic groups	0/60 (0%)	1/48 (2%)	2/55 (4%)	3/163 (2%)
Asian/Asian British	5/60 (8%)	6/48 (13%)	4/55 (7%)	15/163 (9%)
Black/African/Caribbean/Black British	8/60 (13%)	4/48 (8%)	7/55 (13%)	19/163 (12%)
Other ethnic group	3/60 (5%)	4/48 (8%)	3/55 (5%)	10/163 (6%)
Weight (kg)[2]	136.9 (26.6)	119.8 (17.9)	131.7 (25.8)	130.1 (24.9)
Body mass index[2]	48.1 (8.0)	43.8 (6.2)	46.5 (9.0)	46.3 (8.0)
Diabetic	23/60 (38%)	15/48 (31%)	15/55 (27%)	53/163 (33%)
Duration of diabetes (years)	3.6 (2.1, 5.4)	4.9 (2.6, 15.3)	9.2 (5.3, 13.1)	5.0 (2.6, 11.0)
Anti-diabetic medication	22/60 (37%)	15/48 (31%)	13/54 (24%)	50/163 (31%)
Obstructive sleep apnoea	19/60 (32%)	8/48 (17%)	15/55 (27%)	42/163 (26%)
Gastro-oesophageal reflux disease/ hiatus hernia	33/60 (55%)	25/48 (52%)	29/55 (53%)	87/163 (53%)
Anti-hypertensive medication	30/53 (57%)	18/46 (39%)	23/48 (48%)	71/147 (48%)
Anti-hyperlipidaemia medication	18/53 (34%)	18/46 (39%)	17/48 (35%)	53/147 (36%)
Systolic blood pressure (mmHg)[3]	131 (122, 142)	128 (114, 142)	135 (122, 152)	132 (121, 145)
Diastolic blood pressure (mmHg)[3]	82 (76, 88)	80 (74, 86)	84 (76, 90)	82 (76, 88)
HbA1c (mmol/mol)[4]	41 (37, 47)	40 (35, 45)	39 (35, 46)	40 (36, 47)
HbA1c (%)[4]	5.9 (5.5, 6.5)	5.8 (5.4, 6.3)	5.7 (5.4, 6.4)	5.8 (5.4, 6.5)
Total cholesterol (mmol/L)[5]	4.9 (1.2)	4.7 (1.0)	4.7 (1.1)	4.8 (1.1)
HDL-cholesterol (mmol/L)[6]	1.2 (1.0, 1.4)	1.2 (1.1, 1.4)	1.2 (1.0, 1.4)	1.2 (1.0, 1.4)
Triglycerides (mmol/L)[7]	1.6 (1.1, 2.1)	1.3 (0.9, 1.7)	1.4 (1.1, 1.9)	1.4 (1.1, 2.0)
EQ-5D-5L utility score[8]	0.53 (0.34)	0.60 (0.31)	0.59 (0.31)	0.57 (0.32)
EQ-5D-5L visual analogue scale[8]	57.9 (22.5)	60.5 (21.2)	62.4 (23.5)	60.2 (22.4)

[1] includes surgeries abandoned in the operating theatre (n=2), one in the AGB group and one in the SG group

Data are presented as median (interquartile range), mean (standard deviation), or n/N (%).

RYGB = Roux-en-Y gastric bypass, AGB = adjustable gastric band, SG = sleeve gastrectomy

Missing data (RYGB, AGB, SG): [2]2 participants (1, 0, 1), [3]5 participants (2, 1, 2), [4]3 participants (1, 0, 2), [5]3 participants (2, 0, 1), [6]5 participants (2, 2, 1), [7]7 participants (4, 2, 1), [8]2 participants (0, 1, 1)

Table 8 Impact of the COVID-19 pandemic on waiting times for surgery and hospital attendance for in-person follow-up - intention-to-treat population

Waiting time (months)	Randomised to Bypass (n=462)	Randomised to Band (n=464)	Randomised to Sleeve (n=420)	Overall (n=1346)
All participants				
Had surgery	401	417	365	1183
Waiting time (months)	5.3 (2.5, 10.3)	4.2 (2.2, 9.2)	5.1 (2.9, 12.1)	5.0 (2.5, 10.1)
Operated before the COVID-19 pandemic				
Had surgery	394	408	356	1158
Waiting time (months)	5.2 (2.5, 10.1)	4.1 (2.2, 8.9)	5.0 (2.9, 11.2)	4.8 (2.5, 9.7)
Operated after the start of the COVID-19 pandemic				
Had surgery	7	9	9	25
Waiting time (months)	27 (19, 33)	31 (17, 32)	22 (17, 29)	27 (17, 31)
Reached three years post-randomisation before the COVID-19 pandemic[1]	228/412 (55%)	215/394 (55%)	167/354 (47%)	610/1160 (53%)
Attended three-year follow-up in person	151/228 (66%)	146/215 (68%)	107/167 (64%)	404/610 (66%)
Completed three-year follow-up by telephone	24/228 (11%)	21/215 (10%)	19/167 (11%)	64/610 (10%)
Other	53/228 (23%)	48/215 (22%)	41/167 (25%)	142/610 (23%)
In follow-up during the COVID-19 pandemic[1]	184/412 (45%)	179/394 (45%)	187/354 (53%)	550/1160 (47%)
Attended three-year follow-up in person	63/184 (34%)	64/179 (36%)	75/187 (40%)	202/550 (37%)
Completed three-year follow-up by telephone	60/184 (33%)	53/179 (30%)	62/187 (33%)	175/550 (32%)
Other	61/184 (33%)	62/179 (35%)	50/187 (27%)	173/550 (31%)

Data are medians and interquartile ranges or number and percentage. Participants who didn't have surgery were censored at last follow-up. Start of the COVID-19 pandemic was taken as the date of the first lockdown – 20 March 2020

[1] excludes participants who withdrew or died within 3 years, or were lost-to-follow-up

Table 9 Surgical details – participants who underwent surgery

Characteristic	Received RYGB (n = 389)	Received AGB (n = 364)	Received SG (n = 430)	Overall (n = 1183)
Surgery abandoned	0/389 (0%)	1/364 (<1%)	1/430 (<1%)	2/1183 (<1%)
Prohibited surgical components carried out	0/389 (0%)	0/363 (0%)	0/429 (0%)	0/1181 (0%)
Mandated surgical components not carried out	12/389 (3%)	13/363 (3%)	7/429 (2%)	31/1181 (3%)
Surgery not carried out laparoscopically	0/389 (0%)	0/362 (0%)	0/429 (0%)	0/1180 (0%)
Prophylactic antibiotics not given	5/389 (1%)	0/362 (0%)	7/429 (2%)	12/1180 (1%)
Inadequate anti DVT prophylaxis given	0/389 (0%)	0/361 (0%)	0/429 (0%)	0/1179 (0%)
Mesenteric defects not closed[1]	7/129 (5%)			
Pars flaccida dissection technique not used		2/361 (1%)		
Gastro-gastric tunnelling sutures not used		4/361 (1%)		
Fat pad not reflected[2]		8/281 (3%)		
Adjustable port not fixed to the anterior abdominal wall		0/360 (0%)		
Left crus not visualised after dissection of fundus			0/426 (0%)	
**Operation and additional procedures**				
Operation length (mins)[3]	132 (105, 171)	70 (55, 88)	90 (65, 120)	94 (67, 129)
Additional procedure(s)	53/369 (14%)	41/344 (12%)	57/429 (13%)	151/1142 (13%)
Hiatus hernia repair	32/53 (60%)	34/41 (83%)	48/57 (84%)	114/151 (75%)
Cholecystectomy	1/53 (2%)	2/41 (5%)	1/57 (1.8%)	4/151 (3%)
Umbilical hernia repair	0/53 (0%)	0/41 (0%)	1/57 (1.8%)	1/151 (1%)
Paraumbilical hernia repair	2/53 (4%)	0/41 (0%)	0/57 (0.0%)	2/151 (1%)
Incisional hernia repair	0/53 (0%)	0/41 (0%)	1/57 (1.8%)	1/151 (1%)
Other	18/53 (34%)	4/41 (10%)	8/57 (14.0%)	30/151 (20%)
**Recovery**				
Time from surgery to discharge (days)[4]	2.0 (2.0, 2.0)	1.0 (0.0, 1.0)	2.0 (2.0, 2.0)	2.0 (1.0, 2.0)
Clavien-Dindo Classification				
Normal recovery (no complications)	358/389 (92%)	347/363 (96%)	394/429 (92%)	1099/1181 (93%)
Grade I	18/389 (5%)	14/363 (4%)	22/429 (5%)	54/1181 (5%)
Grade II	6/389 (2%)	2/363 (1%)	7/429 (2%)	15/1181 (1%)
Grade IIIa	2/389 (0.5%)	0/363 (0%)	2/429 (<1%)	4/1181 (<1%)
Grade IIIb	5/389 (1%)	0/363 (0%)	4/429 (1%)	9/1181 (1%)

Data are presented as n/N (%) or median (interquartile range).

RYGB = Roux-en-Y gastric bypass, AGB = adjustable gastric band, SG = sleeve gastrectomy

[1] Not mandated until April 2018, [2] Denominator excludes where reflection of the fat pad was marked not applicable. Missing data (RYGB, AGB, SG): [3]8 participants (3, 2, 3), [4]1 participants (0, 1, 0)

Table 10 Completeness of outcome data – number of participants included in statistical analyses

Outcome	Overall (n = 1346)
Excess weight loss at 3 years[1]	1344/1346 (99.9%)
Total weight loss	1321/1346 (98.1%)
BMI	1321/1346 (98.1%)
EQ-5D utility score	1284/1346 (95.5%)
EQ-5D visual analogue scale	1283/1346 (95.3%)
SF-12 physical component	1183/1346 (87.9%)
SF-12 mental component	1182/1346 (87.8%)
HADS anxiety score	1185/1346 (88.0%)
HADS depression score	1188/1346 (88.3%)
GIQLI overall score	1195/1346 (88.8%)
GIQLI gastrointestinal score	1196/1346 (88.9%)
IWQOL overall score	1196/1346 (88.9%)
IWQOL self-esteem score	1202/1346 (89.3%)
IWQOL sexual life score	1162/1346 (86.3%)
IWQOL public distress score	1197/1346 (88.9%)
HbA1c	1293/1346 (96.2%)
Fasting glucose	1254/1346 (93.5%)
Triglycerides	1271/1346 (94.4%)
HDL-C	1282/1346 (95.4%)
Total cholesterol	1285/1346 (95.5%)
Ferritin	1278/1346 (95.0%)
Serum iron	1261/1346 (93.7%)
Folate	1275/1346 (94.7%)
Vitamin B12	1278/1346 (95.0%)
25 hydroxyvitamin D	1270/1346 (94.5%)
Parathyroid hormone	1260/1346 (93.6%)
Haemoglobin	1300/1346 (96.6%)
Calcium	1283/1346 (95.3%)
ALT	1296/1346 (96.3%)
ALP	1300/1346 (96.6%)
Creatinine	1303/1346 (96.8%)
Energy intake (kcal/day)[2]	864/1346 (64.2%)
Dietary fibre (g/day)[2]	864/1346 (64.2%)
% energy from protein[2]	860/1346 (63.9%)

Outcome	Overall (n = 1346)
% energy from fat[2]	860/1346 (63.9%)
% energy from carbohydrate[2]	860/1346 (63.9%)
Total folate (µg/day)[2]	864/1346 (64.2%)
Vitamin B1 (mg/day)[2]	861/1346 (64.0%)
Vitamin B12 (mg/day)[2]	831/1346 (61.7%)
Vitamin E (mg/day)[2]	863/1346 (64.1%)
Calcium (mg/day)[2]	864/1346 (64.2%)
Iron (mg/day)[2]	864/1346 (64.2%)
Binge eating score	1144/1346 (85.0%)
Sleepiness	1284/1346 (95.5%)

Data are presented as n/N (%)

[1]2 participants were excluded due to missing outcome data for all time points; data for other participants with missing data at 3-years (n=214) were imputed using multiple imputation

[2] Participants with missing baseline data were excluded from dietary analyses

Participants had to have provided data for at least one post-randomisation time-point to be included in the analysis

Table 11 Primary outcome – at least 50% excess weight loss at 3-years – intention to treat and per-protocol populations

Achieved at least 50% EWL[1]	Randomised to RYGB	Randomised to AGB	Randomised to SG	RYGB minus AGB RD (98% CI)	SG minus AGB RD (98% CI)	SG minus RYGB RD (98% CI)
Primary analysis[2]	276/405 (68%)	97/383 (25%)	141/342 (41%)	0.407 (0.339, 0.475)	0.147 (0.052, 0.242)	-0.260 (-0.358, -0.163)
**Sensitivity analyses**						
Adjusting for design phase[2,3]	276/405 (68%)	97/383 (25%)	141/342 (41%)	0.410 (0.341, 0.478)	0.144 (0.031, 0.258)	-0.265 (-0.381, -0.15)
Excluding participants recruited during phase 1[2,3]	206/304 (68%)	78/293 (27%)	141/342 (41%)	0.387 (0.312, 0.463)	0.134 (0.024, 0.244)	-0.253 (-0.374, -0.133)
Excluding participants with missing outcome	229/338 (77%)	84/325 (21%)	123/290 (44%)	0.423 (0.364, 0.481)	0.158 (0.064, 0.252)	-0.265 (-0.365, -0.165)
Excluding crossovers	258/363 (71%)	62/328 (19%)	137/336 (41%)	0.490 (0.427, 0.553)	0.201 (0.099, 0.302)	-0.289 (-0.385, -0.194)
Excluding participants who didn't have surgery	274/375 (73%)	96/363 (26%)	140/314 (45%)	0.457 (0.395, 0.519)	0.175 (0.077, 0.273)	-0.282 (-0.384, -0.181)
Excluding crossovers and participants who didn't have surgery[2,4]	256/333 (77%)	61/308 (20%)	136/309 (44%)	0.561 (0.482, 0.64)	0.231 (0.126, 0.336)	-0.330 (-0.438, -0.222)
Excluding crossovers and participants who didn't have surgery, adjusting for design phase[2,3,4]	256/333 (77%)	61/308 (20%)	136/309 (44%)	0.560 (0.480, 0.640)	0.224 (0.093, 0.354)	-0.337 (-0.457, -0.217)
Excluding crossovers, participants who didn't have surgery and participants recruited during phase 1[2,3,4]	189/242 (78%)	46/222 (21%)	136/309 (44%)	0.558 (0.464, 0.652)	0.221 (0.100, 0.342)	-0.337 (-0.473, -0.200)

	Received RYGB	Received AGB	Received SG	RYGB minus AGB RD (98% CI)	SG minus AGB RD (98% CI)	SG minus RYGB RD (98% CI)
Excluding participants who didn't have surgery	276/356 (77.5%)	67/322 (20.8%)	167/374 (44.7%)	0.556 (0.472, 0.639)	0.227 (0.110, 0.345)	-0.328(-0.433, -0.223)
	**Randomised to RYGB**	**Randomised to AGB**	**Randomised to SG**	**RYGB minus AGB RD (98% CI)**	**SG minus AGB RD (98% CI)**	**SG minus RYGB RD (98% CI)**
**Subgroup analyses**						
Non-diabetic	197/270 (73%)	75/264 (28%)	112/246 (46%)	0.414 (0.332, 0.496)	0.151 (0.035, 0.267)	-0.263 (-0.361, -0.165)
Diabetic	79/135 (59%)	22/119 (18%)	29/96 (30%)	0.395 (0.306, 0.483)	0.141 (0.012, 0.270)	-0.254 (-0.411, -0.096)
Test for treatment by subgroup interaction p=0.90						
BMI<40	47/60 (78%)	19/72 (26%)	26/66 (39%)	0.496 (0.291, 0.701)	0.138 (0.009, 0.268)	-0.357 (-0.599, -0.116)
BMI 40 to <50	169/238 (71%)	55/208 (26%)	79/193 (41%)	0.441 (0.333, 0.549)	0.144 (0.027, 0.26)	-0.297 (-0.428, -0.166)
BMI >50	60/107 (56%)	23/103 (22%)	36/83 (43%)	0.300 (0.164, 0.437)	0.164 (0.015, 0.313)	-0.137 (-0.289, 0.016)
Test for treatment by subgroup interaction p=0.30						

Data are presented as n/N (%) or treatment differences and 98% confidence intervals and apply to the intention to treat population unless indicated otherwise

[1]Multiple imputation (50 imputed datasets) was used to account for missing outcome in analyses, counts exclude imputed observations. Models could not be adjusted for site.

[2] Post hoc analyses

[3] Design phase 1 – allocation to one of two groups (RYGB or AGB), design phase 2 allocation to one of three groups (RYGB, AGB or SG)

[4] Per protocol population

Differences of less than -12% (i.e. with the lower limit of the confidence interval above -0.12) are considered non-inferior

RYGB = Roux-en-Y gastric bypass, AGB = adjustable gastric band, SG = sleeve gastrectomy, RD = risk difference, CI = confidence interval

Table 12 Primary outcome – EQ-5D utility score – intention to treat and per-protocol populations

EQ-5D at 3 years	Randomised to RYGB mean (SD)	Randomised to AGB mean (SD)	Randomised to SG mean (SD)	RYGB minus AGB MD (98% CI)	SG minus AGB MD (98% CI)	SG minus RYGB MD (98% CI)
Primary analysis	0.718 (0.288)	0.619 (0.330)	0.676 (0.297)	0.079 (0.040, 0.117)	0.045 (0.006, 0.085)	-0.033 (-0.072, 0.006)
**Sensitivity analyses**						
Adjusting for design phase[1,2]	0.718 (0.288)	0.619 (0.330)	0.676 (0.297)	0.079 (0.04, 0.117)	0.052 (0.011, 0.092)	-0.027 (-0.067, 0.013)
Excluding participants recruited during phase 1[1,2]	0.705 (0.293)	0.610 (0.334)	0.676 (0.297)	0.072 (0.028, 0.116)	0.048 (0.006, 0.09)	-0.024 (-0.066, 0.018)
Excluding participants with missing outcome	0.721 (0.287)	0.617 (0.330)	0.673 (0.300)	0.082 (0.040, 0.123)	0.043 (-0.00, 0.086)	-0.039 (-0.081, 0.004)
Excluding crossovers	0.725 (0.279)	0.606 (0.333)	0.677 (0.297)	0.093 (0.051, 0.134)	0.060 (0.018, 0.101)	-0.033 (-0.073, 0.008)
Excluding participants who didn't have surgery	0.734(0.280)	0.628 (0.322)	0.677 (0.295)	0.089 (0.050, 0.129)	0.046 (0.006, 0.086)	-0.043 (-0.084, -0.003)
Excluding crossovers and participants who didn't have surgery[1,3]	0.744 (0.269)	0.615 (0.324)	0.677 (0.296)	0.105 (0.062, 0.147)	0.06 (0.017, 0.102)	-0.045 (-0.087, -0.003)
Excluding crossovers and participants who didn't have surgery, adjusting for design phase[1,2,3]	0.744 (0.269)	0.615 (0.324)	0.677 (0.296)	0.105 (0.063, 0.148)	0.067 (0.024, 0.11)	-0.038 (-0.081, 0.004)
Excluding crossovers, participants who didn't have surgery and participants recruited during phase 1[1,2,3]	0.727 (0.277)	0.606 (0.327)	0.677 (0.296)	0.095 (0.046, 0.144)	0.063 (0.017, 0.109)	-0.033 (-0.077, 0.012)
Three years from surgery	0.734 (0.280)	0.628 (0.322)	0.677 (0.295)	0.067 (0.022, 0.112)	0.030 (-0.016, 0.076)	-0.037 (-0.083, 0.009)

	Received RYGB	Received AGB	Received SG	RYGB minus AGB MD (98% CI)	SG minus AGB MD (98% CI)	SG minus RYGB MD (98% CI)
Excluding participants who didn't have surgery	0.741 (0.274)	0.617 (0.324)	0.678 (0.299)	0.104 (0.063, 0.145)	0.061 (0.021, 0.101)	-0.043 (-0.082, -0.004)
	**Randomised to RYGB**	**Randomised to AGB**	**Randomised to SG**	**RYGB minus AGB MD (98% CI)**	**SG minus AGB MD (98% CI)**	**SG minus RYGB MD (98% CI)**
**Subgroup analyses**						
Non-diabetic	0.716 (0.303)	0.603 (0.348)	0.675 (0.303)	0.082 (0.039, 0.125)	0.050 (0.007, 0.094)	-0.031 (-0.074, 0.011)
Diabetic	0.722 (0.256)	0.656 (0.284)	0.678 (0.281)	0.072 (0.017, 0.126)	0.033 (-0.025, 0.091)	-0.038 (-0.095, 0.019)
Test for treatment by subgroup interaction p=0.79						
BMI<40	0.741 (0.291)	0.659 (0.341)	0.675 (0.285)	0.061 (-0.010, 0.133)	0.004 (-0.064, 0.073)	-0.057 (-0.129, 0.015)
BMI 40 to <50	0.729 (0.289)	0.627 (0.326)	0.697 (0.275)	0.072 (0.027, 0.117)	0.055 (0.008, 0.102)	-0.017 (-0.062, 0.029)
BMI >50	0.684 (0.284)	0.575 (0.331)	0.629 (0.347)	0.103 (0.045, 0.160)	0.055 (-0.005, 0.114)	-0.048 (-0.107, 0.011)
Test for treatment by subgroup interaction p=0.31						

Data are presented as mean (SD) or treatment differences and 98% confidence intervals and apply to the intention to treat population unless indicated otherwise

[1] Post hoc analyses

[2] Phase 1 – allocation to one of two groups (RYGB or AGB), phase 2 allocation to one of three groups (RYGB, AGB or SG)

[3] Per protocol population

RYGB = Roux-en-Y gastric bypass, AGB = adjustable gastric band, SG = sleeve gastrectomy, SD = standard deviation, MD = mean difference, CI = confidence interval

Figure 1 Proportion achieving 50% excess weight loss over time by randomised group

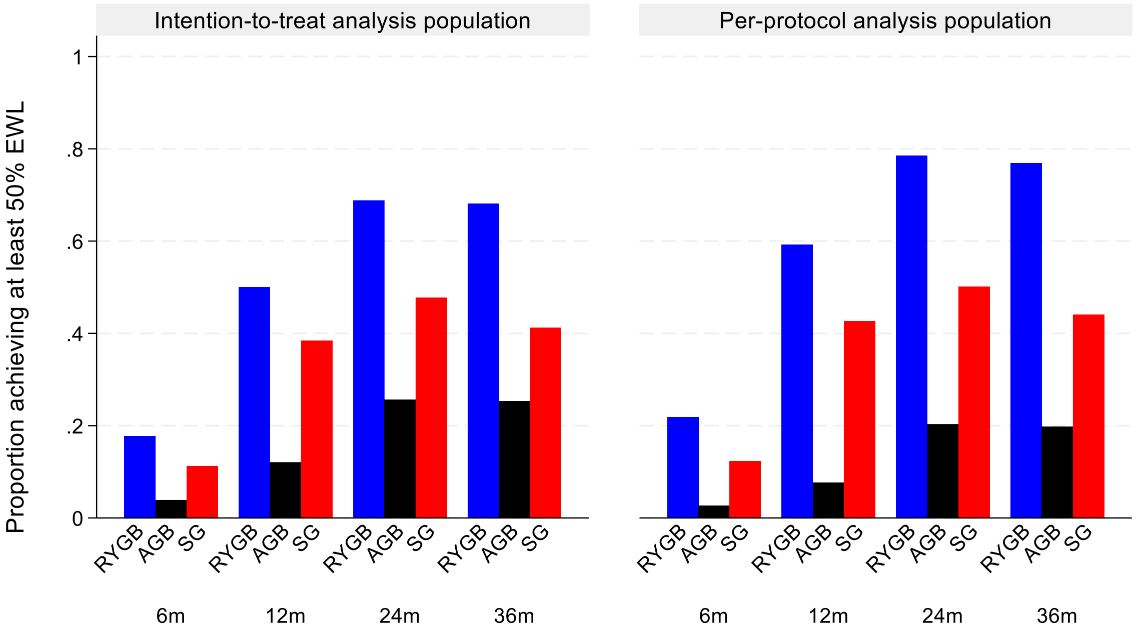

Data are based on reported weight at each follow-up

RYGB = Roux-en-Y gastric bypass, AGB = adjustable gastric band, SG = sleeve gastrectomy, EWL = excess weight lost

Table 13 Percentage total weight loss at 3-years - intention-to-treat population

% total weight loss (TWL) at 3-years	Randomised to RYGB (n = 462)	Randomised to AGB (n = 464)	Randomised to SG (n = 420)	Overall (n = 1346)
At least 10% TWL	364/405 (90%)	217/383 (57%)	269/342 (79%)	850/1130 (75%)
At least 15% TWL	345/405 (85%)	166/383 (43%)	217/342 (63%)	728/1130 (64%)
At least 20% TWL	296/405 (73%)	122/383 (32%)	168/342 (49%)	586/1130 (52%)

Data are presented as n/N (%)

TWL = total weight loss, RYGB = Roux-en-Y gastric bypass, AGB = adjustable gastric band, SG = sleeve gastrectomy

Table 14 Waist circumference at baseline and 3-years - intention-to-treat population

	Randomised to RYGB (n=462)	Randomised to AGB (n=464)	Randomised to SG (n=420)	Overall (n=1346)
Circumference (cm)				
Baseline[1]	130.9 (14.9)	130.6 (14.6)	129.8 (15.1)	130.5 (14.8)
3 years[2]	104.4 (17.1)	116.4 (18.2)	109.9 (17.1)	110.2 (18.2)

Data are presented as mean (standard deviation)

RYGB = Roux-en-Y gastric bypass, AGB = adjustable gastric band, SG = sleeve gastrectomy

Missing data (RYGB, AGB, SG): [1]37 participants (14, 10, 13), [2]785 participants (263, 270, 252)

Figure 2 Weight loss over time by randomised group - intention-to-treat population

a) Body mass index (kg/m^2)

b) Percentage total weight loss

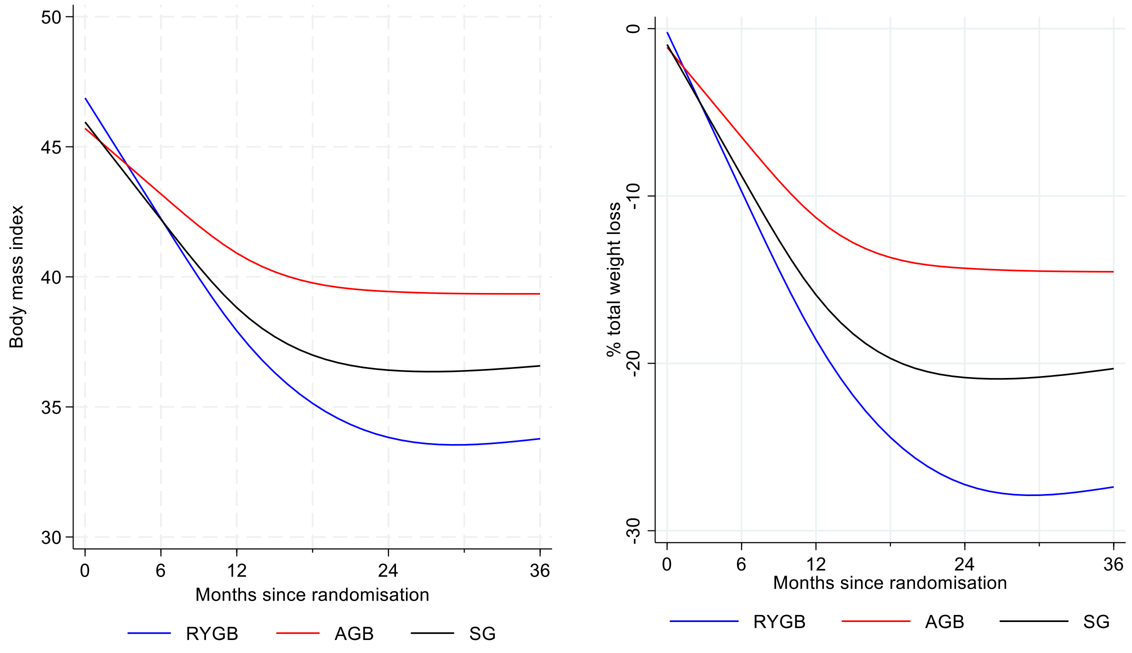

Trajectories are estimated from a regression model and represent the predicted mean values

RYGB = Roux-en-Y gastric bypass, AGB = adjustable gastric band, SG = sleeve gastrectomy

Note: scale of the y-axis differs between the two graphs

Table 15 HbA1c and antidiabetic medication at baseline and 3-years - intention-to-treat population

	Randomised to RYGB (n = 462)	Randomised to AGB (n = 464)	Randomised to SG (n = 420)	Overall (n = 1346)
**All participants**				
HbA1c < 48 mmol/mol				
Baseline	343/447 (77%)	350/450 (78%)	328/413 (79%)	1021/1310 (78%)
3 years	293/323 (91%)	253/308 (82%)	233/266 (88%)	779/897 (87%)
HbA1c < 48 mmol/mol and not taking antidiabetic medication				
Baseline	259/412 (63%)	274/415 (66%)	259/373 (69%)	792/1200 (66%)
3 years	244/291 (84%)	200/268 (75%)	190/230 (83%)	634/789 (80%)
**Participants without diabetes at baseline**				
HbA1c < 48 mmol/mol				
Baseline	290/300 (97%)	300/309 (97%)	288/298 (97%)	878/907 (97%)
3 years	203/205 (99%)	202/205 (99%)	183/185 (99%)	588/595 (99%)
HbA1c < 48 mmol/mol and not taking antidiabetic medication				
Baseline	248/266 (93%)	258/275 (94%)	249/260 (96%)	755/801 (94%)
3 years	180/184 (98%)	169/180 (94%)	157/158 (99%)	506/522 (97%)
**Participants with diabetes at baseline**				
HbA1c < 48 mmol/mol				
Baseline	53/147 (36%)	50/141 (35%)	40/115 (35%)	143/403 (35%)
3 years	90/118 (76%)	51/103 (50%)	50/81 (62%)	191/302 (63%)
HbA1c < 48 mmol/mol and not taking antidiabetic medication				
Baseline	11/146 (8%)	16/140 (11%)	10/113 (9%)	37/399 (9%)
3 years	64/107 (60%)	31/88 (35%)	33/72 (46%)	128/267 (48%)

Data are presented as n/N (%)

RYGB = Roux-en-Y gastric bypass, AGB = adjustable gastric band, SG = sleeve gastrectomy

Note: the diabetes sub-group includes participants with glucose impairment, pre-diabetes, diet- controlled diabetes and medically controlled diabetes

Table 16 Blood pressure, anti-hypertensive medication, total cholesterol and anti-hyperlipidaemic medication at baseline and 3-years - intention-to-treat population

	Randomised to RYGB (n=462)	Randomised to AGB (n=464)	Randomised to SG (n=420)	Overall (n=1346)
Systolic BP < 130 mmHg and diastolic BP < 85 mmHg				
Baseline	104/422 (25%)	126/417 (30%)	98/384 (25.5%)	328/1223 (27%)
3 years	97/225 (43%)	76/202 (38%)	72/173 (42%)	245/600 (41%)
On anti-hypertensives				
Baseline	210/424 (50%)	178/429 (41%)	176/379 (46%)	564/1232 (46%)
3 years	107/350 (31%)	117/323 (36%)	108/297 (36%)	332/970 (34%)
Total cholesterol ≤ 5mmol				
Baseline	266/452 (59%)	269/455 (59%)	226/411 (55%)	761/1318 (58%)
3 years	215/293 (73%)	166/284 (58%)	129/249 (52%)	510/826 (62%)
On anti-hyperlipidaemic medication				
Baseline	131/424 (31%)	127/429 (30%)	110/379 (29%)	368/1232 (30%)
3 years	78/350 (22%)	76/323 (24%)	73/297 (25%)	227/970 (23%)

Data are presented as n/N (%)

BP = blood pressure, RYGB = Roux-en-Y gastric bypass, AGB = adjustable gastric band, SG = sleeve gastrectomy

Table 17 Vitamin D at baseline and 3-years - intention-to-treat population

	Randomised to RYGB (n=462)	Randomised to AGB (n=464)	Randomised to SG (n=420)	Overall (n=1346)
Vitamin D ≤ 50 nmol/L				
Baseline	272/443 (61%)	273/448 (61%)	250/412 (61%)	795/1303 (61%)
3 years	76/285 (27%)	106/256 (41%)	70/238 (29%)	252/779 (32%)

Data are presented as n/N (%)

RYGB = Roux-en-Y gastric bypass, AGB = adjustable gastric band, SG = sleeve gastrectomy

Figure 3 Outcomes measured in blood over time by randomised group - intention-to-treat population

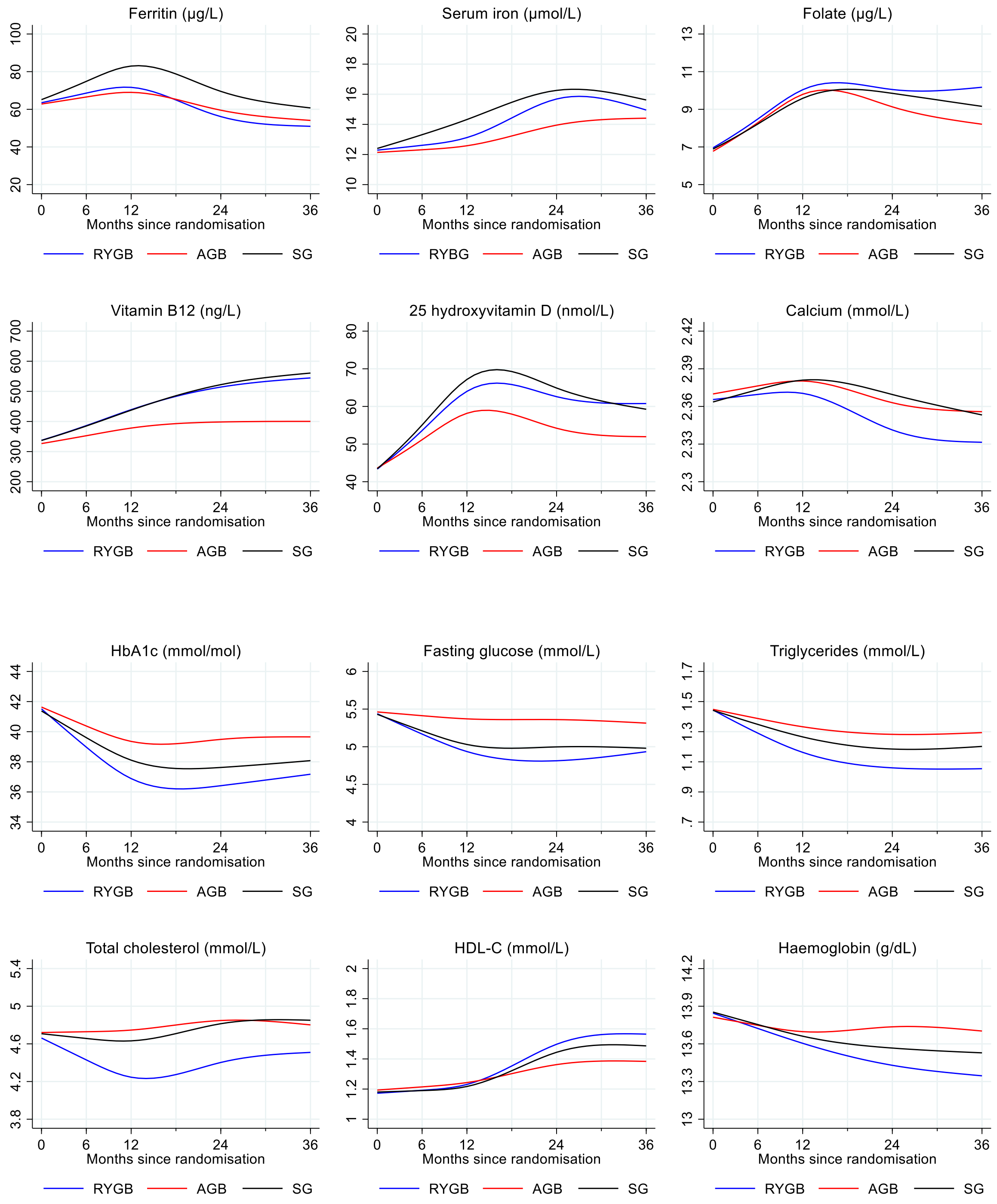

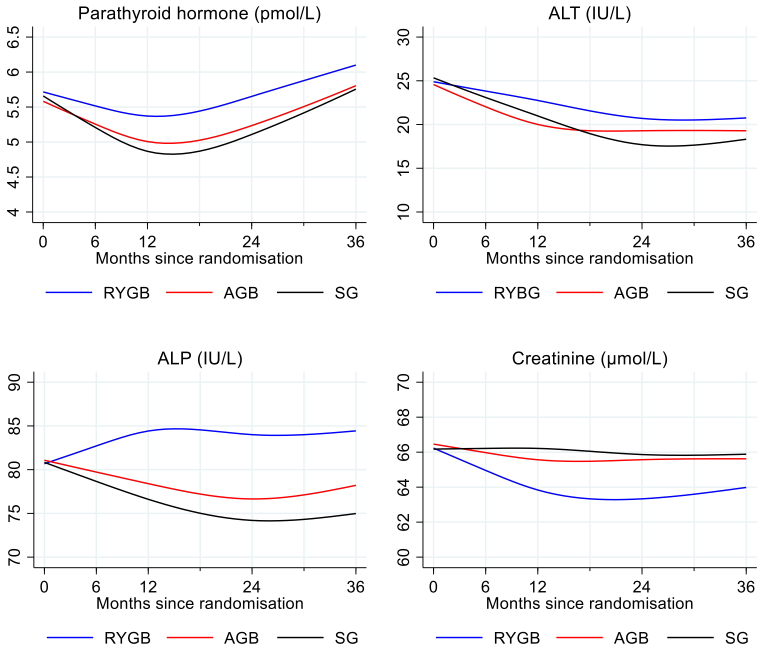

Trajectories are estimated from a regression model and represent the predicted mean values

HbA1c = glycated haemoglobin, HDL- C = high-density lipoprotein cholesterol, ALP = alkaline phosphatase, ALT = alanine transaminase, RYGB = Roux-en-Y gastric bypass, AGB = adjustable gastric band, SG = sleeve gastrectomy

Note: the scale on the y-axis differs across the blood measures

Table 18 Other markers measured in blood at baseline and 3-years - intention-to-treat population

	Randomised to RYGB (n=462)	Randomised to AGB (n=464)	Randomised to SG (n=420)	Overall (n=1346)
Haematocrit (L/L)				
Baseline[1]	0.42 (0.40, 0.45)	0.42 (0.40, 0.44)	0.42 (0.40, 0.44)	0.42 (0.40, 0.44)
3 years[2]	0.41 (0.39, 0.43)	0.41 (0.39, 0.43)	0.41 (0.39, 0.44)	0.41 (0.39, 0.43)
Mean cell haemoglobin (pg)				
Baseline[3]	28.8 (2.1)	28.8 (2.0)	29.0 (4.6)	28.9 (3.1)
3 years[4]	30.0 (3.7)	29.4 (2.0)	29.6 (2.5)	29.7 (2.9)
Mean cell volume (fL)				
Baseline[5]	88.4 (84.9, 91.3)	88.0 (84.6, 91.2)	88.1 (84.3, 91.5)	88.2 (84.6, 91.3)
3 years[6]	91.1 (87.8, 94.2)	89.2 (86.0, 92.3)	90.5 (87.4, 93.5)	90.4 (87.0, 93.7)
Albumin (g/L)				
Baseline[7]	40.4 (3.8)	40.3 (3.8)	40.0 (3.8)	40.2 (3.8)
3 years[8]	39.7 (3.5)	40.1 (3.8)	39.6 (3.5)	39.8 (3.6)
Bilirubin (µmol/L)				
Baseline[9]	9.5 (4.7)	9.5 (4.7)	9.8 (4.4)	9.6 (4.6)
3 years[10]	10.7 (5.8)	10.2 (5.9)	10.5 (5.9)	10.5 (5.8)
Total protein (g/L)				
Baseline[11]	72 (69, 74)	72 (69, 75)	72 (69, 75)	72 (69, 75)
3 years[12]	68 (65, 71)	70 (67, 73)	70 (67, 73)	69 (67, 72)
LDL-cholesterol (mmol/L)				
Baseline[13]	2.8 (2.2, 3.4)	2.9 (2.2, 3.5)	2.9 (2.3, 3.5)	2.9 (2.3, 3.5)
3 years[14]	2.3 (1.9, 2.9)	2.8 (2.2, 3.4)	3.0 (2.2, 3.5)	2.6 (2.0, 3.3)
Phosphate (mmol/L)				
Baseline[15]	1.1 (1.0, 1.2)	1.1 (1.0, 1.2)	1.1 (0.9, 1.2)	1.1 (1.0, 1.2)
3 years[16]	1.1 (1.0, 1.2)	1.1 (1.0, 1.2)	1.1 (1.0, 1.2)	1.1 (1.0, 1.2)
Potassium (mmol/L)				
Baseline[17]	4.3 (4.1, 4.5)	4.3 (4.1, 4.5)	4.3 (4.1, 4.5)	4.3 (4.1, 4.5)
3 years[18]	4.3 (4.0, 4.5)	4.3 (4.1, 4.5)	4.3 (4.1, 4.5)	4.3 (4.1, 4.5)
Sodium (mmol/L)				
Baseline[19]	139 (138, 141)	139 (138, 141)	140 (138, 141)	139 (138, 141)
3 years[20]	140 (139, 142)	140 (139, 141)	140 (139, 141)	140 (139, 141)
Urea (mmol/L)				
Baseline[21]	4.9 (3.9, 5.8)	4.6 (3.9, 5.7)	4.7 (4.0, 5.7)	4.8 (3.9, 5.8)
3 years[22]	4.5 (3.8, 5.5)	4.5 (3.6, 5.6)	4.7 (3.9, 5.7)	4.6 (3.8, 5.6)

Data are presented as mean (standard deviation) or median (interquartile range)

LDL-cholesterol = Low-density lipoprotein cholesterol, RYGB = Roux-en-Y gastric bypass, AGB = adjustable gastric band, SG = sleeve gastrectomy

Missing data (RYGB, AGB, SG): [1]29 participants (15, 7, 7), [2]382 participants (110, 137, 135), [3]22 participants (11, 6, 5), [4]372 participants (110, 132, 130), [5]22 participants (11, 6, 5), [6]369 participants (109, 132, 128), [7]18 participants (9, 6, 3), [8]367 participants (109, 135, 123), [9]25 participants (12, 7, 6), [10]385 participants (113, 143, 129), [11]35 participants (15, 14, 6), [12]489 participants (149, 178, 162), [13]65 participants (26, 23, 16), [14]585 participants (188, 204, 193), [15]49 participants (19, 21, 9), [16]586 participants (177, 215, 194), [17]24 participants (13, 8, 3), [18]348 participants (100, 125, 123), [19]20 participants (11, 6, 3), [20]344 participants (99, 124, 121), [21]21 participants (11, 6, 4), [22]399 participants (121, 145, 133)

Table 19 Outcomes related to diet at 3 years - intention-to-treat population

	Randomised to RYGB<br>Geometric mean (CV)	Randomised to AGB<br>Geometric mean (CV)	Randomised to SG<br>Geometric mean (CV)	RYGB minus AGB<br>GMR (98% CI)	SG minus AGB<br>GMR (98% CI)	SG minus RYGB<br>GMR (98% CI)
Energy intake (kcal/day)	1069 (0.6)	971 (0.5)	1004 (0.5)	1.06 (0.92, 1.22)	1.09 (0.92, 1.28)	1.03 (0.87, 1.20)
Dietary fibre (g/day)	9.4 (0.6)	7.9 (0.9)	9.4 (0.8)	1.14 (0.94, 1.39)	1.14 (0.91, 1.44)	1.00 (0.80, 1.25)
% energy from protein	18.0 (0.4)	18.5 (0.4)	17.2 (0.4)	0.97 (0.88, 1.07)	0.93 (0.83, 1.05)	0.96 (0.86, 1.07)
Total folate (µg/day)	128.8 (0.7)	123.9 (0.8)	128.6 (1)	0.97 (0.79, 1.19)	1.12 (0.88, 1.43)	1.15 (0.92, 1.46)
Vitamin B1 (mg/day)	0.8 (1)	0.7 (0.9)	0.8 (0.9)	1.02 (0.82, 1.27)	1.24 (0.96, 1.60)	1.21 (0.95, 1.55)
Vitamin B12 (mg/day)	2.2 (1.3)	2.0 (1.1)	2.2 (1.2)	1.21 (0.91, 1.61)	1.25 (0.89, 1.76)	1.03 (0.74, 1.43)
Vitamin E (mg/day)	2.8 (1.2)	2.4 (1.3)	3.6 (1.5)	1.19 (0.90, 1.57)	1.56 (1.12, 2.17)	1.31 (0.95, 1.80)
Calcium (mg/day)	492 (0.7)	469 (0.7)	478 (0.6)	0.97 (0.81, 1.16)	1.03 (0.84, 1.28)	1.06 (0.87, 1.30)
Iron (mg/day)	5.5 (0.7)	4.9 (0.7)	5.7 (0.7)	1.06 (0.88, 1.28)	1.20 (0.96, 1.49)	1.13 (0.91, 1.39)
	**mean (SD)**	**mean (SD)**	**mean (SD)**	**MD (98% CI)**	**MD (98% CI)**	**MD (98% CI)**
% energy from fat	33.6 (11.4)	32.6 (10.5)	34.6 (10.5)	1.60 (-1.59, 4.78)	2.03 (-1.73, 5.80)	0.44 (-3.17, 4.05)
% energy from carbohydrate	46.6 (13.4)	48.4 (13.9)	45.9 (13.0)	-1.98 (-5.71, 1.75)	-2.00 (-6.40, 2.40)	-0.01 (-4.23, 4.20)
Binge eating score	10.83 (8.87)	12.56 (8.61)	11.09 (8.47)	-2.78 (-4.48, -1.08)	-1.78 (-3.54, -0.02)	1.00 (-0.75, 2.74)

Data are presented as mean (SD), geometric mean (CV) or treatment differences and 98% confidence intervals

RYGB = Roux-en-Y gastric bypass, AGB = adjustable gastric band, SG = sleeve gastrectomy, CV = coefficient of variation, SD = standard deviation, GMR = geometric mean ratio (RYGB/AGB, SG/AGB, SG/RYGB), MD = mean difference, CI = confidence interval

Table 20 Liver fibrosis at 3 years - intention-to-treat population

Liver fibrosis	Randomised to RYGB	Randomised to AGB	Randomised to SG	RYGB minus AGB Odds ratio (98% CI)	SG minus AGB Odds ratio (98% CI)	SG minus RYGB Odds ratio (98% CI)
None/mild (Ishak<3)	3/111 (3%)	3/104 (3%)	2/76 (3%)	1.26 (0.54, 2.92)	1.44 (0.51, 4.08)	1.14 (0.47, 2.79)
Moderate (Ishak 3-4)	86/111 (77%)	84/104 (81%)	60/76 (79%)			
Severe (Ishak>4)	22/111 (20%)	17/104 (16%)	14/76 (18%)			

Data are presented as n/N (%) or treatment effects and 98% confidence intervals

RYGB = Roux-en-Y gastric bypass, AGB = adjustable gastric band, SG = sleeve gastrectomy, CI = confidence interval

Table 21 Epworth sleepiness scale at 3 years - intention-to-treat population

Sleepiness	Randomised to RYGB	Randomised to AGB	Randomised to SG	RYGB minus AGB MD (98% CI)	SG minus AGB MD (98% CI)	SG minus RYGB MD (98% CI)
Sleepiness	3.58 (3.77)	4.26 (4.23)	4.06 (4.24)	-1.02 (-1.66, -0.38)	-0.68 (-1.34, -0.02)	0.34 (-0.31, 1.00)

Data are presented as mean (standard deviation) or treatment differences and 98% confidence intervals

Scale ranges from 0 to 24, higher scores indicate more sleepiness.

RYGB = Roux-en-Y gastric bypass, AGB = adjustable gastric band, SG = sleeve gastrectomy, MD = mean difference, CI = confidence interval

Table 22 Prevalence of sleep apnoea at baseline and 3 years - intention-to-treat population

	Randomised to RYGB (n=462)	Randomised to AGB (n=464)	Randomised to SG (n=420)	Overall (n=1346)
**On CPAP/BIPAP**				
Baseline	107/460 (23%)	117/464 (25%)	82/418 (20%)	306/1342 (23%)
3 years	37/303 (12%)	50/287 (17%)	36/267 (13%)	123/857 (14%)
**Obstructive sleep apnoea diagnosis, but unable to tolerate CPAP/BIPAP**				
Baseline	17/460 (4%)	15/464 (3%)	10/418 (2%)	42/1342 (3%)
3 years	6/303 (2%)	13/287 (5%)	11/267 (4%)	30/857 (4%)

Data are presented as n/N (%)

RYGB = Roux-en-Y gastric bypass, AGB = adjustable gastric band, SG = sleeve gastrectomy, CPAP/BiPAP = continuous/bilevel positive airway pressure

Table 23 Medication and dietary supplements taken during follow-up - intention-to-treat population

	Randomised to RYGB (n=462)	Randomised to AGB (n=464)	Randomised to SG (n=420)	Overall (n=1346)
On asthma medication				
Baseline	94/424 (22%)	114/429 (27%)	107/379 (28%)	315/1232 (26%)
3 years	48/350 (14%)	67/323 (21%)	57/297 (19%)	172/970 (18%)
On GORD medication				
Baseline	158/424 (37%)	187/429 (44%)	143/379 (38%)	488/1232 (40%)
3 years	125/350 (36%)	120/323 (37%)	137/297 (46%)	382/970 (39%)
On anti-coagulants				
Baseline	8/424 (2%)	9/429 (2%)	9/379 (2%)	26/1232 (2%)
3 years	11/350 (3%)	9/323 (3%)	12/297 (4%)	32/970 (3%)
On anti-depressants				
Baseline	165/424 (39%)	190/429 (44%)	153/379 (40%)	508/1232 (41%)
3 years	130/350 (37%)	129/323 (40%)	105/297 (35%)	364/970 (38%)
On analgesia medication				
Baseline	216/424 (51%)	219/429 (51%)	204/379 (54%)	639/1232 (52%)
3 years	139/350 (40%)	144/323 (45%)	125/297 (42%)	408/970 (42%)
Other medication				
Baseline	254/424 (60%)	263/429 (61%)	234/379 (62%)	751/1232 (61%)
3 years	184/350 (53%)	188/323 (58%)	175/297 (59%)	547/970 (56%)
Dietary supplements				
Baseline	179/424 (42%)	165/429 (38%)	142/379 (37%)	486/1232 (39%)
3 years	318/350 (91%)	249/323 (77%)	266/297 (90%)	833/970 (86%)

Data are presented as n/N (%)

RYGB = Roux-en-Y gastric bypass, AGB = adjustable gastric band, SG = sleeve gastrectomy, GORD = gastro-oesophageal reflux disease

Figure 4 Trajectories of generic quality of life outcomes over time - intention-to-treat population

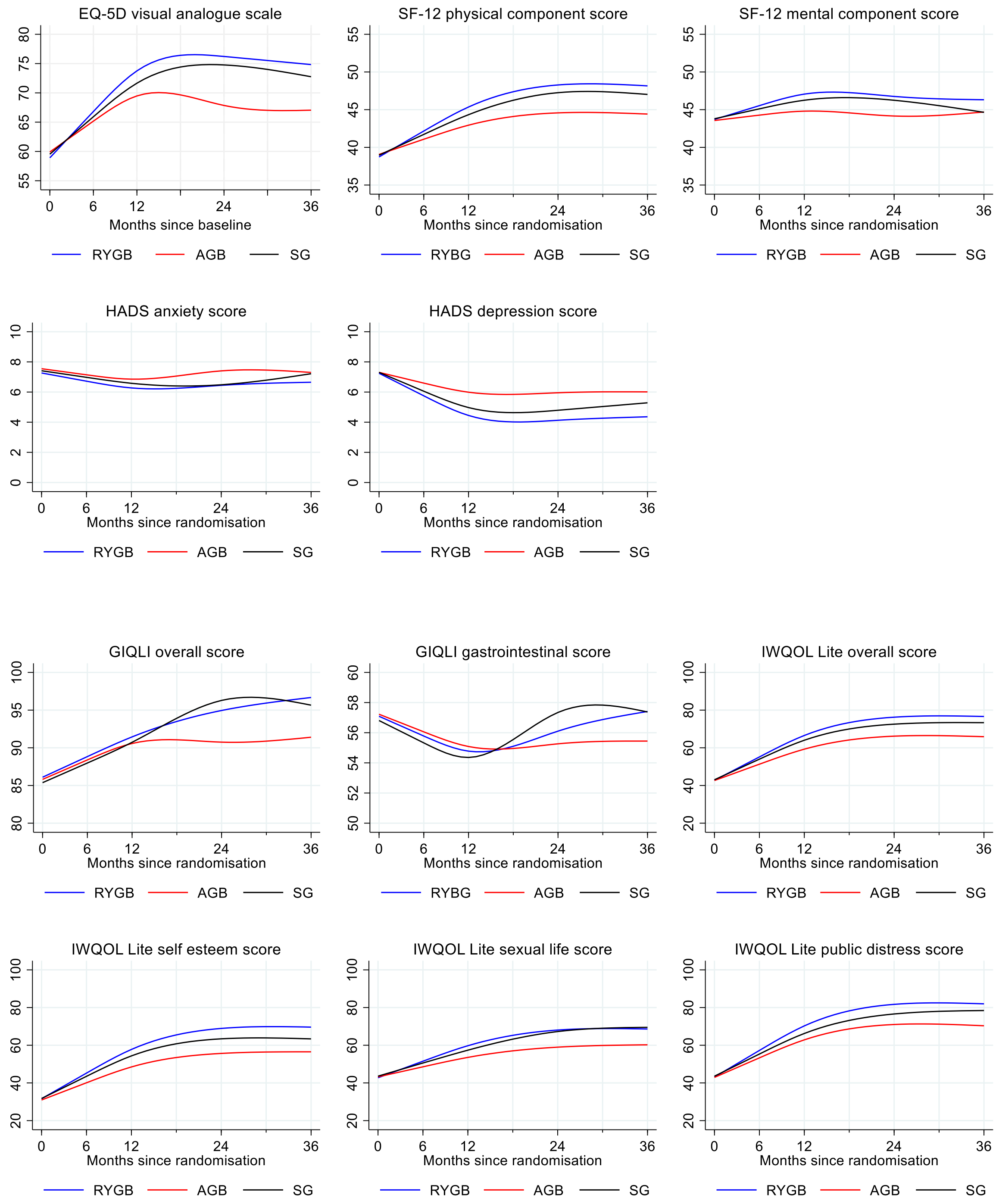

Trajectories are estimated from a regression model and represent the predicted mean scores.

RYGB = Roux-en-Y gastric bypass, AGB = adjustable gastric band, SG = sleeve gastrectomy, SF-12 = short-form 12, HADS = hospital anxiety and depression scale, GIQOL = gastrointestinal quality of life index, IWQOL = impact of weight on quality of life

Note: the scale on the y-axis differs across the quality-of-life measures

Table 24 Quality of life questionnaire responses at baseline and 3 years - intention-to-treat population

	Randomised to RYGB (n=462)	Randomised to AGB (n=464)	Randomised to SG (n=420)	Overall (n=1346)
SF-12 physical function				
Baseline[1]	39.1 (10.4)	38.8 (10.2)	39.3 (10.2)	39.1 (10.3)
3 years[2]	48.3 (10.9)	44.6 (12.0)	46.8 (11.1)	46.6 (11.4)
SF-12 role limitation (physical)				
Baseline[3]	39.7 (10.9)	39.2 (10.4)	39.8 (10.9)	39.6 (10.7)
3 years[4]	48.5 (10.8)	45.0 (11.6)	46.6 (11.0)	46.7 (11.2)
SF-12 bodily pain				
Baseline[5]	40.2 (12.2)	39.6 (12.1)	40.0 (12.1)	39.9 (12.1)
3 years[6]	47.3 (11.7)	43.2 (13.0)	45.1 (12.4)	45.2 (12.4)
SF-12 general health				
Baseline[7]	38.2 (11.0)	37.5 (11.3)	38.5 (11.2)	38.0 (11.2)
3 years[8]	48.0 (11.5)	43.1 (12.2)	46.1 (12.2)	45.7 (12.1)
SF-12 vitality				
Baseline[9]	42.1 (9.8)	41.8 (9.9)	42.9 (9.9)	42.2 (9.9)
3 years[10]	49.0 (11.3)	47.0 (10.7)	49.2 (11.1)	48.4 (11.1)
SF-12 social function				
Baseline[11]	41.0 (11.6)	40.2 (11.1)	40.9 (11.6)	40.7 (11.4)
3 years[12]	46.4 (11.6)	44.8 (12.1)	45.2 (12.5)	45.5 (12.0)
SF-12 role limitation (mental)				
Baseline[13]	40.5 (13.3)	40.4 (13.2)	40.5 (13.3)	40.4 (13.3)
3 years[14]	46.2 (12.2)	43.9 (13.3)	44.4 (12.7)	44.9 (12.7)
SF-12 mental health				
Baseline[15]	43.0 (10.7)	42.5 (10.6)	43.6 (10.0)	43.0 (10.5)
3 years[16]	47.2 (10.5)	45.2 (12.1)	46.3 (11.6)	46.2 (11.4)
IWQOL - physical				
Baseline[17]	43.1 (24.6)	42.7 (24.0)	42.5 (23.9)	42.8 (24.2)
3 years[18]	80.5 (22.4)	67.8 (26.4)	76.0 (21.6)	74.8 (24.2)
IWQOL - work				
Baseline[19]	59.6 (29.0)	57.2 (27.2)	61.1 (26.0)	59.2 (27.5)
3 years[20]	86.9 (19.4)	77.9 (26.7)	84.6 (20.9)	83.2 (22.8)
GIQLI - emotional				
Baseline[21]	8.8 (3.8)	8.5 (3.6)	8.5 (3.5)	8.6 (3.6)
3 years[22]	12.7 (3.6)	11.1 (4.1)	11.7 (3.6)	11.8 (3.8)

	Randomised to RYGB (n=462)	Randomised to AGB (n=464)	Randomised to SG (n=420)	Overall (n=1346)
GIQLI – social				
Baseline[23]	7.2 (2.6)	7.0 (2.6)	7.1 (2.6)	7.1 (2.6)
3 years[24]	8.2 (2.6)	8.1 (2.6)	8.7 (2.5)	8.3 (2.6)
GIQLI - physical				
Baseline[25]	9.7 (5.9)	9.2 (5.4)	9.7 (5.5)	9.5 (5.6)
3 years[26]	16.3 (6.2)	14.2 (6.8)	15.4 (6.5)	15.3 (6.5)
GIQLI - treatment				
Baseline[27]	2.6 (1.4)	2.6 (1.4)	2.8 (1.3)	2.7 (1.4)
3 years[28]	3.4 (1.0)	3.0 (1.4)	3.3 (1.1)	3.2 (1.2)

Data are presented as mean (standard deviation)

RYGB = Roux-en-Y gastric bypass, AGB = adjustable gastric band, SG = sleeve gastrectomy, SF-12 = short form 12, GIQOL = gastrointestinal quality of life index, IWQOL = impact of weight on quality of life

Missing data (RYGB, AGB, SG): [1]79 participants (24, 32, 23), [2]574 participants (191, 203, 180), [3]81 participants (23, 34, 24), [4]574 participants (191, 203, 180), [5]80 participants (24, 31, 25), [6]573 participants (191, 205, 177), [7]80 participants (24, 33, 23), [8]572 participants (191, 204, 177), [9]93 participants (29, 35, 29), [10]582 participants (193, 210, 179), [11]83 participants (26, 31, 26), [12]579 participants (193, 207, 179), [13]81 participants (24, 34, 23), [14]575 participants (192, 204, 179), [15]79 participants (24, 31, 24), [16]573 participants (191, 205, 177), [17]75 participants (22, 30, 23), [18]565 participants (190, 199, 176), [19]201 participants (59, 75, 67), [20]621 participants (203, 222, 196), [21]80 participants (24, 31, 25), [22]567 participants (188, 204, 175), [23]133 participants (42, 46, 45), [24]620 participants (199, 225, 196), [25]87 participants (25, 34, 28), [26]578 participants (190, 206, 182), [27]109 participants (32, 45, 32), [28]578 participants (191, 208, 179)

Table 25 Anxiety and depression as assessed using the HADS questionnaire at baseline and 3 years - intention-to-treat population

	Randomised to RYGB (n=462)	Randomised to AGB (n=464)	Randomised to SG (n=420)	Overall (n=1346)
Possible anxiety (score 8-10)				
Baseline	103/430 (24%)	88/418 (21%)	82/383 (21%)	273/1231 (22%)
3 years	41/261 (16%)	41/252 (16%)	36/232 (16%)	118/745 (16%)
Probable anxiety (score>10)				
Baseline	101/430 (23%)	121/418 (29%)	91/383 (24%)	313/1231 (25%)
3 years	52/261 (20%)	60/252 (24%)	55/232 (24%)	167/745 (22%)
Possible depression (score 8-10)				
Baseline	117/431 (27%)	103/418 (25%)	97/386 (25%)	317/1235 (26%)
3 years	29/261 (11%)	40/252 (16%)	28/231 (12%)	97/744 (13%)
Probable depression (score>10)				
Baseline	112/431 (26%)	103/418 (25%)	90/386 (23%)	305/1235 (25%)
3 years	29/261 (11%)	43/252 (17%)	32/231 (14%)	104/744 (14%)

Data are presented as n/N (%)

RYGB = Roux-en-Y gastric bypass, AGB = adjustable gastric band, SG = sleeve gastrectomy, HADS = hospital anxiety and depression scale

Table 26 Causes of deaths within 3 years of randomisation - intention-to-treat population

Randomised to RYGB (n=4)	Randomised to AGB (n=5)	Randomised to SG (n=2)
**Underwent surgery**		
• Global bowel ischemia (not related to RYGB)<br>• Myocardial fibrosis and fatty liver disease	• Ischaemic heart disease (n=2)<br>• Sepsis from soft tissue abscess (diabetic)<br>• Peritonitis due to leak from gastric band sutures	• Metastatic renal cell cancer
**Did not undergo metabolic bariatric surgery**		
• Pulmonary embolus and deep vein thrombosis<br>• Metastatic vulval cancer (deep vein thrombosis and gastrointestinal bleed)	• COVID-19 pneumonia	• Metastatic breast cancer

RYGB = Roux-en-Y gastric bypass, AGB = adjustable gastric band, SG = sleeve gastrectomy

Participants who underwent surgery received the allocated surgery

Table 27 Surgery-specific morbidity – participants who underwent surgery

	Received RYGB (n=389)	Received AGB (n=363)	Received SG (n=429)
**Surgery-related**			
Intra-abdominal haematoma requiring surgery or drain placement	1		3
Internal hernia (in meso-colic space) requiring repair	15		0
Hiatal hernia repair not at time of initial intervention	2		1
Adhesion obstruction requiring surgery +/- small bowel resection	4		
Anastomotic obstruction/perforation requiring revision surgery or dilatation at:	4		
Jejuno-jejunostomy[1]	1		
G-J anastomosis[2]	3		
Gastrointestinal leak requiring surgery and/or drain placement			
From sleeve staple line (fistula)			3
From G-J anastomosis	0		
From J-J anastomosis	0		
From band sutures		1	
Corrective surgery for maintaining the AGB[3]		16	
AGB removal (not revision)		22	
Revision surgery			
AGB to RYGB		10	
AGB to SG		2	
SG to RYGB			4
RYGB reversal to normal anatomy	0		
**Medically related**			
Need for total parenteral nutrition/enteral feeding	5	-	1
Malnutrition	2	-	
Neuropathy	-	-	-
Wernicke's encephalopathy	-	-	-
Hyperglycaemia		1	4
Hypoglycaemia	1	-	2
Abdominal pain	38	31	27
Diarrhoea	1	-	1
Electrolyte imbalance	10	5	16
Ketoacidosis	1	1	

	Received RYGB (n=389)	Received AGB (n=363)	Received SG (n=429)
Dehydration	1	-	2
Iron deficiency	44	18	46
High turnover osteopathy	1	-	-
Dry skin or hair	-	-	-
Oesophagitis	1	2	6
Death within 3 years	2	4	1

Data are number of events

RYGB = Roux-en-Y gastric bypass, AGB = adjustable gastric band, SG = sleeve gastrectomy

[1] Obstruction at one week, redone, [2] Stricture within a year, redone at 2 years; Ulcer perforation at G-J anastomosis at 10 months; G-J revision at 3 years, [3] Slippage, erosion, infection, and/or leak

Table 28 Adverse events following surgery: surgery to 30 days – safety population 3

	Received RYGB (n = 389)			Received AGB (n = 363)			Received SG (n = 429)			Overall (n = 1181)		
Event	Participants n (%)	Events (n)	SAE (n)	Participants n (%)	Events (n)	SAE (n)	Participants n (%)	Events (n)	SAE (n)	Participants n (%)	Events (n)	SAE (n)
**All events**	**112 (29%)**	**175**	**75/175**	**83 (23%)**	**120**	**37/120**	**110 (26%)**	**181**	**74/181**	**305 (26%)**	**476**	**186/476**
**Cardiac disorders**	**2 (1%)**	**2**	**1/2**	**1 (<1%)**	**1**	**1/1**	**3 (1%)**	**4**	**3/4**	**6 (1%)**	**7**	**5/7**
**Gastrointestinal disorders**	**34 (9%)**	**43**	**25/43**	**13 (4%)**	**14**	**8/14**	**28 (7%)**	**36**	**24/36**	**75 (6%)**	**93**	**57/93**
Abdominal pain	10 (3%)	10	7/10	3 (1%)	3	2/3	13 (3%)	13	11/13	26 (2%)	26	20/26
Vomiting	8 (2%)	9	3/9	1 (<1%)	1	1/1	3 (1%)	3	1/3	12 (1%)	13	5/13
**General disorders and administration site conditions**	**5 (1%)**	**5**	**2/5**	**14 (4%)**	**14**	**4/14**	**1 (<1%)**	**1**	**0/1**	**20 (2%)**	**20**	**6/20**
**Infections and infestations**	**51 (13%)**	**54**	**13/54**	**48 (13%)**	**53**	**13/53**	**45 (10%)**	**47**	**6/47**	**144 (12%)**	**154**	**32/154**
Wound infection	39 (10%)	39	6/39	38 (10%)	42	4/42	40 (9%)	41	1/41	117 (10%)	122	11/122
**Injury, poisoning and procedural complications**	**22 (6%)**	**28**	**13/28**	**11 (3%)**	**13**	**3/13**	**25 (6%)**	**35**	**20/35**	**58 (5%)**	**76**	**36/76**
Haemorrhage	5 (1%)	5	1/5	2 (1%)	2	0/2	6 (1%)	7	5/7	13 (1%)	14	6/14
Transfusion	5 (1%)	5	3/5	0 (0%)	0	0/0	8 (2%)	8	7/8	13 (1%)	13	10/13
Urinary retention	4 (1%)	4	1/4	1 (<1%)	1	0/1	8 (2%)	8	0/8	13 (1%)	13	1/13
**Metabolism and nutrition disorders**	**18 (5%)**	**22**	**7/22**	**6 (2%)**	**7**	**0/7**	**32 (7%)**	**35**	**8/35**	**56 (5%)**	**64**	**15/64**
Iron deficiency	14 (4%)	14	3/14	3 (1%)	4	0/4	14 (3%)	15	1/15	31 (3%)	33	4/33
Electrolyte imbalance	5 (1%)	5	2/5	2 (1%)	2	0/2	12 (3%)	12	5/12	19 (2%)	19	7/19

Event	Received RYGB (n = 389) Participants n (%)	Events (n)	SAE (n)	Received AGB (n = 363) Participants n (%)	Events (n)	SAE (n)	Received SG (n = 429) Participants n (%)	Events (n)	SAE (n)	Overall (n = 1181) Participants n (%)	Events (n)	SAE (n)
**Musculoskeletal and connective tissue disorders**	**1 (<1%)**	**1**	**0/1**	**3 (1%)**	**3**	**0/3**	**0 (0%)**	**0**	**0/0**	**4 (<1%)**	**4**	**0/4**
**Nervous system disorders**	**1 (<1%)**	**1**	**0/1**	**2 (1%)**	**2**	**2/2**	**0 (0%)**	**0**	**0/0**	**3 (<1%)**	**3**	**2/3**
**Renal and urinary disorders**	**0 (0%)**	**0**	**0/0**	**0 (0%)**	**0**	**0/0**	**2 (<1%)**	**2**	**2/2**	**2 (<1%)**	**2**	**2/2**
**Respiratory, thoracic and mediastinal disorders**	**0 (0%)**	**0**	**0/0**	**3 (1%)**	**4**	**3/4**	**2 (<1%)**	**2**	**2/2**	**5 (<1%)**	**6**	**5/6**
**Surgical and medical procedures**	**10 (3%)**	**17**	**14/17**	**7 (2%)**	**8**	**2/8**	**5 (1%)**	**8**	**5/8**	**22 (2%)**	**33**	**21/33**
**Vascular disorders**	**1 (<1%)**	**1**	**0/1**	**0 (0%)**	**0**	**0/0**	**4 (1%)**	**4**	**0/4**	**5 (<1%)**	**5**	**0/5**
**Eye disorders**	**0 (0%)**	**0**	**0/0**	**0 (0%)**	**0**	**0/0**	**1 (<1%)**	**1**	**1/1**	**1 (<1%)**	**1**	**1/1**
**Psychiatric disorders**	**0 (0%)**	**0**	**0/0**	**1 (<1%)**	**1**	**1/1**	**0 (0%)**	**0**	**0/0**	**1 (<1%)**	**1**	**1/1**
**Skin and subcutaneous tissue disorders**	**0 (0%)**	**0**	**0/0**	**0 (0%)**	**0**	**0/0**	**3 (1%)**	**3**	**1/3**	**3 (<1%)**	**3**	**1/3**

Events within a system organ class are listed where the event occurred in at least 12 participants (10% of the cohort who underwent surgery)

SAE – serious adverse event

Table 29 Adverse events following surgery: 30 days post-surgery to three years – safety population 3

Event	Received RYGB (n = 389) Participants n (%)	Events (n)	SAE (n)	Received AGB (n = 363) Participants n (%)	Events (n)	SAE (n)	Received SG (n = 429) Participants n (%)	Events (n)	SAE (n)	Overall (n = 1181) Participants n (%)	Events (n)	SAE (n)
**All events**	**157 (40%)**	**412**	**208/412**	**171 (47%)**	**443**	**247/443**	**154 (36%)**	**320**	**173/320**	**482 (41%)**	**1175**	**628/1175**
**Cardiac disorders**	**3 (1%)**	**3**	**2/3**	**7 (2%)**	**10**	**8/10**	**6 (1%)**	**6**	**5/6**	**16 (1%)**	**19**	**15/19**
**Gastrointestinal disorders**	**52 (13%)**	**91**	**52/91**	**38 (10%)**	**68**	**41/68**	**29 (7%)**	**43**	**26/43**	**119 (10%)**	**202**	**119/202**
Abdominal pain	19 (5%)	29	21/29	20 (6%)	28	16/28	11 (3%)	14	7/14	50 (4%)	71	44/71
Vomiting	15 (4%)	18	5/18	13 (4%)	13	10/13	5 (1%)	5	4/5	33 (3%)	36	19/36
**General disorders and administration site conditions**	**12 (3%)**	**12**	**3/12**	**41 (11%)**	**66**	**26/66**	**10 (2%)**	**11**	**5/11**	**63 (5%)**	**89**	**34/89**
Complication associated with device	0 (0%)	0	0/0	30 (8%)	48	20/48	0 (0%)	0	0/0	30 (3%)	48	20/48
Chest pain	4 (1%)	4	1/4	5 (1%)	6	2/6	6 (1%)	7	3/7	15 (1%)	17	6/17
**Hepatobiliary disorders**	**16 (4%)**	**18**	**9/18**	**6 (2%)**	**7**	**2/7**	**14 (3%)**	**20**	**10/20**	**36 (3%)**	**45**	**21/45**
Cholecystitis acute	10 (3%)	11	6/11	5 (1%)	5	1/5	11 (3%)	13	5/13	26 (2%)	29	12/29
Cholangitis	6 (1.5%)	6	2/6	1 (<1%)	1	0/1	6 (1%)	6	4/6	13 (1%)	13	6/13
**Immune system disorders**	**1 (<1%)**	**1**	**0/1**	**1 (<1%)**	**1**	**0/1**	**0 (0%)**	**0**	**0/0**	**2 (<1%)**	**2**	**0/2**
**Infections and infestations**	**24 (6%)**	**32**	**14/32**	**44 (12%)**	**62**	**30/62**	**24 (6%)**	**30**	**25/30**	**92 (8%)**	**124**	**69/124**
Wound infection	6 (2%)	7	2/7	13 (4%)	16	3/16	3 (1%)	3	1/3	22 (2%)	26	6/26
Respiratory tract infection	1 (<1%)	2	2/2	8 (2%)	9	5/9	6 (1%)	6	5/6	15 (1%)	17	12/17

Event	Received RYGB (n = 389) Participants n (%)	Events (n)	SAE (n)	Received AGB (n = 363) Participants n (%)	Events (n)	SAE (n)	Received SG (n = 429) Participants n (%)	Events (n)	SAE (n)	Overall (n = 1181) Participants n (%)	Events (n)	SAE (n)
Urinary tract infection	6 (2%)	7	0/7	8 (2%)	10	2/10	1 (<1%)	1	1/1	15 (1%)	18	3/18
**Injury, poisoning and procedural complications**	**25 (6%)**	**35**	**9/35**	**16 (4%)**	**20**	**6/20**	**35 (8%)**	**42**	**12/42**	**76 (6%)**	**97**	**27/97**
Fall	5 (1%)	5	1/5	7 (2%)	7	1/7	9 (2%)	9	2/9	21 (2%)	21	4/21
Musculoskeletal injury	6 (2%)	8	0/8	2 (1%)	3	0/3	8 (2%)	8	0/8	16 (1%)	19	0/19
**Metabolism and nutrition disorders**	**34 (9%)**	**37**	**7/37**	**18 (5%)**	**18**	**3/18**	**33 (8%)**	**35**	**4/35**	**85 (7%)**	**90**	**14/90**
Iron deficiency	27 (7%)	28	3/28	14 (4%)	14	1/14	29 (7%)	31	1/31	70 (6%)	73	5/73
**Musculoskeletal and connective tissue disorders**	**3 (1%)**	**3**	**0/3**	**10 (3%)**	**13**	**6/13**	**8 (2%)**	**10**	**1/10**	**21 (2%)**	**26**	**7/26**
**Nervous system disorders**	**10 (3%)**	**11**	**4/11**	**7 (2%)**	**15**	**8/15**	**7 (2%)**	**7**	**2/7**	**24 (2%)**	**33**	**14/33**
**Renal and urinary disorders**	**6 (2%)**	**10**	**3/10**	**3 (1%)**	**6**	**4/6**	**2 (<1%)**	**2**	**0/2**	**11 (1%)**	**18**	**7/18**
**Respiratory, thoracic and mediastinal disorders**	**6 (2%)**	**7**	**1/7**	**4 (1%)**	**5**	**3/5**	**7 (2%)**	**8**	**4/8**	**17 (1%)**	**20**	**8/20**
**Surgical and medical procedures**	**71 (18%)**	**108**	**78/108**	**82 (23%)**	**130**	**96/130**	**59 (14%)**	**75**	**54/75**	**212 (18%)**	**313**	**228/313**
Joint arthroplasty	12 (3%)	12	10/12	15 (4%)	15	11/15	14 (3%)	16	9/16	41 (3%)	43	30/43
Gastric banding reversal	0 (0%)	0	0/0	30 (8%)	36	22/36	0 (0%)	0	0/0	30 (3%)	36	22/36
Cholecystectomy	6 (2%)	6	5/6	4 (1%)	4	4/4	10 (2%)	10	9/10	20 (2%)	20	18/20
Gastric bypass	0 (0%)	0	0/0	10 (3%)	10	8/10	4 (1%)	4	2/4	14 (1%)	14	10/14
Knee arthroplasty	6 (2%)	6	6/6	5 (1%)	6	5/6	3 (1%)	3	3/3	14 (1%)	15	14/15
**Vascular disorders**	**11 (3%)**	**12**	**2/12**	**3 (1%)**	**3**	**1/3**	**6 (1%)**	**6**	**3/6**	**20 (2%)**	**21**	**6/21**
**Blood and lymphatic system disorders**	**4 (1%)**	**6**	**2/6**	**1 (<1%)**	**1**	**1/1**	**1 (<1%)**	**1**	**1/1**	**6 (1%)**	**8**	**4/8**

	Received RYGB (n = 389)			Received AGB (n = 363)			Received SG (n = 429)			Overall (n = 1181)		
**Event**	**Participants n (%)**	**Events (n)**	**SAE (n)**	**Participants n (%)**	**Events (n)**	**SAE (n)**	**Participants n (%)**	**Events (n)**	**SAE (n)**	**Participants n (%)**	**Events (n)**	**SAE (n)**
**Ear and labyrinth disorders**	**1 (<1%)**	**1**	**0/1**	**1 (<1%)**	**1**	**1/1**	**0 (0%)**	**0**	**0/0**	**2 (<1%)**	**2**	**1/2**
**Eye disorders**	**1 (<1%)**	**1**	**0/1**	**0 (0%)**	**0**	**0/0**	**0 (0%)**	**0**	**0/0**	**1 (<1%)**	**1**	**0/1**
**Neoplasms benign, malignant and unspecified (incl. cysts and polyps)**	**1 (<1%)**	**1**	**1/1**	**1 (<1%)**	**1**	**1/1**	**4 (1%)**	**6**	**6/6**	**6 (1%)**	**8**	**8/8**
**Pregnancy, puerperium and perinatal conditions**	**7 (2%)**	**18**	**18/18**	**4 (1%)**	**6**	**6/6**	**5 (1%)**	**7**	**7/7**	**16 (1%)**	**31**	**31/31**
Delivery	5 (1%)	5	5/5	5 (1%)	5	5/5	5 (1%)	5	5/5	14 (1%)	14	14/14
**Psychiatric disorders**	**2 (1%)**	**2**	**0/2**	**4 (1%)**	**6**	**2/6**	**4 (1%)**	**5**	**4/5**	**10 (1%)**	**13**	**6/13**
**Reproductive system and breast disorders**	**3 (1%)**	**3**	**3/3**	**1 (<1%)**	**1**	**1/1**	**4 (1%)**	**6**	**4/6**	**8 (1%)**	**10**	**8/10**
**Skin and subcutaneous tissue disorders**	**0 (0%)**	**0**	**0/0**	**2 (1%)**	**3**	**1/3**	**0 (0%)**	**0**	**0/0**	**2 (<1%)**	**3**	**1/3**

Events within a system organ class are listed where the event occurred in at least 12 participants (10% of the cohort who underwent surgery)

SAE – serious adverse event

Table 30 Intraclass correlations for outcomes measured at multiple post randomisation time points

Outcome	Study centre	Repeated measurements on a participant
Total weight loss	0.2451	0.6959
BMI	0.2418	0.6978
EQ-5D utility score (primary analysis)[1]	0.0034	0.4815
EQ-5D visual analogue scale	0.0184	0.5218
SF-12 physical component[2]	0.1300	0.5713
SF-12 mental component[2]	0.0618	0.5434
HADS anxiety score	0.0200	0.5733
HADS depression score	0.0375	0.5731
GIQLI overall score	0.0413	0.6179
GIQLI gastrointestinal score	0.0034	0.5377
IWQOL overall score[2]	0.2028	0.6619
IWQOL self-esteem score[2]	0.1514	0.6169
IWQOL sexual life score[2]	0.1427	0.6195
IWQOL public distress score[2]	0.1687	0.6269
HbA1c	0.0673	0.6477
Fasting glucose[2]	0.0914	0.5778
Triglycerides	0.0264	0.5424
HDL-C	0.0341	0.4605
Total cholesterol	0.0031	0.5570
Ferritin	0.0153	0.5908
Serum iron	0.0104	0.4884
Folate	0.0476	0.6104
Vitamin B12	0.0975	0.4745
25 hydroxyvitamin D	0.0204	0.6459
Parathyroid hormone[2]	0.1744	0.5986
Haemoglobin	0.0144	0.5226
Calcium	0.0148	0.4399
ALT	0.0236	0.4816
ALP	0.0153	0.5204
Creatinine	0.0155	0.5824
Energy intake (kcal/day)	0.0149	0.2373
Dietary fibre (g/day)	0.0855	0.2803
% energy from protein	0.0302	0.2286

Outcome	Study centre	Repeated measurements on a participant
% energy from fat	0.0087	0.1864
% energy from carbohydrate	0.0040	0.3244
Total folate (µg/day)	0.0089	0.2029
Vitamin B1 (mg/day)	0.0047	0.3658
Vitamin B12 (mg/day)	0.0042	0.0795
Vitamin E (mg/day)	0.0042	0.2052
Calcium (mg/day)	0.0081	0.2195
Iron (mg/day)	0.0111	0.2859
Binge eating score[2]	0.1346	0.5893
Sleepiness	0.0108	0.5638

[1] In the sensitivity analyses the intraclass correlation for centre ranged from 0.0013 (excluding crossovers and those who didn't have surgery and grouping participants by surgery received) to 0.0105 (estimating outcomes post-surgery) and the intraclass correlation for repeated measurements on a participant at a site ranged from 0.4734 (grouping participants by surgery received) to 0.4948 (excluding crossovers and those who didn't have surgery)

[2] Missing baseline data were imputed for this outcome, the ICC was estimated from the model fitted to the imputed data

Table 31 Estimated costs (including intervention costs) and QALYs (primary analysis)

	Costs			QALYs		
Allocated surgery	Mean costs (£) per participant (95% CI)	Cost per participant vs AGB (98% CI)	Cost per participant vs RYGB (98% CI)	Mean QALYs (95% CI)	Incremental QALYs per participant vs AGB (98% CI)	Incremental QALYs per participant vs RYGB (98% CI)
AGB	7357 (6865, 7905)			1.82 (1.75, 1.90)		
SG	7695 (7143, 8451)	337 (-626, 1396)	-574 (-1486, 526)	1.95 (1.88, 2.03)	0.13 (0.02, 0.27)	-0.07 (-0.20, 0.05)
RYGB	8268 (7786, 8813)	911 (76, 1813)		2.02 (1.95, 2.09)	0.20 (0.08, 0.31)	

RYGB = Roux-en-Y gastric bypass, AGB = adjustable gastric band, SG = sleeve gastrectomy, QALY = quality-adjusted life year, CI = confidence interval

Table 32 Impact of sensitivity analyses on estimated costs including intervention costs

	Gamma regression		Assuming costs data are incomplete when weight is missing and using inverse probability of censoring weighting to address selection bias after censoring participants with missing weight data		Per protocol analysis: only including participants that received the surgery they were allocated to (compliers)		Estimating the effect of performing surgery at t=0 among compliers assuming stable effects over time	
Allocated surgery	Cost per participant vs AGB (98% CI)	Cost per participant vs RYGB (98% CI)	Cost per participant vs AGB (98% CI)	Cost per participant vs RYGB (98% CI)	Cost per participant vs AGB (98% CI)	Cost per participant vs RYGB (98% CI)	Cost per participant vs AGB (98% CI)	Cost per participant vs RYGB (98% CI)
SG	425 (-502, 1310)	-523 (-1,426, 375)	328 (-649, 1,346)	-431 (-1469, 651)	371 (-455, 1,154)	-676 (-1,616, 290)	640 (-321, 1,476)	-871 (-1,707, -60)
RYGB	948 (142, 1787)	-	759 (-103, 1,674)	-	1,047 (18, 1,946)	-	1,544 (586, 2585)	-

RYGB = Roux-en-Y gastric bypass, AGB = adjustable gastric band, SG = sleeve gastrectomy, CI = confidence interval

Results from the primary analysis are shown in Table 31

Table 33 Impact of sensitivity analyses on estimated difference in health-related quality of life

Allocated surgery	Beta-one-inflated regression		Mixed effect regression with a spline for time and a random intercept		Mixed effect regression with a random slope and intercept and time modelled using a spline		Mixed effect regression with time modelled as a categorical variable and a random intercept	
	Incremental QALYs per participant vs AGB (98% CI)	Incremental QALYs per participant vs RYGB (98% CI)	Incremental QALYs per participant vs AGB (98% CI)	Incremental QALYs per participant vs RYGB (98% CI)	Incremental QALYs per participant vs AGB (98% CI)	Incremental QALYs per participant vs RYGB (98% CI)	Incremental QALYs per participant vs AGB (98% CI)	Incremental QALYs per participant vs RYGB (98% CI)
SG	0.10 (0.01, 0.21)	-0.03 (-0.13, 0.07)	0.10 (-0.01, 0.21)	-0.08 (-0.20, 0.03)	0.10 (-0.02, 0.22)	-0.08 (-0.19, 0.04)	0.13 (0.00, 0.27)	-0.07 (-0.20, 0.04)
RYGB	0.13 (0.02, 0.24)		0.18 (0.06, 0.29)		0.18 (0.07, 0.30)		0.20 (0.08, 0.32)	

Allocated surgery	Per protocol analysis: only including participants that received the surgery they were allocated to (compliers)		Estimating the effect of performing surgery at t=0 among compliers assuming stable effects over time	
	Incremental QALYs per participant vs AGB (98% CI)	Incremental QALYs per participant vs RYGB (98% CI)	Incremental QALYs per participant vs AGB (98% CI)	Incremental QALYs per participant vs RYGB (98% CI)
SG	0.14 (0.03, 0.27)	-0.10 (-0.24, 0.04)	0.17 (0.04, 0.31)	-0.09 (-0.20, 0.05)
RYGB	0.24 (0.13, 0.38)		0.28 (0.14, 0.40)	

RYGB = Roux-en-Y gastric bypass, AGB = adjustable gastric band, SG = sleeve gastrectomy, QALY = quality-adjusted life year, CI = confidence interval

Results from the primary analysis are shown in Table 31

Table 34 Impact of alternative discount rates

	Costs, 0% discount rate			Costs, 5% discount rate		
Allocated surgery	Mean costs (£) per participant (95% CI)	Cost per participant vs AGB (98% CI)	Cost per participant vs RYGB (98% CI)	Mean costs (£) per participant (95% CI)	Cost per participant vs AGB (98% CI)	Cost per participant vs RYGB (98% CI)
AGB	7,510 (7,006, 8,074)			7296 (6809, 7838)		
SG	7,843 (7,270, 8,524)	333 (-665, 1,425)	-557 (-1,492, 569)	7635 (7090, 8282)	339 (-609, 1,385)	-580 (-1,486, 450)
RYGB	8,400 (7,907, 8,958)	890 (21, 1,820)		8215 (7736, 8755)	919 (98, 1,810)	

	QALYs, 0% discount rate			QALYs, 5% discount rate		
Allocated surgery	Mean QALYs (95% CI)	Incremental QALYs per participant vs AGB (98% CI)	Incremental QALYs per participant vs RYGB (98% CI)	Mean QALYs (95% CI)	Incremental QALYs per participant vs AGB (98% CI)	Incremental QALYs per participant vs RYGB (98% CI)
AGB	1.88 (1.81, 1.96)			1.79 (1.72, 1.87)		
SG	2.02 (1.94, 2.10)	0.14 (0.02, 0.28)	-0.07 (-0.21, 0.05)	1.92 (1.85, 2.00)	0.13 (0.02, 0.26)	-0.07 (-0.20, 0.05)
RYGB	2.09 (2.02, 2.17)	0.21 (0.09, 0.33)		1.99 (1.92, 2.06)	0.20 (0.08, 0.31)	

RYGB = Roux-en-Y gastric bypass, AGB = adjustable gastric band, SG = sleeve gastrectomy, QALY = quality-adjusted life year, CI = confidence interval

Results from the primary analysis, which used discount rates of 3.5% in line with UK cost-effectiveness guidelines, are shown in Table 31

Table 35 Estimated costs (including intervention costs) and QALYs - comparing participants randomised before and after start of COVID-19 pandemic

	Costs before COVID-19			Costs after start COVID-19		
Allocated surgery	Mean costs (£) per participant (95% CI)	Cost per participant vs AGB (98% CI)	Cost per participant vs RYGB (98% CI)	Mean costs (£) per participant (95% CI)	Cost per participant vs AGB (98% CI)	Cost per participant vs RYGB (98% CI)
AGB	8034 (7353, 8819)			6469 (5898, 7226)		
SG	8260 (7636, 9040)	226 (-1025, 1314)	-18 (-1070, 1314)	7411 (6544, 8458)	942 (-474, 2342)	-796 (-2189, 618)
RYGB	8277 (7685, 8930)	243 (-867, 1311)		8207 (7418, 9182)	1738 (520, 3049)	

	QALYs before COVID-19			QALYS after start COVID-19		
Allocated surgery	Mean QALYs (95% CI)	Incremental QALYs per participant vs AGB (98% CI)	Incremental QALYs per participant vs RYGB (98% CI)	Mean QALYs (95% CI)	Incremental QALYs per participant vs AGB (98% CI)	Incremental QALYs per participant vs RYGB (98% CI)
AGB	1.83 (1.75, 1.91)			1.81 (1.74, 1.88)		
SG	1.95 (1.87, 2.04)	0.12 (-0.01, 0.24)	-0.09 (-0.21, 0.04)	1.93 (1.85, 2.01)	0.12 (0.02, 0.24)	-0.03 (-0.14, 0.11)
RYGB	2.04 (1.95, 2.12)	0.21 (0.09, 0.33)		1.96 (1.87, 2.02)	0.14 (0.02, 0.28)	

RYGB = Roux-en-Y gastric bypass, AGB = adjustable gastric band, SG = sleeve gastrectomy, QALY = quality-adjusted life year, CI = confidence interval

Figure 5 Expected incremental net monetary benefit

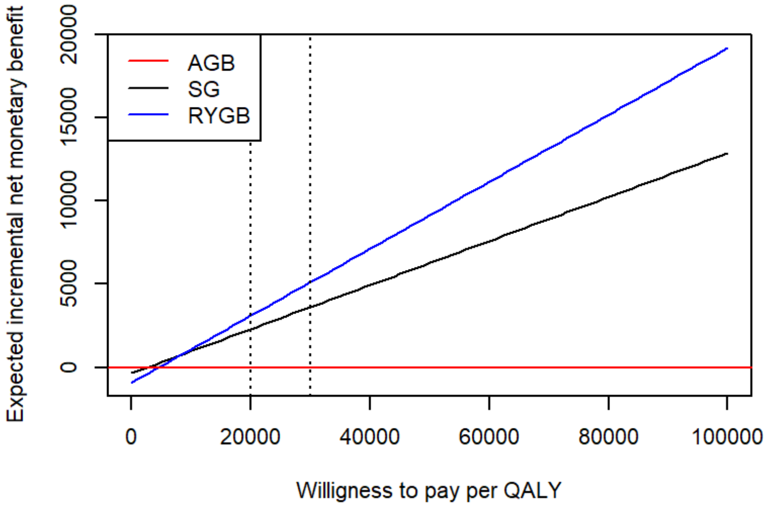

Results are based on *n* = 500 bootstrap samples using adjustable gastric banding as the reference, willingness to pay per QALY and the expected incremental net monetary benefit are expressed in 2021 Great British Pounds (GBP). The dashed vertical lines represent the £20,000 and £30,000 per QALY thresholds generally used by NICE. AGB = adjustable gastric banding; RYGB = Roux-en-Y gastric bypass; SG = sleeve gastrectomy; QALY = quality-adjusted life year.

Figure 6 Cost-effectiveness acceptability curves

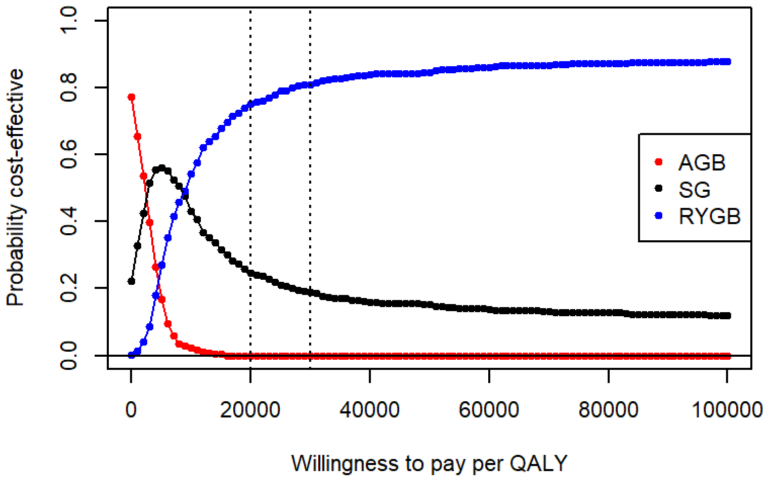

Results are based on n = 500 bootstrap samples using adjustable gastric banding as the reference, willingness to pay per QALY is expressed in 2021 Great British Pounds (GBP). The dashed vertical lines represent the £20,000 and £30,000 per QALY thresholds generally used by NICE. AGB = adjustable gastric banding; RYGB = Roux-en-Y gastric bypass; SG = sleeve gastrectomy; QALY = quality-adjusted life year.

Figure 7 Expected incremental net monetary benefit by body mass index and diabetes status at baseline

a) By body mass index

body mass index<40kg/m^2 | body mass index 40–50kg/m^2 | body mass index>50kg/m^2

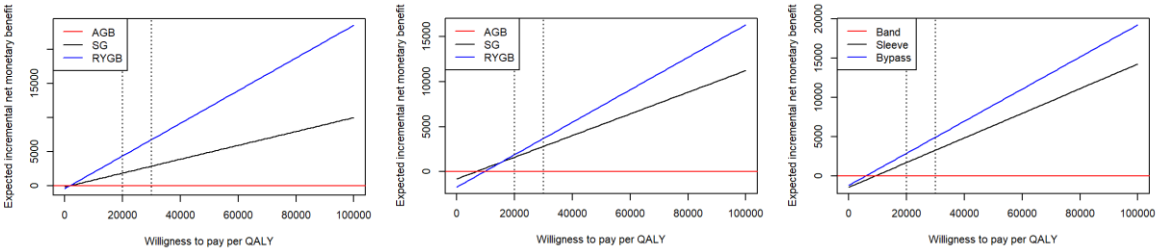

b) By diabetic status

no diabetes | diabetic

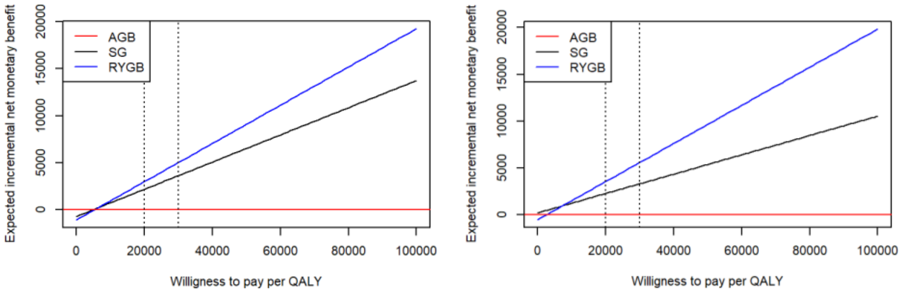

Results are based on n = 500 bootstrap samples using adjustable gastric banding as the reference, willingness to pay per QALY and the expected incremental net monetary benefit are expressed in 2021 Great British Pounds (GBP). The dashed vertical lines represent the £20,000 and £30,000 per QALY thresholds generally used by NICE. AGB = adjustable gastric banding; RYGB = Roux-en-Y gastric bypass; SG = sleeve gastrectomy; QALY = quality-adjusted life year.

Figure 8 Expected incremental net monetary benefit by surgery received

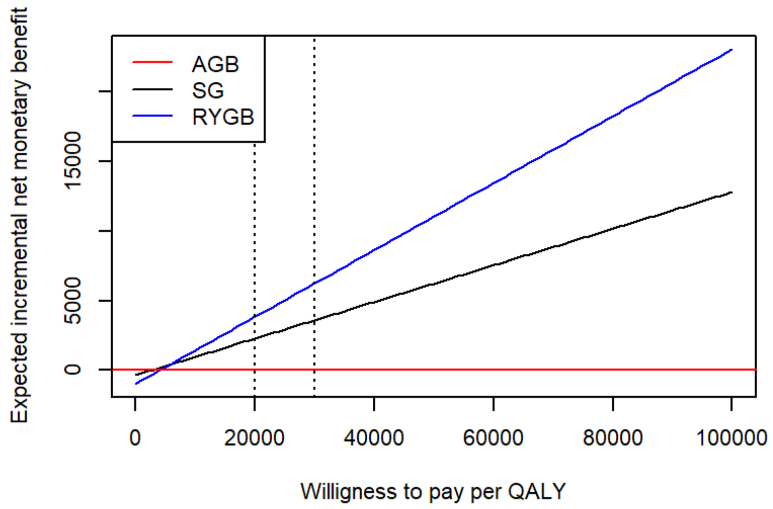

Results are based on n = 500 bootstrap samples using adjustable gastric banding as the reference, willingness to pay per QALY and the expected incremental net monetary benefit are expressed in 2021 Great British Pounds (GBP). The dashed vertical lines represent the £20,000 and £30,000 per QALY thresholds generally used by NICE. AGB = adjustable gastric banding; RYGB = Roux-en-Y gastric bypass, SG = sleeve gastrectomy; QALY = quality-adjusted life year.

Figure 9 Expected incremental net monetary benefit using a binary indicator to address a potential time-trend bias

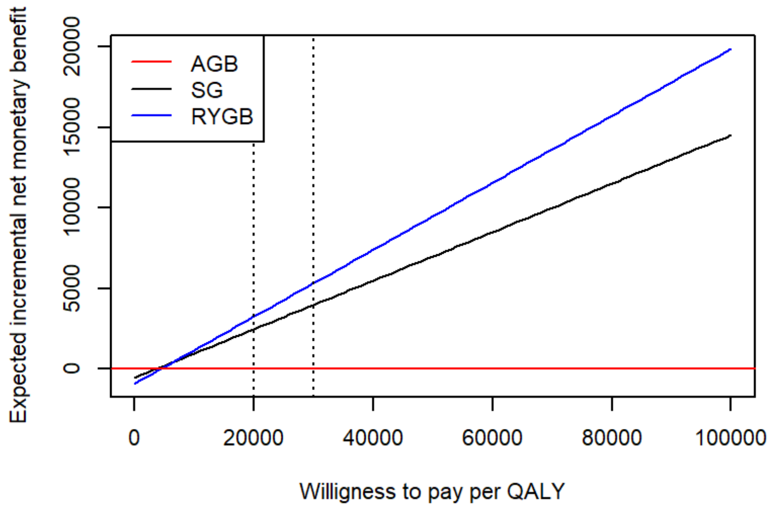

Results are based on n = 500 bootstrap samples using adjustable gastric banding as the reference, willingness to pay per QALY and the expected incremental net monetary benefit are expressed in 2021 Great British Pounds (GBP). The dashed vertical lines represent the £20,000 and £30,000 per QALY thresholds generally used by NICE. AGB = adjustable gastric banding; RYGB = Roux-en-Y gastric bypass; SG = sleeve gastrectomy; QALY = quality-adjusted life year.

Figure 10 Expected incremental net monetary benefit using a more recent mapping algorithm for deriving health-related quality of life

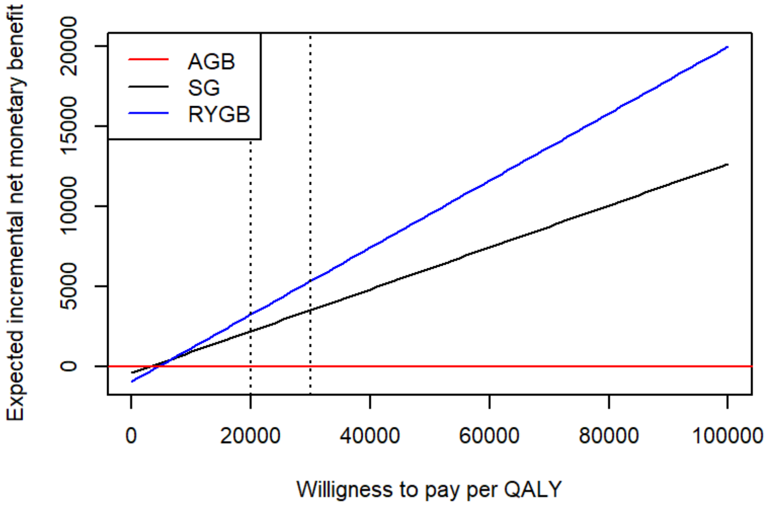

Results are based on n = 500 bootstrap samples using adjustable gastric banding as the reference, willingness to pay per QALY and the expected incremental net monetary benefit are expressed in 2021 Great British Pounds (GBP). The mapping algorithm is described in Wailoo et al. (https://pubmed.ncbi.nlm.nih.gov/33840435/) The dashed vertical lines represent the £20,000 and £30,000 per QALY thresholds generally used by NICE. AGB = adjustable gastric banding; RYGB = Roux-en-Y gastric bypass; SG = sleeve gastrectomy; QALY = quality-adjusted life year.

Figure 11 Expected incremental net monetary benefit using intervention costs as reported by NHS Cost Collection

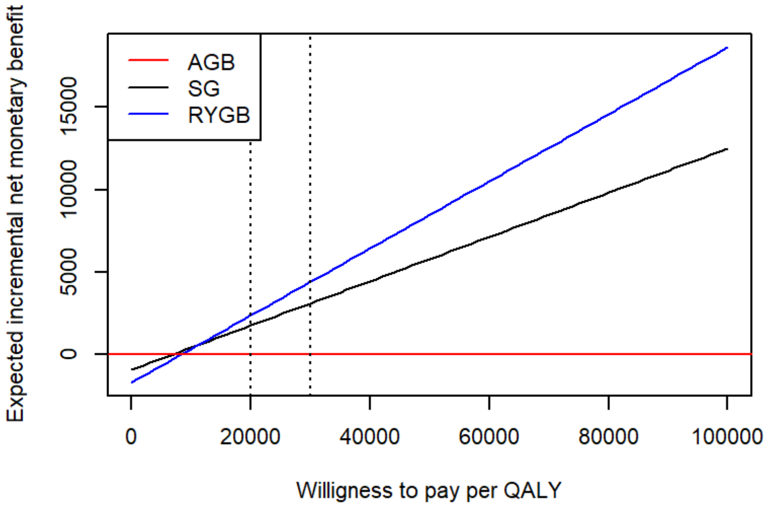

Results are based on n = 500 bootstrap samples using adjustable gastric banding as the reference, willingness to pay per QALY and the expected incremental net monetary benefit are expressed in 2021 Great British Pounds (GBP). The dashed vertical lines represent the £20,000 and £30,000 per QALY thresholds generally used by NICE. AGB = adjustable gastric banding; RYGB = Roux-en-Y gastric bypass; SG = sleeve gastrectomy; QALY = quality-adjusted life year.
